# Evaluation of the efficacy, safety and influencing factors of concomitant and sequential administration of viral respiratory infectious disease vaccines: a systematic review and meta-analysis

**DOI:** 10.3389/fimmu.2023.1259399

**Published:** 2023-12-21

**Authors:** Dafeng Lu, Yifang Han, Ruowei Xu, Mingke Qin, Jianwei Shi, Caihong Zhang, Jinhai Zhang, Fuqiang Ye, Zhenghan Luo, Yuhe Wang, Chunfang Wang, Chunhui Wang

**Affiliations:** ^1^ Department of Infectious Disease Prevention and Control, Nanjing Bioengineering (Gene) Technology Center for Medicines, Nanjing, China; ^2^ Department of Infectious Disease Prevention and Control, Quzhou Center for Disease Prevention and Control, Quzhou, China; ^3^ School of Public Health, Nanjing Medical University, Nanjing, China; ^4^ College of Life Science, Nanjing Normal University, Nanjing, China; ^5^ Department of Occupational Health, Third Military Medical University, Chongqing, China; ^6^ Department of Neurosurgery, Nanjing Brain Hospital Affiliated to Nanjing Medical University, Nanjing, China; ^7^ School of Public Health, Bengbu Medical College, Bengbu, China; ^8^ School of Pharmacy, Nanjing University of Chinese Medicine, Nanjing, China

**Keywords:** vaccine, concomitant administration, COVID-19, seasonal influenza, vaccine efficacy

## Abstract

**Background:**

There is no clear conclusion on the immunogenicity and adverse events of concomitant administration the viral respiratory infectious disease vaccines. We aimed to evaluate the impact of concomitant administering viral respiratory infectious disease vaccines on efficiencies, safety and influencing factors.

**Methods:**

This meta-analysis included studies from PubMed, Embase, Cochrane Central Register of Clinical Trials, Web of Science, WHO COVID-19 Research, and ClinicalTrials.gov databases. Randomized controlled trials of the adult participants concomitant administered with viral respiratory infectious disease vaccine and other vaccines were included. The main outcomes were the seroconversion rate and seroprotection rate of each vaccine. Used the Mantel–Haenszel fixed effects method as the main analysis to estimate the pooled RRs and the corresponding 95% confidence intervals. The risk of bias for each trial was assessed using the Cochrane Handbook for Systematic Reviews of Interventions, while evidence certainty was evaluated using the Grading of Recommendations Assessment, Development, and Evaluation system.

**Results:**

A total of 21 studies comprising 14060 participants with two types of vaccines were retained for the meta-analysis. Concomitant immunization reduced the geometric mean titer (RR: 0.858, 95% CI: (0.785 to 0.939)) and the geometric mean fold rise (0.754 (0.629 to 0.902)) in the SARS-COV-2 vaccine group but increased the seroconversion rate (1.033 (1.0002 to 1.067)) in the seasonal influenza vaccine group. Concomitant administration were influenced by the type of vaccine, adjuvant content, booster immunization, and age and gender of the recipient.

**Conclusion:**

This meta-analysis suggested that the short-term protection and safety of concomitant administered were effective. Appropriate adjuvants, health promotion and counselling and booster vaccines could improve the efficiency and safety of Concomitant vaccination.

**Systematic review registration:**

https://www.crd.york.ac.uk/PROSPERO/, identifier CRD42022343709.

## Introduction

The coronavirus disease 2019 (COVID-19) pandemic, caused by severe acute respiratory syndrome coronavirus 2 (SARS-CoV-2), has become a pressing global crisis and has led to 6.5 million deaths worldwide. The SARS-CoV-2 vaccines have been successfully in inducing the neutralizing humoral and cellular immunity against the virus, thus reducing infections, hospitalizations, and deaths in clinical trials (1, 2). Safe and effective vaccines are considered viable to end the pandemic (3).

There are significant concerns that the COVID‐19 pandemic may overlap with other respiratory viruses, particularly seasonal influenza. For instance, Europe experienced a new avian influenza epidemic in June 2022 and the United States has seen an unprecedented poultry H5N1 infection, with a high zoonotic spillage risk (4, 5). These instances highlight the challenge of overlapping the COVID-19 epidemic with influenza. Concomitant vaccination is recommended for people traveling to epidemic areas with other infectious diseases or as a planned immunization for infants and children, especially during the immunization season, to reduce the burden on healthcare services (6). Vaccine concomitant administration reduces the number of hospital visits, thereby reducing stress and inconvenience for children and parents (7).

Studies on concomitant vaccination against viral respiratory infectious diseases have focused on the seasonal influenza vaccine (SIV) administered with other vaccines in adults (8, 9). With the increasing cases of COVID-19 and the widespread development of SARS-COV-2 vaccines, studies on the concomitant vaccination with SARS-COV-2 vaccines are gradually being reported (6, 10). However, due to the limited sample size, the efficacy evaluation of concomitant vaccination in randomized clinical trials (RCTs) is based on the “non-inferiority criterion,” which is ambiguous, making accurate assessment difficult. There is no clear conclusion on the immunogenicity and Ads of concomitant vaccination with viral respiratory infectious disease vaccines (VRIDVs).

Currently, there is no meta-analysis on the concomitant administration of VRIDVs in adults. This study, therefore, conducted a systematic review and meta-analysis on the differences in immunogenicity and ADs between concomitant and sequential VRIDVs administration.

## Methods

### Search strategy and selection criteria

We conducted a systematic review and network meta-analysis according to the Preferred Reporting Items for Systematic Reviews and Meta-Analyses (PRISMA) guidelines (**Supplementary Data Sheet 1 (eTable 1)**) (11). The review protocol was prospectively registered with the PROSPERO ID CRD42022343709.

We searched PubMed, Embase, Cochrane Central Register of Clinical Trials, Web of Science, WHO COVID-19 Research, and ClinicalTrials.gov databases from inception to September 10, 2022. The search was conducted in English, and the key search terms were “Concomitant vaccination,” “Concomitant administration,” “Concomitant immunization,” AND (“SARS-CoV-2 Vaccine,” OR “Influenza Vaccine,” OR “Viral Vaccine” (**Supplementary Data Sheet 1 (eTable 2)**).

We used a two-stage approach for literature screening: by checking the title and abstract and then going through the full-text article. Two researchers (CH and DF) independently screened the title, abstract, and full text of each article, and the discrepancies were resolved through consensus with a third researcher (YF). Studies included in the meta-analysis were those evaluating concomitant administration of adults who received VRIDV, followed by the outcome, including immunogenicity and adverse events (ADs) of the concomitant administered and sequential/alone groups in RCTs. However, studies with unreported or ambiguous outcomes of immunological efficacy, no focus on VRIDVs, and those involving children or infants were excluded from the meta-analysis. In cases where the study lacked available data, we requested additional information from the corresponding authors, upon which the study was excluded if the data were not provided.

### Publication bias assessment and sensitivity analysis

The Egger regression test with a funnel plot was used to assess the publication bias in the meta-analysis of ≥8 groups, whereby p < 0.05 indicated significant asymmetry and publication bias. Moreover, sensitivity analysis was used to remove each cohort individually from the meta-analysis.

### Quality assessment

The risk of bias for each trial was assessed using the Cochrane Handbook for Systematic Reviews of Interventions (12–14), while evidence certainty was evaluated using the Grading of Recommendations Assessment, Development, and Evaluation (GRADE) system (15). Two researchers (DF and MK) independently assessed the risk of bias in individual studies and the GRADE system. Discrepancies were resolved by consensus and arbitration among the authors (DF, CH, YF, MK).

### Data analysis

Data on the study characteristics comprised the study setting, outcomes, study design, sample size, dropout or non-response rates, and inclusion and exclusion criteria. Participant data included age, gender, and vaccine history. Intervention-related data included the vaccine type and dosing schedule. Outcome-related data comprised the assay type, antibody measured, method of measurement, and intervals of sample collection. The categorical outcomes of the arm subgroups from multi-arm studies were combined, and the continuous outcomes from the large sample size arm were used to represent the study. Two independent researchers (DF and MK) assessed the extracted data.

We analyzed the data provided to compare the efficacy and safety of VRIDVs and reported the findings as relative risks (RRs) for binary outcomes, including seroconversion rate (SCR), seroprotection rate (SPR) and ADs. The main outcomes were the SPR and SCR of each vaccine. Since there is no common evaluation standard for the immunogenicity of SARS-CoV-2 vaccines (16), we combined the geometric mean titer (GMT), geometric mean concentrations (GMCs) and geometric mean fold rises (GMFRs) in the meta-analysis and reported as the GMT through the ratio of means (ROMs) method.

We used the Mantel–Haenszel fixed effects method as the main analysis to estimate the pooled RRs and the corresponding 95% confidence intervals (CIs), while the random-effects model was used for the heterogeneity analysis at I^2^ >50%. Statistical heterogeneities of the results of the included studies were determined using the DerSimonian-Laird estimator, which assessed the between-study variance I^2^ statistic. Heterogeneity was considered low, moderate, or high if estimated I^2^ was below 25%, between 25% and 50%, and above 50%, respectively. Subgroup analysis and meta-regressions were performed to examine the relationship between the outcome and baseline characteristics of the cohorts. The subgroup analysis was undertaken if at least 6 cohorts were available. All analyses were conducted on R (version 4.1.1) using the meta and metafor packages. A two-sided P value < 0.05 indicated statistical significance.

## Result

**Figure 1:**
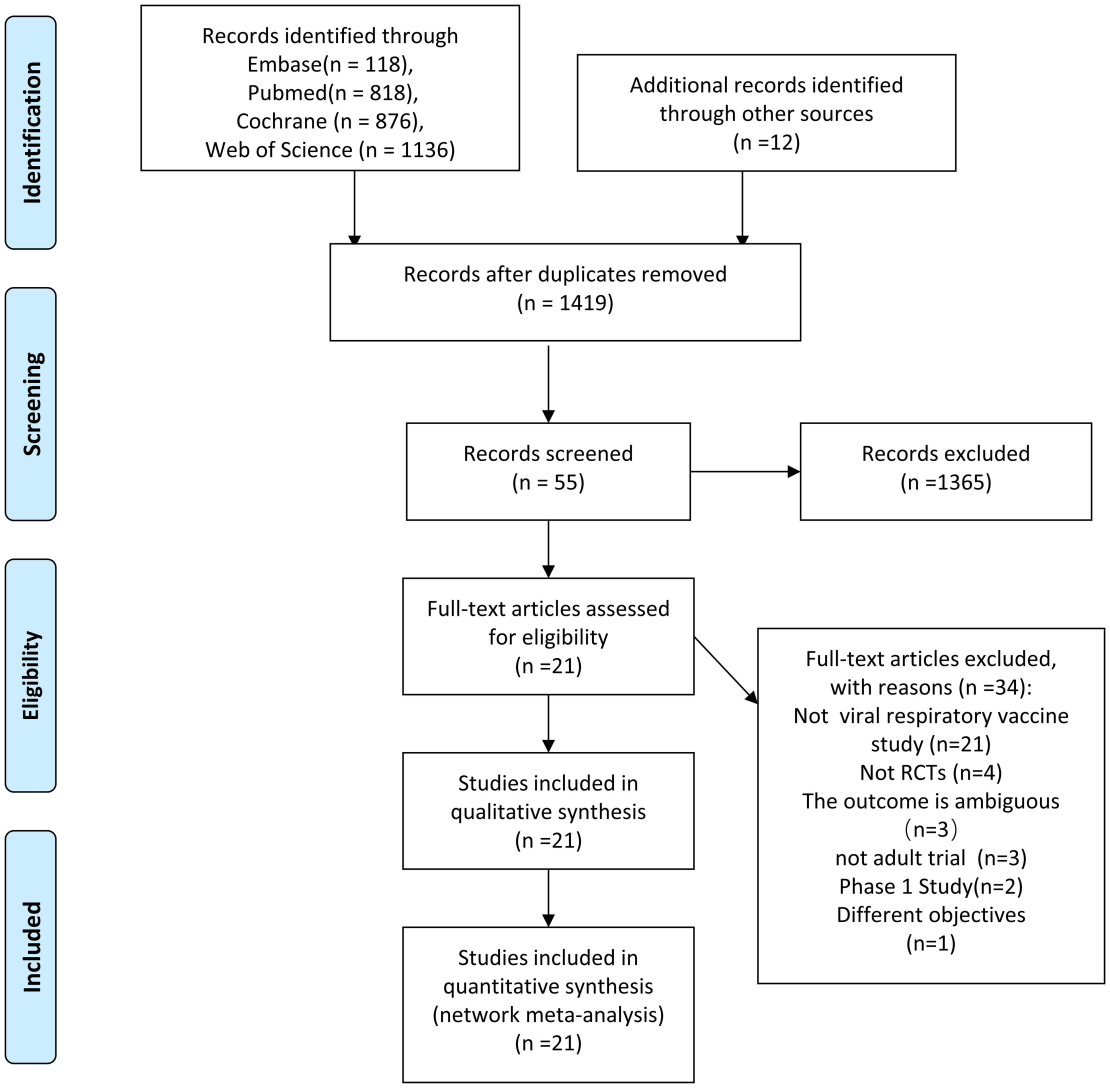
PRISMA Literature search and selection. The study process followed the PRISMA guidelines.

The online database search yielded 2848 results, and an additional 12 were obtained from other sources. A total of 55 articles were considered for full-text assessment after removing the duplicate entries and screening the abstracts (**Supplementary Data Sheet 1 (eTable 3)**), among which only 21 studies comprising 14060 participants were eligible for the analysis (**Figure 1**, total references in **Supplementary Data Sheet 1 (eTable 3)**). The participants received combination or sequential vaccines with VRIDVs. Lazarus et al. included 6 subgroups (6), while Toback et al. included 2 subgroups (17) to evaluate the efficacy of SIVs and SARS-CoV-2 vaccines. Therefore, 27 groups for SIVs and 10 groups for SARS-CoV-2 vaccines were included in the meta-analysis. The main characteristics of the included studies and statistical details of the included groups are presented (**Tables 1**, **2**; **Supplementary Data Sheet 1 (eTables 8–12)**).

**Table 1:** The main characteristics of the included studies.

Author, year/ Main country	group	Interventions	Population	Race (%)	Age, mean (SD), median(range)	Sex, female rate (%)	placebo	sequence	test time	loss rate No. (%)
Schwarz et al., 2011 (18)/ Germany The Netherlands Belgium Hungary	Co-group	PCV13 + TIV/ Placebo	580	white (98.9%)	72 (5.5)	50.35	yes	First	1 month	64 (5.52)
	Seq-group	Placebo + TIV/ PCV13	580	white (98.9%)	72 (5.4)	50.1				
Levin et al., 2018 (8)/ United States	Co-group	ZV + IIV/ Placebo	441	white (83.9%)/ Black (13.9%)	60.8 (7.2)	60.5	yes	First	4 weeks	7 (0.79)
	Seq-group	Placebo + TIV/ ZV	441	white (86.4%)/ Black (11.5%)	61.3 (7.7)	59.3				
Anyinam et al., 2017 (19)/ France Belgium	Co-group	PPV23 + IIV/ Placebo	177	white (98.9%)	68.1 (9.0)	42.9	yes	First	4 weeks	23 (6.46)
	Seq-group	Placebo + IIV/ PPV23	179	white (99.4%)	68.4 (9.4)	43				
Sadoff, et al., 2021 (20)/ United States	Co-group	Ad26.RSV.preF+ Fluarix/ Placebo	90	white (87.8%)/ Black (12.2%)	64.0 (60–82)	63.3	yes	First	4 weeks	6 (3.33)
	Seq-group	Fluarix+Placebo/ Ad26.RSV.preF	90	white (90%)/ Black (8.9%)	66.0 (60–81)	62.2				
Kerzner et al.,, 2007 (21)/ United States	Co-group	VZV+TIV/ Placebo	382	white (67.5%)/ Black (30.6%)	63.4 (7.99)	56.3	yes	First	4 weeks	27 (3.54)
	Seq-group	TIV+placebo/ VZV	380	white (68.7%)/ black (29.2%)	63.6 (8.24)	55.8				
Severance et al., 2022 (22)/ United States	Co-group	V114 + QIV/ Placebo	600	white (82.3%)/ black(12.2%)/ Asian(4.2%)	64.2 (50−98)	55.1	yes	First	1 month	34 (2.83)
	Seq-group	QIV + Placebo/ V114	600	white (82.8%)/ black(10.5%)/ Asian(5%)	64.2 (50−88)	57.2				
Nakashima et al., 2018 (23)/ Japan	Co-group	PPSV23 + QIV	81	Asian	71.0 (5.1)	40.7	no	First	4-6 weeks	5 (3.08)
	Seq-group	QIV/ PPSV23	81	Asian	71.2 (4.1)	38.8				
Thompson et al., 2019 (24)/ United States	Co-group	PCV13 + QIV/ Placebo	441	white (89.8%)/ black(6.9%)	67 (9.1)	53.7	yes	First	1 month	36 (4.08)
	Seq-group	Placebo + QIV/ PCV13	441	white (88.9%)/ black(7.8%)	66.4 (8.8)	56.7				
Toback et al., 2022 (17)/ UK	young- Co-group	NVX-CoV2373+ Quadrivalent	217	white (75.1%)/ Multiple(12.2%)/ Asian(6.5%)	43.2 (14.1)	43.3	yes	First	3 weeks	0
	aged- Co-group	placebo+ Quadrivalent								
	young- Seq-group	NVX-CoV2373+ Trivalent	214	white (76.6%)/ Multiple(10.7%)/ Asian(10.7%)	41.9 (13.2)	44.9				0
	aged- Seq-group	placebo+ Trivalent								
	Covid alone	NVX-CoV2373 alone	502	white (87.1%)/ Multiple(0.6%)/ Asian(7.8%)	51.6 (15.7)	48.6				0
	placebo	placebo	497	white (87.7%)/ Multiple(0.4%)/ Asian(6.8%)	51.4 (15.4)	41.6				0
Lazarus et al., 2021 (6)/ UK	ChA+CQV- Co-group	ChA+CQV/ placebo	65	white (89%)	52 (40–57)	66	yes	second	3 weeks	1 (1.54)
	ChA+CQV- Seq-group	ChA+placebo/ CQV	64	white (96%)	54 (43–61)	59				0
	BNT+CQV- Co-group	BNT+CQV/ placebo	68	white (92%)	48 (35–60)	75				0
	BNT+CQV- Seq-group	BNT+placebo/ CQV	71	white (98%)	47 (34–58)	68				3 (4.22)
	ChA+TV- Co-group	ChA+TV/ placebo	73	white (98%)	69 (67–72)	60				2 (2.73)
	ChA+TV- Seq-group	ChA+placebo/ TV	73	white (98%)	71 (69–72)	42				2 (2.74)
	BNT+TV- Co-group	BNT+TV/ placebo	41	white (100%)	68 (67–70)	59				0
	BNT+TV- Seq-group	BNT+placebo/ TV	38	white (100%)	68 (67–70)	37				0
	ChA+RQV- Co-group	ChA+RQV/ placebo	64	white (94%)	56 (51–60)	53				1 (1.56)
	ChA+RQV- Seq-group	ChA+placebo/ RQV	64	white (100%)	52 (44–60)	58				1 (1.56)
	BNT+RQV- Co-group	BNT+RQV/ placebo	29	white (95%)	42 (31–53)	62				1 (3.44)
	BNT+RQV- Seq-group	BNT+placebo/ RQV	29	white (96%)	39 (33–47)	52				0
Song et al., 2017 (9)/ Korea	Co-group	MF59-aTIV + PCV13	391	Asian	65.4 (0.5)	65.7	no	First	4 weeks	18 (4.6)
	Seq-group	PCV13 alone	413	Asian	65.2 (0.5)	70.6				19 (4.6)
	Seq-group	MF59-aTIV alone	390	Asian	65.9 (0.5)	70.4				8 (2.05)
Zimmermann et al., 2013 (25)/ France and Germany	Co-group	Tdap-IPV+IV	478	unkonw	68.8 (6.2)	54	no	First	4-5 weeks	2 (0.42)
	Seq-group	IV/Tdap-IPV	476	unkonw	68.8 (6.5)	58.6				16 (1.26)
Izikson et al., 2022 (10)/ United States	Co-group	mRNA-1273+ QIV-HD	100	white (94%)	71.0 (67.5–74.0)	54	no	First	3 weeks	4 (4)
	Seq-group	QIV-HD alone	101	white (93%)	71·0 (68.0–74.5)	53				9 (8.91)
	Seq-group	mRNA-1273 alone	105	white (98%)	72.0 (69.0–74.0)	61				1 (0.95)
Schwarz et al., 2017 (26)/ Canada Germany United States	Co-group	HZ/su+ IIV4/ HZ/su	413	white (92.3%)/ black(2.2%)/ Asian(4.1%)	65.9 (8.3)	51.1	no	First	3 weeks	29 (7.02)
	Seq-group	IIV4/ HZ/su/ HZ/su	415	white (91.8%)/ black (1.2%)/ Asian (4.8%)	63.4 (8.8)	52.5				21 (5.06)
Song et al., 2015 (27)/ Korea	Co-group	MF59-aIIV3 + PPSV23 in contralateral arms	56	Asian	71.2 (4.6)	69.2	no	First	4 weeks	4 (7.14)
	Co-group	MF59-aIIV3 + PPSV23 in same arms	56	Asian	71.0 (4.1)	69.1				1 (1.78)
	Seq-group	MF59-aIIV3 alone	56	Asian	71.0 (4.2)	58.9				0
	Seq-group	PPSV23 alone	56	Asian	71.9 (4.5)	64.2				3 (5.36)
Ortiz et al., 2022 (28)/ United States	Co-group	H7N9IIV+IIV4/ H7N9IIV	62	white (35.5%)/ black (58.1%)/ Asian (4.8%)	39.1 (13.1)	41.9	no	First	3 weeks	4 (6.45)
	Seq-group	IIV4/ H7N9IIV /H7N9IIV	53	white (32.1%)/ black (54.7%)/ Asian (5.7%)	38.1 (11.5)	49.1				3 (6)
	Seq-group	IIV4	34	white (41.2%)/ black (50%)/ Asian (2.9%)	35.7 (10.6)	38.2				4 (11.76)
Weston et al., 2012 (29)/ United States	Co-group	Tdap + Flu	112	white (96.4%)	71.3 (5.1)	49.1	no	First	1 month	2 (1.79)
	Seq-group	Flu/Tdap	109	white (96.3%)	71.9 (5.8)	43.1				4 (3.67)
Weston et al., 2009 (30)/ United States	Co-group	Tdap + Flu	748	white (87%)/ black (9.1%)	45.8 (12.6)	63.3	no	First	1 month	19 (2.54)
	Seq-group	Flu/ Tdap	749	white (85.9%)/ black(10.3%)	46.4 (12.1)	57.6				89 (11.88)
Wang et al., 2022 (31)/ China	Co-group	C1:CoronaVac+ QIV/ CoronaVac	120	Asian	43.7 (9.6)	66.6	no		4 weeks	4 (3.33)
	Co-group	C2:CoronaVac/ CoronaVac+ QIV	120	Asian	46.1 (8.6)	60.8				4 (3.33)
	Seq-group	S:CoronaVac/ QIV/ CoronaVac	240	Asian	44.8 (9.2)	63.7				12 (5)
Frenck et al., 2012 (32)/ United States	Co-group	PCV13 + TIV/ Placebo	554	white (91.1%)/ black(6.2%)	54.6 (2.8)	57.9	yes	First	4 weeks	25 (4.51)
	Seq-group	Placebo + TIV/ PCV13	562	white (91.3%)/ black(7.5%)	54.6 (2.9)	57.5				34 (6.05)
Herbinger et al., 2013 (33)/ Germany	Seq-group	TIV+placebo/ H5N1 IV	199	white (97%)	28.3 (8.0)	64	yes	First	3 weeks	5 (2.51)
	Seq-group	H5N1 IV+ placebo/ TIV	203	white (98%)	30.6 (9.8)	64				1 (0.49)
	Co-group	TIV+ H5N1 IV/ H5N1 IV	199	white (97%)	31.0 (9)	58				1 (0.5)

1. In the Toback et al., 2022 study, the placebo group was not included.

**Table 2:** The main outcomes of the included studies.

Author year	outcome for influenza vaccine	outcome for covid-19 vaccine
**Schwarz et al., 2011** (18)	**GMT**: HAI geometric mean titres **SPR**: hemagglutination inhibition assay titer ≥40 **GMFR**: geometric mean fold rise **Adverse events**: fever, fatigue, headache, chills, erythra, gastrointestinal., arthralgia, muscle pain, local pain, erythema, itch, swelling	
**Levin et al., 2018** (8)	**SCR**: a 4-fold rise-in subjects who are seropositive (HAI titer ≥1:10) or a titer of ≥1:40 postvaccination in subjects who are seronegative (HAI titer < 1:10) **SPR**: subjects with titers ≥1:40/ **GMT**: Geometric Mean Titers **GMFR**: geometric mean fold rise **Adverse events**: fever, chills, muscle pain, local pain, erythema, itch, swelling	
**Anyinam et al., 2017** (19)	**SCR**: a 4-fold rise in subjects who are seropositive (HAI titer ≥1:10) or a titer of ≥1:40 postvaccination in subjects who are seronegative (HAI titer < 1:10) **SPR**: serum HI titer greater than or equal to (≥) 1:40 **GMFR**: fold increase in serum haemagglutination inhibition (HI) GMTs post-vaccination compared to pre-vaccination **Adverse events**: fever, fatigue, headache, chills, gastrointestinal., arthralgia, local pain, erythema, itch, swelling, fatigue-G3, headache-G3, chills-G3, gastrointestinal -G3, arthralgia-G3, muscle pain-G3, pain-G3, erythema-G3, swelling-G3	
**Sadoff et al., 2021** (20)	**GMT**: Geometric Mean Titers **SCR**: a 4-fold rise in subjects who are seropositive (HAI titer ≥1:10) or a titer of ≥1:40 postvaccination in subjects who are seronegative (HAI titer < 1:10) **SPR**: subjects with titers ≥1:40 **Adverse events**: fever, fatigue, headache, chills, gastrointestinal., arthralgia, muscle pain, pain, erythema, itch, swelling, pain-G3, swelling-G3	
**Kerzner et al., 2007** (21)	**GMT**: Geometric Mean Titers **SPR**: antibody titer of 1:40 or greater at Week 4 **SCR**: a 4-fold rise in subjects who are seropositive (HAI titer ≥1:10) or a titer of ≥1:40 postvaccination in subjects who are seronegative (HAI titer < 1:10) **Adverse events**: fever, headache, chills, arthralgia, pain, erythema, itch, swelling	
**Severance et al., 2022** (22)	**GMT**: Geometric Mean Titers **SCR**: a 4-fold rise in subjects who are seropositive (HAI titer ≥1:10) or a titer of ≥1:40 postvaccination in subjects who are seronegative (HAI titer < 1:10) **SPR**: subjects with titers ≥1:40 **GMFR**: geometric mean fold rise **Adverse events**: fever, fatigue, headache, chills, arthralgia, muscle pain, local pain, erythema, itch, swelling,	
**Nakashima et al., 2018** (23)	**SPR**: post-vaccination titer ≥1:40 **Adverse events**: fever, fatigue, headache, chills, erythra, local pain, erythema, itch, swelling	
**Thompson et al., 2019** (24)	**GMT**: Geometric Mean Titers **SCR**: a 4-fold rise in subjects who are seropositive (HAI titer ≥1:10) or a titer of ≥1:40 postvaccination in subjects who are seronegative (HAI titer < 1:10) **GMFR**: geometric mean fold rise **SPR**: subjects with titers ≥1:40 **Adverse events**: fever, fatigue, headache, chills, erythra, gastrointestinal., arthralgia, muscle pain, pain, erythema, itch, swelling	
**Toback et al., 2022** (17)	**GMT**: Geometric Mean Titers **SCR**: a 4-fold rise in subjects who are seropositive (HAI titer ≥1:10) or a titer of ≥1:40 postvaccination in subjects who are seronegative (HAI titer < 1:10) **GMFR**: geometric mean fold rise **Adverse events**: fever, fatigue, headache, chills, gastrointestinal., arthralgia, muscle pain, malaise, local pain, erythema, itch, swelling, fatigue-G3, headache-G3, gastrointestinal -G3, arthralgia-G3, muscle pain-G3, malaise-G3, pain-G3, erythema-G3, swelling-G3	**GMEU**: Geometric mean ELISA unit **SCR**: Seroconversion rate **GMFR**: Geometric mean fold rise **Adverse events**: fever, fatigue, headache, malaise, muscle pain, gastrointestinal., local pain, fever-G3, fatigue-G3, headache-G3, malaise-G3, muscle pain-G3, gastrointestinal-G3, Chills-G3,
**Lazarus et al., 2021** (6)	**GMT**: Geometric Mean Titers SCR: a 4-fold rise in subjects who are seropositive (HAI titer ≥1:10) or a titer of ≥1:40 postvaccination in subjects who are seronegative (HAI titer < 1:10) **Adverse events**: fever, fatigue, headache, chills, gastrointestinal., arthralgia, muscle pain, malaise, local pain, erythema, itch, swelling, fatigue-G3, headache-G3, gastrointestinal -G3, arthralgia-G3, muscle pain-G3, malaise-G3, pain-G3,	**GMT**: Geometric Mean Titers **SCR**: a 4-fold rise in subjects who are seropositive (HAI titer ≥1:10) or a titer of ≥1:40 postvaccination in subjects who are seronegative (HAI titer < 1:10) **Adverse events**: fever, fatigue, headache, malaise, muscle pain, gastrointestinal., Chills, local pain, fever-G3, fatigue-G3, headache-G3, malaise-G3, muscle pain-G3, gastrointestinal-G3, Chills-G3,
**Song et al., 2017** (9)	**GMT**: Geometric Mean Titers **SCR**: a 4-fold rise in subjects who are seropositive (HAI titer ≥1:10) or a titer of ≥1:40 postvaccination in subjects who are seronegative (HAI titer < 1:10) **SPR**: subjects with titers ≥1:40 **GMFR**: geometric mean fold rise **Adverse events**: fever, fatigue, headache, chills, arthralgia, muscle pain, local pain, erythema, swelling	
**Zimmermann et al., 2013** (25)	**GMT**: geometric mean anti-haemagglutinin antibody titres **Adverse events**: not reported	
**Izikson et al., 2022** (10)	**GMT**: geometric mean anti-haemagglutinin antibody titres **GMFR**: geometric mean fold rise **SCR**: a 4-fold rise in subjects who are seropositive (HAI titer ≥1:10) or a titer of ≥1:40 postvaccination in subjects who are seronegative (HAI titer < 1:10) **Adverse events**: fever, fatigue, headache, chills, gastrointestinal., arthralgia, muscle pain, malaise, pain, erythema, swelling, fatigue-G3, headache-G3, chills-G3, gastrointestinal -G3, arthralgia-G3,muscle pain-G3, malaise-G3, pain-G3, erythema-G3, swelling-G3	**GMC**: geometric mean concentration **SCR**: a ≥2-times or ≥4-times rise in concentration **GMFR**: geometric mean concentration fold rise **Adverse events**: fever, fatigue, headache, malaise, muscle pain, gastrointestinal., Chills, local pain, fever-G3,fatigue-G3,headache-G3,malaise-G3,muscle pain-G3, gastrointestinal-G3,Chills-G3,
**Schwarz et al., 2017** (18)	**GMT**: Geometric Mean Titers **SCR**: a 4-fold rise in subjects who are seropositive (HAI titer ≥1:10) or a titer of ≥1:40 postvaccination in subjects who are seronegative (HAI titer < 1:10) **SPR**: subjects with titers ≥1:40 **GMFR**: geometric mean fold rise/geometric mean fold rise **Adverse events**: fever, fatigue, headache, chills, gastrointestinal., arthralgia, muscle pain, local pain, erythema, swelling, fatigue-G3, headache-G3, chills-G3, gastrointestinal -G3, pain-G3, erythema-G3, swelling-G3	
**Song et al., 2015** (27)	**GMT**: Geometric Mean Titers **SCR**: a 4-fold rise in subjects who are seropositive (HAI titer ≥1:10) or a titer of ≥1:40 postvaccination in subjects who are seronegative (HAI titer < 1:10) **SPR**: subjects with titers ≥1:40 **GMFR**: geometric mean fold rise **Adverse events**: fever, fatigue, headache, chills, arthralgia, muscle pain, malaise, pain, erythema, swelling,	
**Ortiz et al., 2022** (28)	**GMT**: Geometric Mean Titers **SCR**: a 4-fold rise in subjects who are seropositive (HAI titer ≥1:10) or a titer of ≥1:40 postvaccination in subjects who are seronegative (HAI titer < 1:10) **SPR**: subjects with titers ≥1:40 **Adverse events**: fever, fatigue, headache, gastrointestinal., arthralgia, muscle pain, malaise, pain, erythema, itch, swelling	
**Weston et al., 2012** (29)	**GMT**: Geometric Mean Titers **SCR**: a 4-fold rise in subjects who are seropositive (HAI titer ≥1:10) or a titer of ≥1:40 postvaccination in subjects who are seronegative (HAI titer < 1:10) **SPR**: subjects with titers ≥1:40 **Adverse events**: fever, fatigue, headache, chills, gastrointestinal., arthralgia, muscle pain, local pain, erythema, swelling, fatigue-G3, headache-G3, chills-G3, gastrointestinal-G3, arthralgia-G3, muscle pain-G3, pain-G3, erythema-G3, swelling-G3	
**Weston et al., 2009** (30)	**GMT**: Geometric Mean Titers **SCR**: a 4-fold rise in subjects who are seropositive (HAI titer ≥1:10) or a titer of ≥1:40 postvaccination in subjects who are seronegative (HAI titer < 1:10) **SPR**: subjects with titers ≥1:40 **Adverse events**: fever, fatigue, headache, chills, gastrointestinal., arthralgia, muscle pain, local pain, erythema, swelling, fatigue-G3,headache-G3,chills-G3, gastrointestinal -G3, arthralgia-G3,muscle pain-G3,pain-G3,erythema-G3,swelling-G3	
**Wang et al., 2022** (31)	**GMT**: Geometric Mean Titers **SCR**: a 4-fold rise in subjects who are seropositive (HAI titer ≥1:10) or a titer of ≥1:40 postvaccination in subjects who are seronegative (HAI titer < 1:10) **SPR**: subjects with titers ≥1:40 **GMFR**: geometric mean fold rise **Adverse events**: fever, fatigue, headache, erythra, gastrointestinal., muscle pain, local pain, erythema, itch, swelling,	**GMC**: geometric mean concentration SCR: a ≥2-times or ≥4-times rise in concentration **GMFR**: geometric mean concentration fold rise **Adverse events**: fever, fatigue, headache, muscle pain, gastrointestinal., pain, fever-G3, gastrointestinal-G3
**Herbinger et al., 2014** (33)	**GMT**: geometric mean titer **GMFR**: geometric mean fold rise **Adverse events**: fever, fatigue, headache, chills, erythra, gastrointestinal., arthralgia, muscle pain, local pain, erythema, swelling,	
**Frenck et al., 2012** (32)	**GMT**: Geometric Mean Titers **SCR**: a 4-fold rise in subjects who are seropositive (HAI titer ≥1:10) or a titer of ≥1:40 postvaccination in subjects who are seronegative (HAI titer < 1:10) **SPR**: subjects with titers ≥1:40 **Adverse events**: fever, fatigue, headache, chills, gastrointestinal., arthralgia, muscle pain, local pain, erythema, swelling (excluded by sensitivity analysis)	

PCV13, 13-valent pneumococcal conjugate vaccine; TIV, trivalent inactivated influenza vaccine; ZV, zoster vaccine; IIV4, quadrivalent inactivated influenza vaccine; PPV23, 23-valent pneumococcal polysaccharide vaccine; Ad26.RSV.preF, an adenovirus serotype 26 (Ad26) vector encoding Respiratory syncytial virus F protein stabilized in its prefusion conformation (pre-F); Fluarix, Fluarix Quadrivalent inactivated influenza vaccine; VZV, varicella-zoster virus; V114, 15-valent pneumococcal conjugate vaccine; QIV, quadrivalent inactivated influenza vaccine; PPSV23, 23-valent pneumococcal polysaccharide vaccine; NVX-CoV2373, a recombinant vaccine of COVID-19 vaccine; Quadrivalent, quadrivalent influenza cell-based vaccine (Flucelvax); Trivalent, adjuvanted trivalent influenza vaccine (Fluad); ChA, ChAdOx1 COVID-19 vaccine; BNT, BNT162b2 COVID-19 vaccine; CQV, cellular quadrivalent vaccine; TV, MF59C adjuvanted; trivalent vaccine; RQV, recombinant quadrivalent vaccine; MF59-aTIV, MF59-adjuvanted trivalent inactivated influenza vaccine; Tdap-IPV, diphtheria; tetanus; acellular pertussis and inactivated poliomyelitis vaccine; IIV, inactivated influenza vaccine; mRNA-1273, mRNA-1273 SARS-CoV-2 vaccine; QIV-HD, high-dose quadrivalent influenza vaccine; HZ/su, adjuvanted herpes zoster subunit (HZ/su) vaccine; MF59-aIIV3, MF59-adjuvanted trivalent inactivated influenza vaccine; H7N9IIV, AS03-adjuvanted 2017 inactivated influenza A/H7N9 vaccine; Tdap, tetanus toxoid; reduced diphtheria toxoid; and acellular pertussis vaccine; Flu, seasonal influenza vaccine; CoronaVac, SARS-CoV-2 inactivated vaccine; H5N1 IV, MF59-adjuvanted A/H5N1 vaccine.

1. In the Herbinger et al. and Ortiz et al. studies, the vaccines concomitant administered with the seasonal influenza vaccine were the H7N9 and H5N1 influenza vaccines. In Group B of the Herbinger et al. study, increasing titers for the three serotype outcomes of the seasonal influenza vaccine were found in 22 days after H5N1 vaccination alone, with GMRs of 1.4 to 1.6, indicating that H5N1 vaccination affects the outcome of seasonal influenza, we decided to include only Groups A and C in the meta-analysis, and the time of immunogenicity evaluation was chosen 21 days after the second dose of sequential (42 days) to eliminate the interference from H5N1 vaccine.

2. In the Ortiz et al. study, although no H7N9 vaccine in vaccination alone in first round to provided direct evidence that H7N9 affected seasonal influenza. However, we found an increase in H7N9 immunological outcome after the first round of seasonal influenza vaccination, with a GMR of approximately between 1.2 to 2, which indicated a possible effect between IIV4 and H7N9 vaccine. The outcome of seasonal influenza vaccine in the concomitant administered group may be higher than the actual effect.

3. In the Wang et al. study, the protocols of the combined subgroups and combined and sequential vaccination groups were different. First, the vaccination interval was 28 days in the concomitant administered group, while the interval was 14 days in the sequential group, and the difference in vaccination intervals may cause bias. Second, the results of groups C2 and S may have been affected due to possible interference between the SARS-COV-2 vaccine and the seasonal influenza vaccine. Therefore, this study did not include outcomes for subgroup analysis, and only the main outcomes were included.

### Efficacy and safety of concomitant vaccination

**Figure 2:**
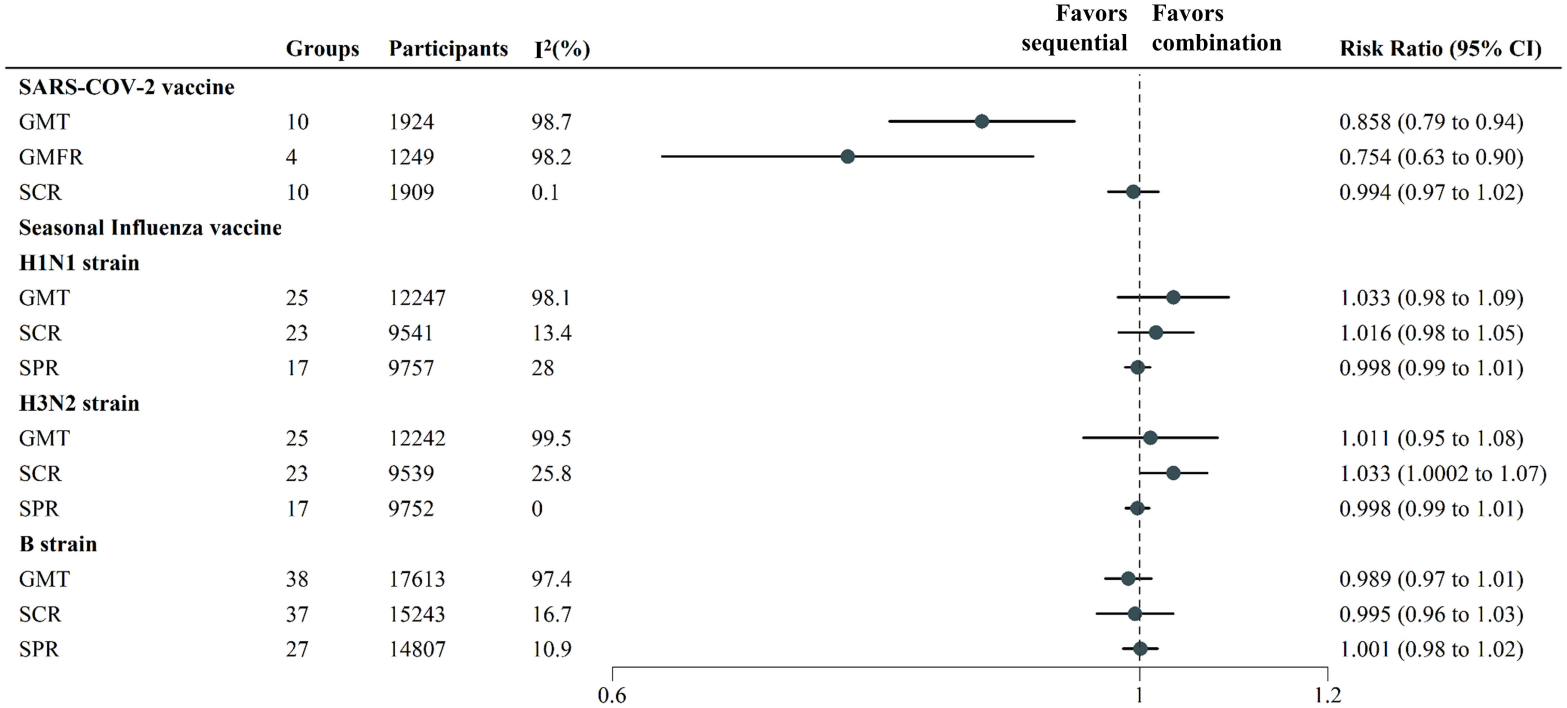
Pooled estimates of concomitant vaccination immunogenicity endpoints of viral respiratory infectious disease vaccines. Each point represents the respective endpoints pooled estimate, derived from Mantel–Haenszel fixed effects model and random effects model based on heterogeneity I^2^. The horizontal lines represent 95% CIs. In analysis of B strain, some studies analyzed 2 strain subtypes, which we combined it. A risk ratio less than 1 favors the sequential or alone vaccination. seroconversion rate (SCR), seroprotection rate (SPR), geometric mean titer (GMT) and geometric mean fold rises (GMFRs).

We report the results of serum-immunogenicity (**Figure 2**; **Supplementary Data Sheet 1 (eFigures 1–12)**). Concomitant administration reduced the GMT (RR: 0.858, 95% CI: (0.785 to 0.939)) and GMFR (0.754 (0.629 to 0.902)) but did not interfere with the SCR (0.994 (0.969 to 1.018)) in the SARS-COV-2 vaccine group. For the SIV group, influenza tests included three strains: H1N1 and H3N2 of A strains, and B strain. Each strain was analyzed for SCR, SPR and GMT. Concomitant immunization increased the SCR by 3.3% (1.033(1.0002 to 1.067) for the H3N2 strains; however, there were no statistically significant differences were found in other results.

**Figure 3:**
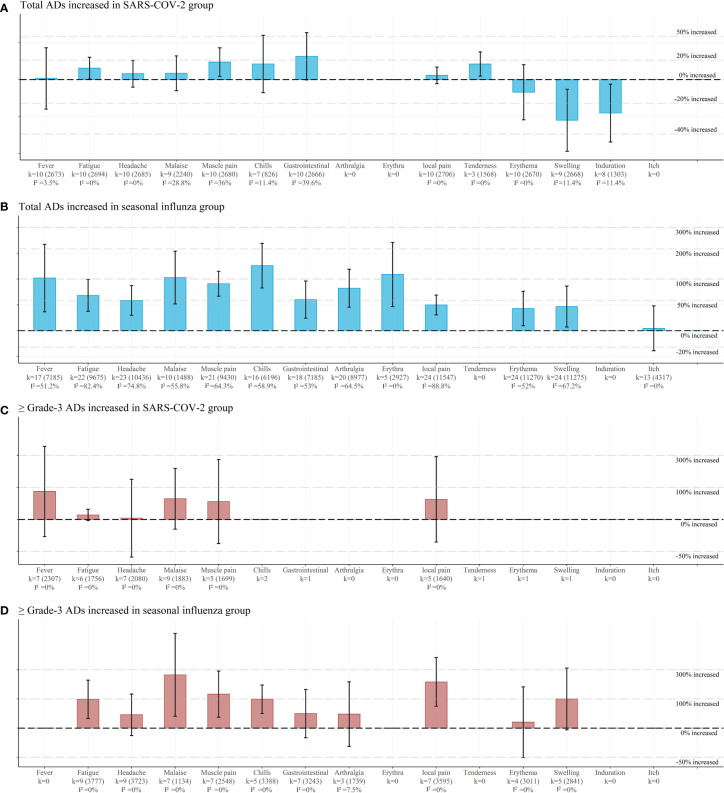
Pooled estimates of concomitant vaccination safety endpoints of viral respiratory infectious disease vaccines. **(A)** Pooled risk ratios (95% CI) for each total adverse events in SARS-COV-2 group. **(B)** Pooled risk ratios (95% CI) for each total adverse events in seasonal influenza group. **(C)** Pooled risk ratios (95% CI) for each ≥Grade-3 adverse events in SARS-COV-2 group. **(D)** Pooled risk ratios (95% CI) for each ≥Grade-3 adverse events in seasonal influenza group. Each point represents the respective endpoints pooled estimate, derived from Mantel–Haenszel fixed effects model and random effects model based on heterogeneity I^2^. The vertical lines represent 95% CIs. Each horizontal dashed line represents the increase or decrease in the incidence of adverse events for concomitant vaccination. k represents the number of studies and participants within each group. Meta-analysis when within-group studies > 3 groups.

In the SARS-COV-2 vaccine group (**Figures 3A, C**; **Supplementary Data Sheet 1 (eFigures 13–30)**), concomitant immunization increased fatigue incidence by 11.3% (1.113 (1.005 to 1.234)) and muscle pain incidence by 17.9% (1.179 (1.031 to 1.348)) for the systemic ADs. and increased 15.8% (1.158(1.033 to 1.297)) tenderness incidence and decreased 27% (0.73 (0.556 to 0.959)) of induration incidence among the local ADs.

In the SIV group (**Figures 3B, D**; **Supplementary Data Sheet 1 (eFigures 31–52)**), concomitant immunization increased several parameters of systemic ADs. These included 102.5% (2.025 (1.287 to 3.184)) of fever incidence; 60.6% (1.606 (1.297 to 1.988)) of total and 98.1% (1.981 (1.255 to 3.127)) of Grade-3 fatigue incidence; 50% (1.5 (1.229 to 1.831)) of headache incidence; 139.4%(2.394 (1.774 to 3.229)) of total and 98.7% (1.987 (1.418 to 2.784)) of Grade-3 chills incidence; 112.7% (2.127 (1.383 to 3.269)) of total rash incidence; 52.7% (1.517 (1.182 to 1.948) of gastrointestinal symptoms incidence; 76.9% (1.769 (1.372 to 2.281)) of arthralgia incidence; 87.9% (1.879 (1.589 to 2.221)) of total and 124.3% (2.243 (1.297; 3.879)) of Grade-3 muscle pain incidence; 104.2% (2.042 (1.433 to 2.909)) of total and 254.5% (3.545 (1.326 to 9.477)) of Grade-3 malaise incidence. Similarly, concomitant vaccination increased the local ADs parameters, including 98.1% (1.981 (1.255 to 3.127)) of total and 199.9% (2.998 (1.68 to 5.25)) of Grade-3 local pain, 34.7% (1.347 (1.069 to 1.696)) of redness, and 38.2% (1.382 (1.049 to 1.819)) of swelling.

### Influencing factors of vaccine type

In the subgroup analysis (**Supplementary Data Sheet 1 (eFigures 54–56)**, **Supplementary Data Sheet 1 (eTables 27–48)**), the GMT (p< 0.001) and GMFR (p<0.001) of the recombinant vaccine were more effective in the immunological group than those of the inactivated and split vaccine in the SARS-COV-2 vaccine group. The concomitant immunization results of the SIV group showed that the GMT (p<0.001; B strain), SCR (p=0.014; B strain), and the SPR (p=0.039; H1N1 strain) of the recombinant vaccine were more effective than those of the inactivated and split vaccines. No statistically significant results were found in the analysis of the ADs except for the local pain in the SIV group. Inactivated and split vaccines increased the ADs more than the recombinant vaccine (p=0.048).

### Influencing factors of concomitant administered vaccine type

The subgroup analysis (**Supplementary Data Sheet 1 (eFigures 57–59)**, **Supplementary Data Sheet 1 (eTables 13–48)**) showed that the split vaccine was more effective than GMFR (p=0.01) in the SARS-COV-2 immunogenicity group. However, no statistically significant results were found in the SIV group. For the ADs analysis of the SARS-COV-2 vaccine group, the muscle pain results showed that inactivated and recombinant vaccines increased the ADs more than the split vaccine (p=0.048). Moreover, concomitant administering SIV with recombinant and mRNA vaccines significantly increased ADs incidences, including fever (p<0.001), fatigue (p<0.001), headache (p<0.001), chills (p<0.001), muscle pain (p=0.027), and gastrointestinal symptoms (p<0.001) for systemic ADs and local pain for local ADs, than with other types of vaccines.

### Influencing factors of gender proportion

In the subgroup analysis (**Supplementary Data Sheet 1 (eTables 13–48)**), the GMFR (p=0.01) of the SARS-COV-2 vaccine and SPR (p=0.045) of the B strain revealed that concomitant vaccination was more efficient in groups with ≥55% females. Notably, there were no statistically significant results in the ADs of the subgroups. According to meta-regression analysis, the GMT (0.21% (0.086 to 0.35%) per 1% of female proportions increased in the SARS-COV-2 vaccine group, consistent with the results of the subgroup analysis. The ADs analysis showed that gastrointestinal symptoms and malaise increased by 5.07% (1.62 to 8.51%) and 3.19% (0.38 to 6.01%), respectively, in the SIV group. However, there were no statistically significant results in the meta-regression analysis of the SARS-COV-2 vaccine ADs and SIV immunogenicity groups.

### Influencing factors of mean age

The subgroup analysis (**Supplementary Data Sheet 1 (eTables 13–48)**) showed that the age distribution was around 18 to 80 years. The Geometric Mean Titers (GMT) and Geometric Mean Fold Rises (GMFR) in the SARS-CoV-2 vaccine immunogenicity group demonstrated a more pronounced effect of concomitant vaccination in participants with a mean age of 65 years and older, with statistical significance (p < 0.001 for both GMT and GMFR). Conversely, in the Seasonal Influenza Vaccine (SIV) immunogenicity group, no significant differences were noted in GMT and GMFR. The ADs increasing rate of concomitant vaccination was higher in the mean age < 65 group, with significantly increased fatigue (p=0.02), malaise (p=0.007) and muscle pain (p=0.02) in the SRAS-COV2 group. In contrast, the SIV group did not exhibit significant differences in these symptoms. According to meta-regression analysis, there was an increase in the GMT (0.81% (0.64 to 0.98%) per year of age and GMFR (1.7% (1.1 to 2.28%)) of the SARS-COV-2 vaccine immunogenicity group. Moreover, increase in fatigue (-0.88% (-1.69 to -0.07%)), malaise (-3.45% (-6.78 to-0.12%)) and local pain (-0.7% (-1.27 to-0.13%)) was observed in the SARS-COV-2 vaccine ADs group, but there were no statistically significant results in the SIV group.

### Influencing factors of placebo used

For the subgroup analysis (**Supplementary Data Sheet 1 (eTables 13–48)**), in the SARS-COV-2 vaccine group, the concomitant immunization efficacy was improved upon the study use placebo, as shown by the GMT of p=0.04. Moreover, muscle pain (p=0.048) was higher in the placebo used group than in the no-placebo group. In the SIV group of the B strain group, the concomitant immunization effect was elevated by 3-4% with placebo use compared to no-placebo use in GMT and SPR; however, no differences were observed in the ADs of the SIV group. In meta-regression, the GMT of the SARS-COV-2 vaccine group increased by 7.57% (2.33 to 12.8%) with placebo used, and that of the seasonal influenza B strain group increased by 6.04% (1.75 to 10.33%). Additionally, the muscle pain of the SARS-COV-2 vaccine ADs group increased by 35.58% (0.18 to 70.97%) with placebo used. No statistically significant results were found in the ADs of the SIV group.

### Influencing factors of adjuvants used

In the subgroup analysis (**Supplementary Data Sheet 1 (eTables 13–48)**), the SCR of the H3N2 (p=0.015) strain and B strain (p=0.006) showed that the adjuvants enhanced the concomitant immunization efficacy. Notably, statistically significant results were found in the ADs parameters, including itching (p=0.04). According to meta-regression, the SCR of H3N2 increased by 16.6% (2.42 to 30.8%) when adjuvant was used, while that of the B strain increased by 29.5% (3.94 to 55.1%) in the immunological group.

### Influencing factors of booster dose used

We grouped participants according to whether the SARS-COV-2 vaccination was a first-time or a booster dose (**Supplementary Data Sheet 1 (eTables 13–48)**). We excluded the study by Wang et al. group, due to its design. Participants who received the first-time vaccination had a lower concomitant immunization efficiency (GMT; p<0.001 and GMFR; p<0.001) than those who received the booster dose. The increase in ADs incidences was greater in first-time vaccinated participants (fever; p < 0.001 and local pain; p < 0.001) than in those who received the booster. No statistically significant results were found in the meta-regression analysis of the groups.

### Publication bias assessment and sensitivity analysis

Publication bias was found only in the chills symptoms of the SIV group (p=0.047). In the sensitivity analysis of the ADs, we found that the Herbinger et al. group had a greater impact on the results and heterogeneity of the meta-analysis and thus was excluded from the final meta-analysis.

### Quality assessment

Overall, in combination with previous studies risk of bias assessments, these trials were considered low-moderate risk for bias (**Supplementary Data Sheet 1 (eFigure 53)**). In the GRADE system (**Supplementary Data Sheet 1 (eTables 4–7)**), there was low or very low grading for the SIV group and high grading for the SARS-COV-2 group in the ADs analyses. Similarly, low or very low grading was observed for GMT and GMFR results, while SCR and SPR had high or moderate grading in immunological efficacy.

## Discussion

In this systematic review and meta-analysis, we evaluated the efficacy and safety of VRIDVs when concomitant administered with other vaccines. Four RCTs of the SARS-COV-2 vaccine and 21 RCTs of SIV were included in the meta-analysis. Concomitant immunization reduced the immunogenicity of GMT and GMFR in the SARS-COV-2 vaccine group by 14.2% and 24.6%, respectively. The SCR demonstrated the ability to protect the population (6, 10), both vaccine results showed that concomitant administration protected the population at the same level as sequential vaccination. Clinical studies and meta-analyses have shown that vaccine-induced production of high sero-neutralizing antibody titers declines after 3 ~ 6 months, with a progressive increase in the possibility of breakthrough infection as the titers decline and mutant strains develop (2, 3, 34, 35). Thus, serum immunoprotection declines with time, causing vaccine protection to be maintained only in higher GMT populations, suggesting that the duration of protection from concomitant vaccination is reduced.

In the analysis of the vaccine types, the concomitant administration effect of the recombinant vaccines was better than that of inactivated and split vaccines in some of the SIV group. The concomitant immunization effect of mRNA vaccines was also better than inactivated vaccines in the SARS-COV-2 vaccine group. “Heterologous effects of vaccination” can cross-reactive and bystander activate the classical adaptive immune response and improve the magnitude and durability of humoral and cellular immunity (7, 36–38). Moreover, the components of mRNA and recombinant vaccines may increase immunogenicity (3, 39), and this can potentially improve the duration and efficacy of future vaccines. In the analysis of ADs, concomitant administering SIV with the mRNA and recombinant vaccines increased the incidences of almost all systemic ADs (fever, fatigue, headache, chills, gastrointestinal symptoms, arthralgia, and muscle pain) more than the other vaccine types. In general, concomitant immunization mainly affected the systemic ADs and had less effect on the local ADs. Moreover, inactivated, and split vaccines had better safety than mRNA and recombinant vaccines when concomitant vaccination. This result is like vaccination alone.

In this review, the concomitant vaccination group showed a reduction in the GMT and GMFR among the SARS-COV-2 vaccine participants. However, no significant reduction was observed in the SIV group. Therefore, hypothesized that concomitant administration has greater effectiveness when supplemented with booster vaccination, as also reported by Lazarus et al. and Toback et al. Since most adults have a previous illness-immunity or prior vaccination against seasonal influenza because of the high prevalence of seasonal influenza, the SIV administration could be considered a booster vaccination. As such, the serum immune background of participants against influenza exhibited some reaction. In contrast, the pre-vaccination population has a very low baseline against SARS-COV-2 since covid-19 is an emerging infectious disease. According to the subgroup and meta-regression analysis of the booster vaccine in the SARS-COV-2 group, the immunogenicity of concomitant administration was lower in participants receiving the vaccine for the first time. Moreover, the immunogenicity of concomitant administration versus sequential/alone vaccination was similar to that of the booster vaccination in the SIV group, further supporting our hypothesis. The results affirm that alone vaccination is recommended as a first-time vaccination against SARS-COV-2, new emerging viruses, or other low-intensity VRIDVs, while a combination with other vaccines is a suitable option as a booster vaccination or vaccination against high-intensity VRIDVs.

Older adults exhibited a more robust tolerance to the concomitant administration of the SARS-CoV-2 vaccine compared to younger individuals, the ADs of concomitant administration significantly increased fatigue, malaise, and muscle pain among the participants with <65 years in the SARS-COV-2 vaccine group. This may also be caused by the weaker immunity of the elderly, which is not sensitive to both individual and combined vaccinations. In the SIV group, the elderly received the more suitable 3-valent SIV, while the young received 4-valent SIV. This procedure improved the immunogenicity of the elderly when vaccinated sequentially (10, 40). Consequently, we advocate for the development of vaccines offering broad coverage and prolonged protection, specifically designed for the elderly demographic.

Gender-based analysis revealed intriguing findings, one subgroup in the SARS-COV-2 group and another in the SIVs group showed higher concomitant vaccination efficacy in the group with a higher proportion of women. Notably, the meta-regression analysis showed that this observation was found only in the SARS-COV-2 vaccine group. In addition, a higher proportion of females had increased reports of gastrointestinal symptoms and malaise upon co-vaccination. Some studies reported that females have greater immune responses to vaccines and ADs than males (41, 42), potentially due to heightened sensitivity to the immune stimulation caused by ADs and the multiple antigens present in concomitant vaccination.

In the analysis of the placebo used, the concomitant immunization effect was significantly elevated in the group with placebo in the GMT of B strain and SARS-COV-2 vaccine group compared to the group without placebo. The use of a placebo increased the ADs in the muscle pain group of the SARS-COV-2 vaccine group to the point that participants were confused about their grouping after the placebo administration. People may believe that too many vaccines/antigens overload the immune system, or increase ADs, resulting in less efficacy than when the same vaccine is administered alone (43). This means that concomitant vaccination participants may have a reduced psychological burden, while those in the sequential group may have an elevated psychological burden due to the placebo, thus affecting the efficiency and safety of the administered vaccine. Therefore, educating the participants about the safety and efficacy of concomitant administration is beneficial for reducing psychological burden and improving vaccine efficacy and coverage (44).

The use of adjuvants significantly increased the immunogenicity of concomitant vaccination, with the concomitant administered vaccines being more effective in the adjuvant-containing group. The meta-regression analysis also supported these results, suggesting that adjuvants improve the immunological responses to a certain extent (17-30%). Consequently, this evidence suggests that adjuvant-containing vaccines may yield superior efficacy in concomitant administration scenarios (4).

This study has several strengths that highlight its significance. Of note, this is the first study to evaluate the efficacy and safety of the concomitant administration of SARS-COV-2 vaccines or SIVs with other vaccine in adults. Moreover, we meticulously performed meta-analyses, follow-up subgroup analyses, and meta-regressions of the primary immune and adverse outcomes to provide comprehensive and evidence-based medical data to help develop robust public health strategies. Nonetheless, this study was limited by several factors. First, some studies suggest that simple serologic testing of SARS-CoV-2 antibodies may not reflect the complexity and persistence of protective immunity and there is some testing variation in the quantitative detection (39, 45). The immunological efficacy results were only based on the serological immunological outcomes due to a lack of population outcomes, such as population protected rate and severe disease reduction rate. Thus, the study could not comprehensively reflect the protective efficiency of the discussed vaccines. Secondly, the RCTs studies included in this meta-analysis had few participants, making it difficult to statistically analyze severe ADs with very low incidences, such as anaphylaxis and myocarditis (46, 47). Therefore, future studies should analyze the efficacy and safety of concomitant administering vaccines through real-world studies to capture all vaccine ADs. Finally, this analysis focused on the SARS-COV-2 and SIV, failing to encompass other emerging VRIDVs, leading to potentially biased conclusions.

In conclusion, the results of this meta-analysis suggested that the short-term protection and safety of concomitant administered VRIDVs were reliable and did not interfere with the protection of the elderly (65-80 years old). Concomitant vaccination could improve the efficiency and safety of immunity with booster vaccines, appropriate adjuvants, and health promotion and counselling. However, the protection duration of concomitant vaccination against emerging infectious diseases could be greatly impacted by the first vaccination. Nonetheless, there is a need to develop specific vaccines for the elderly that ensure long-term protection.

## Data availability statement

The original contributions presented in the study are included in the article/**Supplementary Material**. Further inquiries can be directed to the corresponding author.

## Author contributions

DL: Data curation, Software, Visualization, Writing – original draft, Conceptualization, Investigation, Methodology, Supervision. YH: Formal analysis, Investigation, Project administration, Validation, Writing – original draft, Writing – review & editing. RX: Data curation, Supervision, Writing – original draft. MQ: Data curation, Formal analysis, Methodology, Validation, Writing – original draft. JS: Formal analysis, Project administration, Validation, Writing – original draft. CZ: Data curation, Formal analysis, Methodology, Writing – original draft. JZ: Funding acquisition, Resources, Visualization, Writing – original draft. FY: Formal analysis, Software, Writing – original draft. ZL: Methodology, Writing – original draft. YW: Validation, Writing – original draft. CFW: Validation, Writing – original draft. CHW: Formal analysis, Funding acquisition, Project administration, Resources, Validation, Writing – review & editing.
