# Supplementary material for: Evaluation of the efficacy, safety and influencing factors of concomitant and sequential administration of viral respiratory infectious disease vaccines: a systematic review and meta-analysis
Source: Front Immunol. 2023 Dec 21;14:1259399. doi: 10.3389/fimmu.2023.1259399 (PMC10764558; doi:10.3389/fimmu.2023.1259399)

eTable1. PRISMA 2020 checklist
eTable2. Search strategy
eTable3. Inclusion articles and exclusion articles and their specific reasons
eTable4-7. Grading of recommendations, assessment, development and evaluations (GRADE) in immunogenicity and adverse event incidence
eTable8-12. Statistics detail for each included articles in immunogenicity and adverse events incidence
eTable13-26. Subgroup analysis and meta-regression results in SARS-COV-2 vaccine group
eTable27-48. Subgroup analysis and meta-regression results in seasonal influenza vaccine group
eFigure 1-3. Immunogenicity of concomitant vaccination vs sequential vaccination for SARS-COV-2 group in the meta-analysis
eFigure 4-12. Immunogenicity of concomitant vaccination vs sequential vaccination for seasonal influenza group in the meta-analysis
eFigure 13-30. Adverse events incidence of concomitant vaccination vs sequential vaccination for SARS-COV-2 group in the meta-analysis
eFigure 31-52. Adverse events incidence of concomitant vaccination vs sequential vaccination for seasonal influenza group in the meta-analysis
eFigure 53. Risk of Bias Assessments
eFigure 54-56. Subgroup analysis of the types of viral respiratory infectious disease vaccines
eFigure 57-59. Subgroup analysis of the types of concomitant administered vaccines

eTable1. PRISMA 2020 checklist

Section and Topic	Item #	Checklist item	Location where item is reported
**TITLE**			
Title	1	Identify the report as a systematic review.	Page1
**ABSTRACT**			
Abstract	2	See the PRISMA 2020 for Abstracts checklist.	Page2
**INTRODUCTION**			
Rationale	3	Describe the rationale for the review in the context of existing knowledge.	Page4-5
Objectives	4	Provide an explicit statement of the objective(s) or question(s) the review addresses.	Page4-5
**METHODS**			
Eligibility criteria	5	Specify the inclusion and exclusion criteria for the review and how studies were grouped for the syntheses.	Page6
Information sources	6	Specify all databases, registers, websites, organisations, reference lists and other sources searched or consulted to identify studies. Specify the date when each source was last searched or consulted.	Page5-6
Search strategy	7	Present the full search strategies for all databases, registers and websites, including any filters and limits used.	eTable2
Selection process	8	Specify the methods used to decide whether a study met the inclusion criteria of the review, including how many reviewers screened each record and each report retrieved, whether they worked independently, and if applicable, details of automation tools used in the process.	Page6
Data collection	9	Specify the methods used to collect data from reports, including how many reviewers collected data from each report, whether they worked independently, any processes for obtaining or confirming data	Page6

Section and Topic	Item #	Checklist item	Location where item is reported
process		from study investigators, and if applicable, details of automation tools used in the process.	
Data items	10a	List and define all outcomes for which data were sought. Specify whether all results that were compatible with each outcome domain in each study were sought (e.g. for all measures, time points, analyses), and if not, the methods used to decide which results to collect.	Page6
	10b	List and define all other variables for which data were sought (e.g. participant and intervention characteristics, funding sources). Describe any assumptions made about any missing or unclear information.	Page6
Study risk of bias assessment	11	Specify the methods used to assess risk of bias in the included studies, including details of the tool(s) used, how many reviewers assessed each study and whether they worked independently, and if applicable, details of automation tools used in the process.	Page6
Effect measures	12	Specify for each outcome the effect measure(s) (e.g. risk ratio, mean difference) used in the synthesis or presentation of results.	Page7-8
Synthesis methods	13a	Describe the processes used to decide which studies were eligible for each synthesis (e.g. tabulating the study intervention characteristics and comparing against the planned groups for each synthesis (item #5)).	Page7-8
	13b	Describe any methods required to prepare the data for presentation or synthesis, such as handling of missing summary statistics, or data conversions.	Page7-8
	13c	Describe any methods used to tabulate or visually display results of individual studies and syntheses.	Page7-8
	13d	Describe any methods used to synthesize results and provide a rationale for the choice(s). If meta-analysis was performed, describe the model(s), method(s) to identify the presence and extent of	Page7-8

Section and Topic	Item #	Checklist item	Location where item is reported
		statistical heterogeneity, and software package(s) used.	
	13e	Describe any methods used to explore possible causes of heterogeneity among study results (e.g. subgroup analysis, meta-regression).	Page7-8
	13f	Describe any sensitivity analyses conducted to assess robustness of the synthesized results.	Page7-8
Reporting bias assessment	14	Describe any methods used to assess risk of bias due to missing results in a synthesis (arising from reporting biases).	Page6
Certainty assessment	15	Describe any methods used to assess certainty (or confidence) in the body of evidence for an outcome.	Page6
**RESULTS**			
Study selection	16a	Describe the results of the search and selection process, from the number of records identified in the search to the number of studies included in the review, ideally using a flow diagram.	Page8-9 Figure1
	16b	Cite studies that might appear to meet the inclusion criteria, but which were excluded, and explain why they were excluded.	eTable3
Study characteristics	17	Cite each included study and present its characteristics.	Page8-9 Table1-2
Risk of bias in studies	18	Present assessments of risk of bias for each included study.	Page14 eFigure53
Results of individual	19	For all outcomes, present, for each study: (a) summary statistics for each group (where appropriate) and (b) an effect estimate and its precision (e.g. confidence/credible interval), ideally using structured	eTable8-12

Section and Topic	Item #	Checklist item	Location where item is reported
studies		tables or plots.	
Results of syntheses	20a	For each synthesis, briefly summarise the characteristics and risk of bias among contributing studies.	Page8-14
	20b	Present results of all statistical syntheses conducted. If meta-analysis was done, present for each the summary estimate and its precision (e.g. confidence/credible interval) and measures of statistical heterogeneity. If comparing groups, describe the direction of the effect.	Page8-14<br>Figure2,3
	20c	Present results of all investigations of possible causes of heterogeneity among study results.	Page8-14<br>eTable13-48
	20d	Present results of all sensitivity analyses conducted to assess the robustness of the synthesized results.	Page14
Reporting biases	21	Present assessments of risk of bias due to missing results (arising from reporting biases) for each synthesis assessed.	Page14<br>eTable4-7
Certainty of evidence	22	Present assessments of certainty (or confidence) in the body of evidence for each outcome assessed.	Page14<br>eTable4-7
**DISCUSSION**			
Discussion	23a	Provide a general interpretation of the results in the context of other evidence.	Page14-17
	23b	Discuss any limitations of the evidence included in the review.	Page14-17
	23c	Discuss any limitations of the review processes used.	Page18
	23d	Discuss implications of the results for practice, policy, and future research.	Page18-19
**OTHER INFORMATION**			

Section and Topic	Item #	Checklist item	Location where item is reported
Registration and protocol	24a	Provide registration information for the review, including register name and registration number, or state that the review was not registered.	Page5
	24b	Indicate where the review protocol can be accessed, or state that a protocol was not prepared.	Page5
	24c	Describe and explain any amendments to information provided at registration or in the protocol.	Page5
Support	25	Describe sources of financial or non-financial support for the review, and the role of the funders or sponsors in the review.	Page20
Competing interests	26	Declare any competing interests of review authors.	Page20
Availability of data, code and other materials	27	Report which of the following are publicly available and where they can be found: template data collection forms; data extracted from included studies; data used for all analyses; analytic code; any other materials used in the review.	Page20

eTable2. Search strategy

Search strategy

#	search
1	(COVID 19 Vaccines or (Vaccines, COVID-19) or SARS Coronavirus 2 Vaccines or COVID19 Virus Vaccines or (Vaccines, COVID19 Virus) or (Virus Vaccines, COVID19) or COVID19 Virus Vaccine or (Vaccine, COVID19 Virus) or (Virus Vaccine, COVID19) or (COVID19 Vaccines) or (Vaccines, COVID19) or COVID19 Vaccine or (Vaccine, COVID19) or (SARS-CoV-2 Vaccines) or SARS CoV 2 Vaccines or (Vaccines, SARS-CoV-2) or SARS-CoV-2 Vaccine or SARS CoV 2 Vaccine or (Vaccine, SARS-CoV-2) or SARS2 Vaccines or (Vaccines, SARS) or SARS2 Vaccine or (Vaccine, SARS2) or Coronavirus Disease 2019 Vaccines or Coronavirus Disease 2019 Vaccine or Coronavirus Disease 2019 Virus Vaccine or Coronavirus Disease 2019 Virus Vaccines or Coronavirus Disease-19 Vaccines or Coronavirus Disease 19 Vaccines or (Vaccines, Coronavirus Disease-19) or (Coronavirus Disease-19 Vaccine) or Coronavirus Disease 19 Vaccine or (Vaccine, Coronavirus Disease-19) or COVID 19 Vaccin or (Vaccine, COVID 19) or 2019-nCoV Vaccine or 2019 nCoV Vaccine or (Vaccine, 2019-nCoV) or 2019 Novel Coronavirus Vaccines or 2019 Novel Coronavirus Vaccine or 2019-nCoV Vaccines or 2019 nCoV Vaccines or (Vaccines, 2019-nCoV) or COVID-19 Vaccine or (Vaccine, COVID-19) or COVID-19 Virus Vaccines or COVID 19 Virus Vaccines or (Vaccines, COVID-19 Virus) or (Virus Vaccines, COVID-19) or COVID-19 Virus Vaccine or COVID 19 Virus Vaccine or (Vaccine, COVID-19 Virus) or (Virus Vaccine, COVID-19)) .mp.
2	(Influenza Vaccine or (Vaccine, Influenza) or Flu Vaccine or (Vaccine, Flu) or Influenza Virus Vaccines or Flu Vaccines or Influenzavirus Vaccine or (Vaccine, Influenzavirus) or Influenza Virus Vaccine or (Vaccine, Influenza Virus) or (Virus Vaccine, Influenza) or Influenzavirus Vaccines or High-Dose Trivalent Influenza Vaccine or High Dose Trivalent Influenza Vaccine or Monovalent Influenza Vaccines or (Influenza Vaccines, Monovalent) or (Vaccines, Monovalent Influenza) or Monovalent Influenza Vaccine or (Influenza Vaccine, Monovalent) or (Vaccine, Monovalent Influenza) or Trivalent Influenza Vaccine or (Influenza Vaccine, Trivalent) or (Vaccine, Trivalent Influenza) or Intranasal Live-Attenuated Influenza Vaccine or Intranasal Live Attenuated Influenza Vaccine or Universal Flu Vaccines or Universal Flu Vaccine or (Flu Vaccine, Universal) or (Vaccine, Universal Flu) or Universal Influenza Vaccine or (Influenza Vaccine, Universal) or (Vaccine, Universal Influenza) or Universal Influenza Vaccines or Trivalent Live Attenuated Influenza Vaccine or LAIV Vaccine or (Vaccine, LAIV) or Quadrivalent Influenza Vaccine or (Influenza Vaccine, Quadrivalent) or (Vaccine, Quadrivalent Influenza)) .mp.
3	((Vaccines, Viral) or Viral Vaccine or (Vaccine, Viral)) .mp.
4	(Concomitant vaccination or Administered concomitantly or Concomitant

	administration or Concomitant or Combined vaccination or Combination vaccination or (Concomitant, Vaccination) or (Administered , Concomitantly) or Concomitant or Combination or Combined).mp.
5	(Adult) .mp.
6	(Randomized Controlled Trial or Clinical Trial) .mp.<br>([controlled clinical trial]/lim OR [randomized controlled trial]/lim)
7	#1 OR #2 OR #3
8	#7 AND #4 AND #5 LIN#6

mp=ti, ab, hw, tn, ot, dm, mf, dv, kw, fx, dq, nm, kf, ox, px, rx, ui, sy, tc, id, tm

searched results

database	time	results	final search date
EMBASE	2	118	Spe 1, 2022
Pubmed	2	818	Spe 1, 2022
Cochrane Central Register of Clinical Trials	2	876	Spe 1, 2022
Web of Science	2	1136	Spe 1, 2022

eTable3. Inclusion articles

	References
**1**	[1] Schwarz TF, Flamaing J, Rümke HC, Penzes J, Juergens C, Wenz A, et al. A randomized, double-blind trial to evaluate immunogenicity and safety of 13-valent pneumococcal conjugate vaccine given concomitantly with trivalent influenza vaccine in adults aged ≥65 years. Vaccine 2011;29:5195-202, https://doi.org/10.1016/j.vaccine.2011.05.031.
**2**	[2] Levin MJ, Buchwald UK, Gardner J, Martin J, Stek JE, Brown E, et al. Immunogenicity and safety of zoster vaccine live administered with quadrivalent influenza virus vaccine. Vaccine 2018;36:179-85, https://doi.org/10.1016/j.vaccine.2017.08.029.
**3**	[3] Ofori-Anyinam O, Leroux-Roels G, Drame M, Aerssens A, Maes C, Amanullah A, et al. Immunogenicity and safety of an inactivated quadrivalent influenza vaccine co-administered with a 23-valent pneumococcal polysaccharide vaccine versus separate administration, in adults ≥50 years of age: results from a phase iii, randomized, non-inferiority trial. Vaccine 2017;35:6321-8, https://doi.org/10.1016/j.vaccine.2017.09.012.
**4**	[4] Sadoff J, De Paepe E, Haazen W, Omoruyi E, Bastian AR, Comeaux C, et al. Safety and immunogenicity of the ad26.rsv.pref investigational vaccine coadministered with an influenza vaccine in older adults. The Journal of Infectious Diseases 2021;223:699-708, https://doi.org/10.1093/infdis/jiaa409.
**5**	[5] Kerzner B, Murray AV, Cheng E, Ifle R, Harvey PR, Tomlinson M, et al. Safety and immunogenicity profile of the concomitant administration of zostavax and inactivated influenza vaccine in adults aged 50 and older. J Am Geriatr Soc 2007;55:1499-507, https://doi.org/10.1111/j.1532-5415.2007.01397.x.
**6**	[6] Severance R, Schwartz H, Dagan R, Connor L, Li J, Pedley A, et al. Safety, tolerability, and immunogenicity of v114, a 15-valent pneumococcal conjugate vaccine, administered concomitantly with influenza vaccine in healthy adults aged ≥50 years: a randomized phase 3 trial (pneu-flu). Hum Vaccin Immunother 2022;18:1-14, https://doi.org/10.1080/21645515.2021.1976581.
**7**	[7] Nakashima K, Aoshima M, Ohfuji S, Yamawaki S, Nemoto M, Hasegawa S, et al. Immunogenicity of simultaneous versus sequential administration of a 23-valent pneumococcal polysaccharide vaccine and a quadrivalent influenza vaccine in older individuals: a randomized, open-label, non-inferiority trial. Hum Vaccin Immunother 2018;14:1923-30, https://doi.org/10.1080/21645515.2018.1455476.

	References
**8**	[8] Thompson AR, Klein NP, Downey HJ, Patterson S, Sundaraiyer V, Watson W, et al. Coadministration of 13-valent pneumococcal conjugate and quadrivalent inactivated influenza vaccines in adults previously immunized with polysaccharide pneumococcal vaccine 23: a randomized clinical trial. Hum Vaccin Immunother 2018;15:444-51, https://doi.org/10.1080/21645515.2018.1533777.
**9**	[9] Toback S, Galiza E, Cosgrove C, Galloway J, Goodman AL, Swift PA, et al. Safety, immunogenicity, and efficacy of a covid-19 vaccine (nvx-cov2373) co-administered with seasonal influenza vaccines: an exploratory substudy of a randomised, observer-blinded, placebo-controlled, phase 3 trial. Lancet Respir Med 2022;10:167-79, https://doi.org/10.1016/S2213-2600(21)00409-4.
**10**	[10] Lazarus R, Baos S, Cappel-Porter H, Carson-Stevens A, Clout M, Culliford L, et al. Safety and immunogenicity of concomitant administration of covid-19 vaccines (chadox1 or bnt162b2) with seasonal influenza vaccines in adults in the uk (comflucov): a multicentre, randomised, controlled, phase 4 trial. Lancet 2021;398:2277-87, https://doi.org/10.1016/S0140-6736(21)02329-1.
**11**	[11] Song JY, Cheong HJ, Hyun HJ, Seo YB, Lee J, Wie S, et al. Immunogenicity and safety of a 13-valent pneumococcal conjugate vaccine and an mf59-adjuvanted influenza vaccine after concomitant vaccination in ⩾60-year-old adults. Vaccine 2017;35:313-20, https://doi.org/10.1016/j.vaccine.2016.11.047.
**12**	[12] Zimmermann U, Gavazzi G, Richard P, Eymin C, Soubeyrand B, Baudin M. Immunogenicity and safety of a booster dose of diphtheria, tetanus, acellular pertussis and inactivated poliomyelitis vaccine (tdap-ipv; Repevax®) administered concomitantly versus non-concomitantly with an influenza vaccine (vaxigrip®) to adults aged ≥60 years: an open-label, randomised trial. Vaccine 2013;31:1496-502, https://doi.org/10.1016/j.vaccine.2012.12.081.
**13**	[13] Izikson R, Brune D, Bolduc JS, Bourron P, Fournier M, Moore TM, et al. Safety and immunogenicity of a high-dose quadrivalent influenza vaccine administered concomitantly with a third dose of the mrna-1273 SARS-cov-2 vaccine in adults aged >/=65 years: a phase 2, randomised, open-label study. Lancet Respir Med 2022;10:392-402, https://doi.org/10.1016/S2213-2600(21)00557-9.
**14**	[14] Schwarz TF, Aggarwal N, Moeckesch B, Schenkenberger I, Claeys C, Douha M, et al. Immunogenicity and safety of an adjuvanted herpes zoster subunit vaccine coadministered with seasonal influenza vaccine in adults aged 50 years or older. The Journal of Infectious Diseases 2017;216:1352-61, https://doi.org/10.1093/infdis/jix481.

	References
**15**	[15] Song JY, Cheong HJ, Tsai TF, Chang H, Choi MJ, Jeon JH, et al. Immunogenicity and safety of concomitant mf59-adjuvanted influenza vaccine and 23-valent pneumococcal polysaccharide vaccine administration in older adults. Vaccine 2015;33:4647-52, https://doi.org/10.1016/j.vaccine.2015.05.003.
**16**	[16] Ortiz JR, Spearman PW, Goepfert PA, Cross K, Buddy Creech C, Chen WH, et al. Safety and immunogenicity of monovalent h7n9 influenza vaccine with as03 adjuvant given sequentially or simultaneously with a seasonal influenza vaccine: a randomized clinical trial. Vaccine 2022;40:3253-62, https://doi.org/10.1016/j.vaccine.2022.03.055.
**17**	[17] Weston WM, Friedland LR, Wu X, Howe B. Vaccination of adults 65 years of age and older with tetanus toxoid, reduced diphtheria toxoid and acellular pertussis vaccine (boostrix®): results of two randomized trials. Vaccine 2012;30:1721-8, https://doi.org/10.1016/j.vaccine.2011.12.055.
**18**	[18] Weston WM, Chandrashekar V, Friedland LR, Howe B. Safety and immunogenicity of a tetanus toxoid, reduced diphtheria toxoid, and acellular pertussis vaccine when co-administered with influenza vaccine in adults. Hum Vaccin 2009;5:858-66, https://doi.org/10.4161/hv.9961.
**19**	[19] Shenyu W, Xiaoqian D, Bo C, Xuan D, Zeng W, Hangjie Z, et al. Immunogenicity and safety of a SARS-cov-2 inactivated vaccine (coronavac) co-administered with an inactivated quadrivalent influenza vaccine: a randomized, open-label, controlled study in healthy adults aged 18 to 59 years in china. Vaccine 2022;40:5356-65, https://doi.org/10.1016/j.vaccine.2022.07.021.
**20**	[20] Frenck RW, Gurtman A, Rubino J, Smith W, van Cleeff M, Jayawardene D, et al. Randomized, controlled trial of a 13-valent pneumococcal conjugate vaccine administered concomitantly with an influenza vaccine in healthy adults. Clinical and Vaccine Immunology 2012;19:1296-303, https://doi.org/10.1128/CVI.00176-12.
**21**	[21] Herbinger K, von Sonnenburg F, Nothdurft HD, Perona P, Borkowski A, Fragapane E, et al. A phase ii study of an investigational tetravalent influenza vaccine formulation combining mf59®. Hum Vaccin Immunother 2014;10:92-9, https://doi.org/10.4161/hv.26495.

eTable3. exclusion articles and their specific reasons

Author / Publication year	Paper	Is included	Exclusion reasons
Schönbeck et al. 2015	Schönbeck Y, Sanders EAM, Hoes AW, Schilder AGM, Verheij TJM, Hak E. Rationale and design of the prevention of respiratory infections and management in children (primakid) study. Vaccine 2005;23:4906-14, https://doi.org/10.1016/j.vaccine.2005.05.021.	no	protocol
Breiman et al. 2009	Breiman RF, Brooks WA, Goswami D, Lagos R, Borja-Tabora C, Lanata CF, et al. A multinational, randomized, placebo-controlled trial to assess the immunogenicity, safety, and tolerability of live attenuated influenza vaccine coadministered with oral poliovirus vaccine in healthy young children. Vaccine 2009;27:5472-9, https://doi.org/10.1016/j.vaccine.2009.07.002.	no	not adult trial
Furumoto et al. 2008	Furumoto A, Ohkusa Y, Chen M, Kawakami K, Masaki H, Sueyasu Y, et al. Additive effect of pneumococcal vaccine and influenza vaccine on acute exacerbation in patients with chronic lung disease. Vaccine 2008;26:4284-9, https://doi.org/10.1016/j.vaccine.2008.05.037.	no	The study population was chronic lung disease patients
Grilli et al. 1997	Grilli G, Fuiano L, Biasio LR, Pregliasco F, Plebani A, Leibovitz M, et al. Simultaneous influenza and pneumococcal vaccination in elderly individuals. Eur J Epidemiol 1997;13:287-91.	no	outcome ambiguous

Author / Publication year	Paper	Is included	Exclusion reasons
Reyes et al. 2012	Reyes MRAL, Dimaano E, Macalalad N, Dbaibo G, Bianco V, Baine Y, et al. The investigational meningococcal serogroups a, c, w-135 and y tetanus toxoid conjugate vaccine (acwy-tt) and the seasonal influenza virus vaccine are immunogenic and well-tolerated when co-administered in adults. Hum Vaccin Immunother 2014;8:881-7, https://doi.org/10.4161/hv.20212.	no	The type combined vaccine is not viral respiratory vaccine
Lum et al. 2010	Lum LCS, Borja-Tabora CF, Breiman RF, Vesikari T, Sablan BP, Chay OM, et al. Influenza vaccine concurrently administered with a combination measles, mumps, and rubella vaccine to young children. Vaccine 2010;28:1566-74, https://doi.org/10.1016/j.vaccine.2009.11.054.	no	not adult trial
McNeil et al. 2007	Mcneil SA, Noya F, Dionne M, Predy G, Meekison W, Ojah C, et al. Comparison of the safety and immunogenicity of concomitant and sequential administration of an adult formulation tetanus and diphtheria toxoids adsorbed combined with acellular pertussis (tdap) vaccine and trivalent inactivated influenza vaccine in adults. Vaccine 2007;25:3464-74, https://doi.org/10.1016/j.vaccine.2006.12.047.	no	outcome ambiguous
Walter et al. 2020	Walter EB, Klein NP, Wodi AP, Rountree W, Todd CA, Wiesner A, et al. Fever after influenza, diphtheria-tetanus-acellular pertussis, and pneumococcal vaccinations. Pediatrics 2020;145, https://doi.org/10.1542/peds.2019-1909.	no	not adult trial

Author / Publication year	Paper	Is included	Exclusion reasons
Schwarz et al. 2013	Schwarz TF, Schmoele-Thoma B. Assessment of functional antibacterial opsonophagocytic antibodies elicited by 13-valent pneumococcal conjugate vaccine administered concomitantly with trivalent influenza vaccine in a randomized clinical trial in adults aged ≥65 years. Vaccine 2013;31:291-4, https://doi.org/10.1016/j.vaccine.2012.10.077.	no	The type combined vaccine is not viral respiratory vaccine
Stefanizzi et al. 2022	Stefanizzi P, Martinelli A, Bianchi FP, Migliore G, Tafuri S. Acceptability of the third dose of anti-SARS-cov-2 vaccine co-administered with influenza vaccine: preliminary data in a sample of italian hcws. Hum Vaccin Immunother 2022;18:1-2, https://doi.org/10.1080/21645515.2021.2011652.	no	Acceptability Survey/ letters
Baj et al. 2022	Baj A, Gasperina DD, Focosi D, Forlani G, Ferrante FD, Novazzi F, et al. Safety and immunogenicity of synchronous covid19 and influenza vaccination. J Clin Virol Plus 2022;2:100082, https://doi.org/10.1016/j.jcvp.2022.100082.	no	outcome ambiguous
Huang et al. 2014	Huang L, Lin T, Chiu C, Chiu N, Chen P, Yeh S, et al. Concomitant administration of live attenuated japanese encephalitis chimeric virus vaccine (je-cv) and measles, mumps, rubella (mmr) vaccine: randomized study in toddlers in taiwan. Vaccine 2014;32:5363-9, https://doi.org/10.1016/j.vaccine.2014.02.085.	no	The type combined vaccine is not viral respiratory vaccine/ not adult trial

Author / Publication year	Paper	Is included	Exclusion reasons
Wijesinghe et al. 2014	Wijesinghe PR, Abeysinghe MRN, Yoksan S, Yao Y, Zhou B, Zhang L, et al. Safety and immunogenicity of live-attenuated japanese encephalitis sa 14-14-2 vaccine co-administered with measles vaccine in 9-month-old infants in sri lanka. Vaccine 2014;32:4751-7, https://doi.org/10.1016/j.vaccine.2014.06.036.	no	The type combined vaccine is not viral respiratory vaccine/ not adult trial
Kaltenböck et al. 2009	Kaltenböck A, Dubischar-Kastner K, Eder G, Jilg W, Klade C, Kollaritsch H, et al. Safety and immunogenicity of concomitant vaccination with the cell-culture based japanese encephalitis vaccine ic51 and the hepatitis a vaccine havrix®1440 in healthy subjects: a single-blind, randomized, controlled phase 3 study. Vaccine 2009;27:4483-9, https://doi.org/10.1016/j.vaccine.2009.05.034.	no	The type combined vaccine is not viral respiratory vaccine
Li et al. 2019	Li Y, Chu SY, Yue C, Wannemuehler K, Xie S, Zhang F, et al. Immunogenicity and safety of measles-rubella vaccine co-administered with attenuated japanese encephalitis sa 14–14–2 vaccine in infants aged 8 months in china: a non-inferiority randomised controlled trial. The Lancet Infectious Diseases 2019;19:402-9, https://doi.org/10.1016/S1473-3099(18)30650-9.	no	The type combined vaccine is not viral respiratory vaccine/ not adult trial

**Author / Publication year**	**Paper**	**Is included**	**Exclusion reasons**
Angsuwatcharakon et al. 2020	Angsuwatcharakon P, Ratananpinit N, Yoksan S, Saengseesom W, Sriaksorn R, Raksahket N, et al. Immunogenicity and safety of two-visit, intradermal pre-exposure rabies prophylaxis simultaneously administrated with chimeric live-attenuated japanese encephalitis vaccine in children living in rabies and japanese encephalitis endemic country. Vaccine 2020;38:5015-20, https://doi.org/10.1016/j.vaccine.2020.05.054.	no	The type combined vaccine is not viral respiratory vaccine/ not adult trial
Gatchalian et al. 2008	Gatchalian S, Yao Y, Zhou B, Zhang L, Yoksan S, Kelly K, et al. Comparison of the immunogenicity and safety of measles vaccine administered alone or with live, attenuated japanese encephalitis sa 14-14-2 vaccine in philippine infants. Vaccine 2008;26:2234-41, https://doi.org/10.1016/j.vaccine.2008.02.042.	no	The type combined vaccine is not viral respiratory vaccine/ not adult trial
Nasveld et al. 2010	Nasveld PE, Marjason J, Bennett S, Aaskov J, Elliott S, Mccarthy K, et al. Concomitant or sequential administration of live attenuated japanese encephalitis chimeric virus vaccine and yellow fever 17d vaccine. Human Vaccines 2014;6:906-14, https://doi.org/10.4161/hv.6.11.12854.	no	The type combined vaccine is not viral respiratory vaccine

Author / Publication year	Paper	Is included	Exclusion reasons
Liu et al. 2021	Liu X, Yang W, Zhang C, Wu H, Wang R, Ding Q, et al. Immunogenicity and safety of an inactivated enterovirus 71 vaccine co-administered with measles-mumps-rubella vaccine and live-attenuated japanese encephalitis vaccine: a phase 4, single-center, randomized controlled trial. Hum Vaccin Immunother 2021;17:5348-54, https://doi.org/10.1080/21645515.2021.2010428.	no	The type combined vaccine is not viral respiratory vaccine/ not adult trial
Jelinek et al. 2015	Jelinek T, Burchard GD, Dieckmann S, Bühler S, Paulke-Korinek M, Nothdurft HD, et al. Short-term immunogenicity and safety of an accelerated pre-exposure prophylaxis regimen with japanese encephalitis vaccine in combination with a rabies vaccine: a phase iii, multicenter, observer-blind study. J Travel Med 2015;22:225-31, https://doi.org/10.1111/jtm.12210.	no	The type combined vaccine is not viral respiratory vaccine
Jelinek-2 et al. 2015	Jelinek T, Cramer JP, Dieckmann S, Hatz C, Paulke-Korinek M, Alberer M, et al. Evaluation of rabies immunogenicity and tolerability following a purified chick embryo cell rabies vaccine administered concomitantly with a japanese encephalitis vaccine. Travel Med Infect Dis 2015;13:241-50, https://doi.org/10.1016/j.tmaid.2015.05.008.	no	The type combined vaccine is not viral respiratory vaccine

Author / Publication year	Paper	Is included	Exclusion reasons
Pengsaa et al. 2009	Pengsaa K, Limkittikul K, Sabchareon A, Ariyasriwatana C, Chanthavanich P, Attanath P, et al. A three-year clinical study on immunogenicity, safety, and booster response of purified chick embryo cell rabies vaccine administered intramuscularly or intradermally to 12- to 18-month-old thai children, concomitantly with japanese encephalitis vaccine. Pediatr Infect Dis J 2009;28:335-7, https://doi.org/10.1097/INF.0b013e3181906351.	no	The type combined vaccine is not viral respiratory vaccine/not adult trial
Cramer et al. 2016	One-year immunogenicity kinetics and safety of a purified chick embryo cell rabies vaccine and an inactivated vero cell-derived japanese encephalitis vaccine administered concomitantly according to a new, 1-week, accelerated primary series. J Travel Med 2016, https://doi.org/10.1093/jtm/taw011.	no	The type combined vaccine is not viral respiratory vaccine/not adult trial
Maréchal et al. 2018	Maréchal C, Lal H, Poder A, Ferguson M, Enweonye I, Heineman TC, et al. Immunogenicity and safety of the adjuvanted recombinant zoster vaccine co-administered with the 23-valent pneumococcal polysaccharide vaccine in adults ≥50 years of age: a randomized trial. Vaccine 2018;36:4278-86, https://doi.org/10.1016/j.vaccine.2018.05.110.	no	The type combined vaccine is not viral respiratory vaccine

Author / Publication year	Paper	Is included	Exclusion reasons
Strezova et al. 2019	Strezova A, Lal H, Enweonye I, Campora L, Beukelaers P, Segall N, et al. The adjuvanted recombinant zoster vaccine co-administered with a tetanus, diphtheria and pertussis vaccine in adults aged ≥50 years: a randomized trial. Vaccine 2019;37:5877-85, https://doi.org/10.1016/j.vaccine.2019.08.001.	no	The type combined vaccine is not viral respiratory vaccine
Calabrese et al. 2020	Calabrese LH, Abud Mendoza C, Lindsey SM, Lee SH, Tatulych S, Takiya L, et al. Live zoster vaccine in patients with rheumatoid arthritis treated with tofacitinib with or without methotrexate, or adalimumab with methotrexate: a post hoc analysis of data from a phase iiib/iv randomized study. Arthritis Care Res (Hoboken) 2020;72:353-9, https://doi.org/10.1002/acr.24010.	no	not vaccine efficacy trial
Blatter et al. 2012	Blatter MM, Klein NP, Shepard JS, Leonardi M, Shapiro S, Schear M, et al. Immunogenicity and safety of two tetravalent (measles, mumps, rubella, varicella) vaccines coadministered with hepatitis a and pneumococcal conjugate vaccines to children twelve to fourteen months of age. Pediatr Infect Dis J 2012;31:e133-40, https://doi.org/10.1097/INF.0b013e318259fc8a.	no	The type combined vaccine is not viral respiratory vaccine/not adult trial

Author / Publication year	Paper	Is included	Exclusion reasons
Hata et al. 2019	Hata A, Ishioka T, Oishi K, Katayama T, Ohkubo T. Altered immunogenicity of 23-valent pneumococcal polysaccharide vaccine in elderly patients with diabetes who revealed lower responses to concomitant administration of biken varicella zoster vaccine: results of post hoc analysis of a randomized double-blind trial. J Diabetes Complications 2019;33:243-8, https://doi.org/10.1016/j.jdiacomp.2018.11.003.	no	The type combined vaccine is not viral respiratory vaccine
MacIntyre et al. 2010	Macintyre CR, Egerton T, Mccaughey M, Parrino J, Campbell BV, Su SC, et al. Concomitant administration of zoster and pneumococcal vaccines in adults >/=60 years old. Hum Vaccin 2010;6:894-902, https://doi.org/10.4161/hv.6.11.12852.	no	The type combined vaccine is not viral respiratory vaccine
Min et al. 2022	Min J, Mwakingwe-Omari A, Riley M, Molo LY, Soni J, Girard G, et al. The adjuvanted recombinant zoster vaccine co-administered with the 13-valent pneumococcal conjugate vaccine in adults aged ≥50 years: a randomized trial. J Infect 2022;84:490-8, https://doi.org/10.1016/j.jinf.2021.12.033.	no	The type combined vaccine is not viral respiratory vaccine
Rome et al. 2021	Rome BN, Feldman WB, Fischer MA, Desai RJ, Avorn J. Influenza vaccine uptake in the year after concurrent vs separate influenza and zoster immunization. Jama Netw Open 2021;4:e2135362, https://doi.org/10.1001/jamanetworkopen.2021.35362.	no	not randomized controlled trial

Author / Publication year	Paper	Is included	Exclusion reasons
Li et al. 2022	Li W, Wang F, Guo R, Bian Z, Song Y. Targeting macrophages in hematological malignancies: recent advances and future directions. J Hematol Oncol 2022;15, https://doi.org/10.1186/s13045-022-01328-x.	no	not randomized controlled trial
Baber et al. 2022	Baber J, Arya M, Moodley Y, Jaques A, Jiang Q, Swanson KA, et al. A phase 1/2 study of a respiratory syncytial virus prefusion f vaccine with and without adjuvant in healthy older adults. The Journal of Infectious Diseases 2022, https://doi.org/10.1093/infdis/jiac189.	no	Phase 1/2 Study
Falsey 2022	Falsey AR, Walsh EE, Scott DA, Gurtman A, Zareba A, Jansen KU, et al. Phase 1/2 randomized study of the immunogenicity, safety, and tolerability of a respiratory syncytial virus prefusion f vaccine in adults with concomitant inactivated influenza vaccine. The Journal of Infectious Diseases 2022;225:2056-66, https://doi.org/10.1093/infdis/jiab611.	no	Phase 1/2 Study

A

eTable4. Grading of recommendations, assessment, development and evaluations (GRADE) in immunogenicity of SARS-COV-2 vaccine group

Outcome	No of studies	Study design	Risk of bias	Imprecision	Inconsistency	Indirectness	Publication bias	Certainty
GMT	10	RCT	Not serious	high	High	Low	Low	⊕◯◯◯ Very Low
GMFR	4	RCT	Not serious	high	High	Low	Low	⊕◯◯◯ Very Low
SCR	10	RCT	Not serious	moderate	Low	Low	Low	⊕⊕⊕◯ Moderate

Risk of bias: According to Assessment of risk of bias for included studies. 5 articles upgraded to some concerns in the randomization pross evaluation. 7 articles were assigned to some concerns in deviations from intended interventions. 5 articles generating high-risk assessments and 5 generating moderate in the measurement of the outcome. 1 article upgraded to some concerns the selection of the reported result.

Imprecision: To assess imprecision of the estimates, we set the thresholds for every worthwhile difference in each term. A ‘major concerned point’ is assigned to a result if the 95% confidence interval extends beyond the area of equivalence on the opposite side of the no effect line as the point estimate, so that the estimated treatment effect is compatible with clinically important effects in both directions. A rating of ‘some concerned point’ is assigned if the confidence interval extends into but not beyond the area of equivalence on the opposite side of the no effect line. There are ‘No concerns point’ if the confidence interval is entirely on one side of the no effect line, or if it is entirely within the area of equivalence.

Low: “major concerned point” is less than 25% or “some concerned point” is less than 50% of total worthwhile difference

moderate: “major concerned point” is greater than or equal to 25% of total worthwhile difference, but less than 50% or “some concerned point” is greater than or equal to 50% of total worthwhile difference, but less than 75%

high: “major concerned point” is greater than or equal to 50% or “some concerned point” is greater than or equal to 75% of total worthwhile difference

Inconsistency: Heterogeneity was considered 'low', 'moderate', 'high' for estimated I^2 under 25%, between 25% and 50%, and over 50% in each term, respectively, in each term..

Indirectness: The included studies were deemed directly relevant, as they aligned with our research questions and adequately reported outcomes, indicating no indirectness.

Across-studies bias: No significant evidence of publication bias was observed, except in the case of the Chills of seasonal influenza vaccine studies. This was determined as the funnel plot did not indicate the presence of bias ($p>0.05$). The included studies had minimal missing outcomes, and the potential for reporting bias was assessed as low.

eTable5. Grading of recommendations, assessment, development and evaluations (GRADE) in immunogenicity of seasonal influenza vaccine group

Outcome	No of studies	Study design	Risk of bias	Imprecision	Inconsistency	Indirectness	Publication bias	Certainty
A/H1N1-SCR	23	RCT	Not serious	low	Low	Low	Low	⊕⊕⊕⊕ High
A/H1N1-SPR	17	RCT	Not serious	low	Moderate	Low	Low	⊕⊕⊕○ Moderate
A/H1N1-GMT	25	RCT	Not serious	high	High	Low	Low	⊕○○○ Very Low
A/H3N2-SCR	23	RCT	Not serious	low	Moderate	Low	Low	⊕⊕⊕○ Moderate
A/H3N2-SPR	17	RCT	Not serious	low	Low	Low	Low	⊕⊕⊕⊕ High
A/H3N2-GMT	25	RCT	Not serious	high	High	Low	Low	⊕○○○ Very Low
B-SCR	37	RCT	Not serious	low	Low	Low	Low	⊕⊕⊕⊕ High
B-SPR	27	RCT	Not serious	low	Low	Low	Low	⊕⊕⊕⊕ High
B-GMT	38	RCT	Not serious	high	High	Low	Low	⊕○○○ Very Low

eTable6. Grading of recommendations, assessment, development and evaluations (GRADE) in adverse event incidence of SARS-COV-2 vaccine group

Outcome	No of studies	Study design	Risk of bias	Imprecision	Inconsistency	Indirectness	Publication bias	Certainty
Total ADs								
Fever	10	RCT	Not serious	low	Low	Low	Low	⊕⊕⊕⊕ High
Fatigue	10	RCT	Not serious	low	Low	Low	Low	⊕⊕⊕⊕ High
Headache	10	RCT	Not serious	low	Low	Low	Low	⊕⊕⊕⊕ High
Malaise	9	RCT	Not serious	low	Moderate	Low	Low	⊕⊕⊕○ Moderate
Muscle pain	10	RCT	Not serious	low	Moderate	Low	Low	⊕⊕⊕○ Moderate
Gas	10	RCT	Not serious	low	Moderate	Low	Low	⊕⊕⊕○ Moderate
Chills	7	RCT	Not serious	low	Low	Low	Low	⊕⊕⊕⊕ High
Pain	10	RCT	Not serious	low	Low	Low	Low	⊕⊕⊕⊕ High
Tenderness	3	RCT	Not serious	low	Low	Low	Low	⊕⊕⊕⊕ High
Erythra	10	RCT	Not serious	low	Low	Low	Low	⊕⊕⊕⊕ High
Swelling	9	RCT	Not serious	low	Low	Low	Low	⊕⊕⊕⊕ High
Induration	8	RCT	Not serious	low	Low	Low	Low	⊕⊕⊕⊕ High

Outcome	No of studies	Study design	Risk of bias	Imprecision	Inconsistency	Indirectness	Publication bias	Certainty
>Grade-3 ADs								
Fever-G3	7	RCT	Not serious	low	Low	Low	Low	⊕⊕⊕⊕ High
Fatigue - G3	6	RCT	Not serious	low	Low	Low	Low	⊕⊕⊕⊕ High
Headache -G3	7	RCT	Not serious	low	Low	Low	Low	⊕⊕⊕⊕ High
Malaise-G3	9	RCT	Not serious	low	Low	Low	Low	⊕⊕⊕⊕ High
Muscle pain-G3	5	RCT	Not serious	low	Low	Low	Low	⊕⊕⊕⊕ High
Pain-G3	5	RCT	Not serious	low	Low	Low	Low	⊕⊕⊕⊕ High

eTable7. Grading of recommendations, assessment, development and evaluations (GRADE) in adverse event incidence of seasonal influenza vaccine group

Outcome	No of studies	Study design	Risk of bias	Imprecision	Inconsistency	Indirectness	Publication bias	Certainty
Total ADs								
Fever	17	RCT	Not serious	low	High	Low	Low	⊕⊕○○ Low
Fatigue	22	RCT	Not serious	moderate	High	Low	Low	⊕○○○ Very Low
Headache	23	RCT	Not serious	moderate	High	Low	Low	⊕○○○ Very Low
Chills	16	RCT	Not serious	Low	High	Low	High	⊕○○○ Very Low
Rash	5	RCT	Not serious	low	Low	Low	Low	⊕⊕⊕⊕ High
gas	18	RCT	Not serious	moderate	High	Low	Low	⊕○○○ Very Low
Arthralgia	20	RCT	Not serious	moderate	High	Low	Low	⊕○○○ Very Low
Muscle pain	21	RCT	Not serious	low	High	Low	Low	⊕⊕○○ Low
Malaise	10	RCT	Not serious	low	High	Low	Low	⊕⊕○○ Low
pain	24	RCT	Not serious	moderate	High	Low	Low	⊕○○○ Very Low
redness	24	RCT	Not serious	low	High	Low	Low	⊕⊕○○ Low
Itch	13	RCT	Not serious	low	Low	Low	Low	⊕⊕⊕⊕ High

Outcome	No of studies	Study design	Risk of bias	Imprecision	Inconsistency	Indirectness	Publication bias	Certainty
Swelling	24	RCT	Not serious	low	High	Low	Low	⊕⊕◯◯ Low

Outcome	No of studies	Study design	Risk of bias	Imprecision	Inconsistency	Indirectness	Publication bias	Certainty
>Grade-3 ADs								
Fatigue-G3	9	RCT	Not serious	low	Low	Low	Low	⊕⊕⊕⊕ High
Headache-G3	9	RCT	Not serious	low	Low	Low	Low	⊕⊕⊕⊕ High
Chills-G3	5	RCT	Not serious	low	Low	Low	Low	⊕⊕⊕⊕ High
gas-G3	7	RCT	Not serious	low	Low	Low	Low	⊕⊕⊕⊕ High
Muscle pain-G3	7	RCT	Not serious	low	Low	Low	Low	⊕⊕⊕⊕ High
Malaise-G3	7	RCT	Not serious	low	Low	Low	Low	⊕⊕⊕⊕ High
pain-G3	7	RCT	Not serious	Low	Low	Low	Low	⊕⊕⊕⊕ High
redness-G3	4	RCT	Not serious	low	Low	Low	Low	⊕⊕⊕⊕ High
Swelling-G3	5	RCT	Not serious	low	Low	Low	Low	⊕⊕⊕⊕ High

eTable8. immunogenicity statistics detail for each included group in SARS-COV-2 vaccine group

Name	n-con	n-seq	COVID-19-SCR		COVID-19-GMT				COVID-19-GMFR			
			re-con	re-seq	mean-con	mean-seq	SD-con	SD-seq	mean-con	mean-seq	SD-con	SD-seq
Toback 2022-young	168	300	164	297	31516.9	47564.3	5714.55	5561.05	272.3	425	55.6	52.55
Toback 2022-aged	10	114	10	113	26876.1	37892.8	15803.45	7867.6	214	335.9	189.05	68.35
Lazarus 2021-ChA+CQV	60	64	49	53	18.1	19.4	1.55	2.45				
Lazarus 2021-BNT+CQV	68	79	63	!64	47.1	52.9	4.85	3.8				
Lazarus 2021-ChA+TV	72	70	64	~64	19.9	19.2	2	2				
Lazarus 2021-BNT+TV	41	38	~40	37	44.8	45.5	5.2	5.75				
Lazarus 2021-ChA+RQV	63	62	^56	~49	19.9	23.3	2.45	2.8				
Lazarus 2021-BNT+RQV	29	28	26	25	40.6	50	6.8	5.4				
Izikson 2022	96	102	89	97	7634	7904	1298.5	1097	13.7	14.2	2.8	2.45
wang 2022	232	227	216	216	27.5	38.1	3.35	4.8	13.8	19	1.7	2.4

n-con= Number of participants in the concomitant vaccination group, n-seq= Number of participants in the sequence vaccination group, re-con= Number of responders in the concomitant vaccination group, re-seq= Number of responders in the sequence vaccination group, mean-con= Mean serum immunogenicity of concomitant vaccination participants, mean-seq= Mean serum immunogenicity of sequence vaccination participants, SD-con= Standard deviation serum immunogenicity of concomitant vaccination participants, SD-seq= Standard deviation serum immunogenicity of sequence vaccination participants.
~ 1participant decreased, ^ 2participants decreased, & 3participants decreased, * 4-5participants decreased

eTable9. immunogenicity statistics detail for each included group in seasonal influenza vaccine group

			SIV-H1N1-SCR		SIV-H3N2-SCR		SIV-B-SCR			
Name	n-con	n-seq	re-con	re-seq	re-con	re-seq	re-con	re-seq	re-con	re-seq
Schwarz 2011	not reported									
Levin 2018	400	401	167	166	305	299	151	150	124	123
Anyinam 2017	162	170	101	110	61	62	54	60	55	73
Sadoff 2021	85	89	36	38	47	50	36	33	39	39
Kerzner 2007	356	353	170	181	276	274	211	233		
Severance 2022	569	561	276	271	158	142	166	161	177	171
Nakashima 2018	not reported									
Thompson 2019	427	430	125	104	119	136	91	96	99	106
Toback 2022-young	201	201	153	143	136	125	29	20	65	65
Toback 2022-aged	16	13	12	10	8	7	2	4		
Lazarus 2021-ChA+CQV	61	64	20	17	~21	14	7	4	9	13
Lazarus 2021-BNT+CQV	68	70	22	19	20	23	6	2	9	^9
Lazarus 2021-ChA+TV	73	71	16	7	8	~6	1	2		
Lazarus 2021-BNT+TV	40	37	6	2	5	3	1	1		
Lazarus 2021-ChA+RQV	61	64	28	40	41	37	13	16	23	22
Lazarus 2021-BNT+RQV	29	28	19	11	21	17	11	6	17	7
Song 2017	373	382	220	224	226	190				
Zimmermann 2013	not reported									
Izikson 2022	96	86	66	62	42	41				
Schwarz 2017	384	394	232	240	136	139				
Song-2 2015	107	56	40	25	37	19				
Ortiz 2022	59	82	31	47	29	51				
Weston 2012	708	672	414	377	560	494				
Weston 2009	108	102	37	32	72	64				

Name	n-con	n-seq	SIV-H1N1-SCR		SIV-H3N2-SCR		SIV-B-SCR			
			re-con	re-seq	re-con	re-seq	re-con	re-seq	re-con	re-seq
Wang 2022	234	228	216	199	216	214				
Herbinger 2014	189	181	136	143	134	145				
Frenck 2012		not reported								

			SIV-H1N1-SPR		SIV-H3N2-SPR		SIV-B-SPR			
**Name**	**n-con**	**n-seq**	**re-con**	**re-seq**	**re-con**	**re-seq**	**re-con**	**re-seq**	**re-con**	**re-seq**
**Schwarz 2011**	548	546	515	514	&526	~531	449	444		
**Levin 2018**	409	405	385	388	392	389	272	249	220	201
**Anyinam 2017**	163	170	157	160	~128	131	~162	169	~154	167
**Sadoff 2021**	85	89	70	69	75	77	48	51	47	45
**Kerzner 2007**	363	363	323	331	314	309	312	326		
**Severance 2022**	576	567	495	480	446	449	317	311	302	288
**Nakashima 2018**	81	77	68	60	66	68	33	45	49	48
**Thompson 2019**	427	430	387	384	420	415	210	200	281	272
**Toback 2022-young**	not reported									
**Toback 2022-aged**	not reported									
**Lazarus 2021-ChA+CQV**	not reported									
**Lazarus 2021-BNT+CQV**	not reported									
**Lazarus 2021-ChA+TV**	not reported									
**Lazarus 2021-BNT+TV**	not reported									
**Lazarus 2021-ChA+RQV**	not reported									
**Lazarus 2021-BNT+RQV**	not reported									
**Song 2017**	373	382	330	350	369	379	271	276		
**Zimmermann 2013**	not reported									
**Izikson 2022**	100	92	94	87	96	91	99	90	99	91
**Schwarz 2017**	384	394	347	360	292	295	372	382	383	393
**Song-2 2015**	107	56	73	46	102	55	84	43		
**Ortiz 2022**	59	82	59	78	55	79	15	34	32	60
**Weston 2012**	711	675	669	643	694	663	685	654		
**Weston 2009**	108	103	92	88	104	95	107	101		

			SIV-H1N1-SPR		SIV-H3N2-SPR		SIV-B-SPR			
**Name**	**n-con**	**n-seq**	**re-con**	**re-seq**	**re-con**	**re-seq**	**re-con**	**re-seq**	**re-con**	**re-seq**
**Wang 2022**	234	228	219	199	233	228	197	195	220	221
**Herbinger 2014**	189	181	187	180	186	179	147	147		

Name	n-con	n-seq	SIV-H1N1-GMT mean-con	mean-seq	SD-con	SD-seq	SIV-H3N2-GMT mean-con	mean-seq	SD-con	SD-seq	SIV-B-GMT mean-con	mean-seq	SD-con	SD-seq	mean-con	mean-seq	SD-con	SD-seq
**Schwarz 2011**	548	546	195.5	191.9	19.9	19.55	&327.4	^413.2	35.85	45.15	90.4	88.5	9.3	9.15				
**Levin 2018**	409	405	207.3	200.5	23.05	21.3	298.8	272	37	32.5	46.7	43.9	5	4.45	33.9	32.3	409	405
**Anyinam 2017**	not reported																	
**Sadoff 2021**	85	89	215	168	64.5	50.5	98	80	29	21	39	40	9	5	35	35	85	89
**Kerzner 2007**	363	363	122.9	134.2	16.1	21.95	162.7	150.5	24	22.35	119.8	136.5	16.15	17.1				
**Severance 2022**	576	567	127.35	112.6	18.5	12.25	90.11	83.63	10.5	9.3	35.58	37.13	3.4	3.6	33.82	33.03	576	567
**Nakashima 2018**	not reported																	
**Thompson 2019**	427	430	115	113	11.4	11.5	226	196	21.2	19	28	26	2.65	2.5	45	43	427	430
**Toback 2022-young**	201	201	186.5	170.6	38.8	32.85	243.7	226.2	32.3	33.65	9.7	9.4	2	1.75	38.8	39	201	201
**Toback 2022-aged**	16	13	159	112.8	92.15	142.9	173.2	199	98	142.85	11.8	21.9	8.95	23.75				
**Lazarus 2021-ChA+CQV**	61	64	6.1	6.1	0.8	0.8	8.2	7.4	0.8	0.7	2.7	2.8	0.4	0.4	3.6	3.8	61	64
**Lazarus 2021-BNT+CQV**	68	70	6.7	6.3	0.7	0.8	8.5	7.9	0.8	0.7	2.5	2.4	2.3	0.3	3.8	3.9	68	70
**Lazarus 2021-ChA+TV**	73	71	5.2	4.6	0.55	0.55	6.3	6.7	0.6	0.35	2.3	2.1	0.25	0.25				
**Lazarus 2021-BNT+TV**	41	37	5.9	5.2	0.85	0.9	7.5	6.3	0.9	0.95	2.4	2.3	0.3	0.3				
**Lazarus 2021-ChA+RQV**	63	64	7.9	9.3	0.95	1.1	10	9.5	0.95	1.05	3.7	3.3	0.5	0.5	4.9	4.7	63	64
**Lazarus 2021-BNT+RQV**	29	28	10.9	9	1.7	1.85	12.6	12	1.35	1.3	3.3	3	0.75	0.55	5.6	5	29	28
**Song 2017**	373	382	169.4	136.5	23.1	16.35	315.5	238.2	30.5	23.35	45	46.7	3.75	3.75				
**Zimmermann 2013**	463	448	177	189	26	27.5	291	338	39	44.5	305	308	32.5	34				
**Izikson 2022**	96	86	363	366	50	109.5	286	315	59.5	64.5	429	471	87.5	105	377	390	96	86
**Schwarz 2017**	384	394	187.5	194.3	22.05	22.55	63.7	65.9	5.7	5.85	170.2	181.6	14.75	15.55	423.5	413.9	384	394
**Song-2 2015**	55	56	55.5	70.6	18.3	19.55	115.3	168.3	29.4	41.25	59.2	60.1	13.4	12.5				
**Ortiz 2022**	59	53	547.7	503.6	132.55	171.25	284	289.8	99.2	106.1	20.8	23.05	6.6	7.3	46.1	61.8	55	52
**Weston 2012**	711	675	189.1	181.6	18.3	16.9	368.7	337.3	32.2	29.35	210.1	222.8	16.3	17.6				

Name	n-con	n-seq	SIV-H1N1-GMT mean-con	mean-seq	SD-con	SD-seq	SIV-H3N2-GMT mean-con	mean-seq	SD-con	SD-seq	SIV-B-GMT mean-con	mean-seq	SD-con	SD-seq	mean-con	mean-seq	SD-con	SD-seq
**Weston 2009**	108	103	79.9	89.3	16.65	18.35	333.6	276.8	73.85	74.2	221.2	214.3	40.55	43.2				
**Wang 2022**	234	228	462	342	99.87	80.65	724.8	703.3	101.75	98.2	95.6	100.8	14.55	15.7	154	161.7	234	228
**Herbinger 2014**	189	181	565	702	150.5	192	292	425	71	106.5	110	116	28	30				
**Frenck 2012**	530	531	331.8	330.6	32.8	134.05	443.2	477.7	45.7	53.25	67.7	77.8	6.9	7.8				

n-con= Number of participants in the concomitant vaccination group, n-seq= Number of participants in the sequence vaccination group, re-con= Number of responders in the concomitant vaccination group, re-seq= Number of responders in the sequence vaccination group, mean-con= Mean serum immunogenicity of concomitant vaccination participants, mean-seq= Mean serum immunogenicity of sequence vaccination participants, SD-con= Standard deviation serum immunogenicity of concomitant vaccination participants, SD-seq= Standard deviation serum immunogenicity of sequence vaccination participants.

~ 1participant decreased, ^ 2participants decreased, & 3participants decreased, * 4-5participants decreased

eTable10. System ADs statistics detail for each included group in SARS-COV-2 vaccine group

Name			Fever-total		Fever-Grade 3+		Fatigue-total		Fatigue-Grade 3+		Headache-total		Headache-Grade 3+		Chills-total	
	n-con	n-seq	re-con	re-seq	re-con	re-seq	re-con	re-seq	re-con	re-seq	re-con	re-seq	re-con	re-seq	re-con	re-seq
Toback 2022-young	190	931	8	20	1	5	56	204	3	4	48	248	1	6		
Toback 2022-aged	15	228	1	2	0	0	1	21	0	0	3	36	1	4		
Lazarus 2021-ChA+CQV	58-63	59-62	11	13	1	0	41	36	4	1	36	31	2	0	11	10
Lazarus 2021-BNT+CQV	64-67	66-67	14	15	1	0	45	44	1	2	42	36	1	0	20	19
Lazarus 2021-ChA+TV	66-70	66-68	9	11	1	0	36	29	0	0	22	21	1	0	9	11
Lazarus 2021-BNT+TV	39-41	35-36	2	4	0	0	17	19	0	0	14	13	0	0	4	4
Lazarus 2021-ChA+RQV	59-61	58-61	5	9	1	0	37	30	1	0	27	21	1	0	6	11
Lazarus 2021-BNT+RQV	23-27	23-27	6	3	0	0	22	17	0	1	18	12	0	0	6	2
Izikson 2022	100	104	12	11	2	2	62	72	6	6	51	54	0	5	33	21
wang 2022	240	240	2	3	1	0	1	1			1	2				

n-con= Number of participants in the concomitant vaccination group, n-seq= Number of participants in the sequence vaccination group, re-con= Number of responders in the concomitant vaccination group, re-seq= Number of responders in the sequence vaccination group.

eTable11. System ADs statistics detail for each included group in SARS-COV-2 vaccine group

			Local pain-total		Local pain-Grade 3+		Tenderness-total		Tenderness-Grade 3+		Erythema-total		Erythema-Grade 3+		Swelling-total	
**Name**	**n-con**	**n-seq**	**re-con**	**re-seq**	**re-con**	**re-seq**	**re-con**	**re-seq**	**re-con**	**re-seq**	**re-con**	**re-seq**	**re-con**	**re-seq**	**re-con**	**re-seq**
**Toback 2022-young**	190	931	79	310	1	0	127	537	4	11	2	20	0	0	2	10
**Toback 2022-aged**	15	228	2	28	0	0	6	78	0	0	0	3	0	0	0	0
**Lazarus 2021-ChA+CQV**	58-63	60-62	45	42	1	1					18	13			8	17
**Lazarus 2021-BNT+CQV**	62-68	64-71	63	63	1	3					14	14			13	14
**Lazarus 2021-ChA+TV**	67-71	66-72	32	37	0	0					8	6			8	5
**Lazarus 2021-BNT+TV**	39-40	35-38	24	25	0	0					6	6			4	4
**Lazarus 2021-ChA+RQV**	59-60	57-62	39	45	0	0					11	13			8	10
**Lazarus 2021-BNT+RQV**	24-25	24-27	23	23	1	0					3	4			3	6
**Izikson 2022**	100	104	82	87	4	2	3	3	0	0	22	31	4	7	14	27
**wang 2022**	240	240	27	28	0	0					2	5			3	9

Name	n-con	n-seq	Swelling-Grade3+ re-con	re-seq	Induration-total re-con	re-seq
**Toback 2022-young**	190	931	0	0		
**Toback 2022-aged**	15	228	0	0		
**Lazarus 2021-ChA+CQV**	58-63	60-62			17	16
**Lazarus 2021-BNT+CQV**	62-68	64-71			14	19
**Lazarus 2021-ChA+TV**	67-71	66-72			7	10
**Lazarus 2021-BNT+TV**	39-40	35-38			5	12
**Lazarus 2021-ChA+RQV**	59-60	57-62			8	11
**Lazarus 2021-BNT+RQV**	24-25	24-27			2	6
**Izikson 2022**	100	104	1	1	13	22
**wang 2022**	240	240			6	4

n-con= Number of participants in the concomitant vaccination group, n-seq= Number of participants in the sequence vaccination group, re-con= Number of responders in the concomitant vaccination group, re-seq= Number of responders in the sequence vaccination group.

eTable12. System ADs statistics detail for each included group in seasonal influenza vaccine group

Name	n-con	n-seq	Fever-total		Fatigue-total		Fatigue-Grade 3+		Headache-total		Headache-Grade 3+		Chills-total		Chills-Grade3+		Erythra	
			re-con	re-seq	re-con	re-seq	re-con	re-seq	re-con	re-seq	re-con	re-seq	re-con	re-seq	re-con	re-seq	re-con	re-seq
Schwarz 2011	431-476	433-483	18	14	178	154			154	139			61	40			15	15
Levin 2018	not reoprted																	
Anyinam 2017	173	176	2	0	39	30	2	1	20	21	2	0	10	6	1	0		
Sadoff 2021	90	90	7	0	44	15			35	12			33	4				
Kerzner 2007	378	376							8	17								
Severance 2022	600	596			163	179			129	141								
Nakashima 2018	79-81	76-79	2	3	9	19			4	5							2	2
Thompson 2019	439	437			1	1			0	3							1	1
Toback 2022 (combined)	205	199	9	3	57	58	2	3	51	43	1	0						
Lazarus 2021-ChA+CQV	53-63	51-60	2	0	41	21	4	0	36	16	2	0	11	5				
Lazarus 2021-BNT+CQV	57-67	41-57	0	2	45	22	1	0	42	15	1	0	20	7				
Lazarus 2021-ChA+TV	61-70	63-69	1	0	36	10	0	0	22	13	1	0	9	3				
Lazarus 2021-BNT+TV	37-41	30-37			17	7	0	0	14	6	0	0	4	0				
Lazarus 2021-ChA+RQV	58-61	52-59			37	13	1	0	27	10	1	0	6	1				
Lazarus 2021-BNT+RQV	19-27	18-23			22	4	0	0	18	5	0	0	6	0				
Song 2017	373	382	3	1	102	81			67	50			44	28				
Zimmermann 2013	not reoprted																	
Izikson 2022	100	89	12	1	62	26	6	1	54	18	0	0	32	5	2	0		
Schwarz 2017	409	411	63	18	150	52	15	4	122	57	5	2	101	30	12	0		
Song-2 2015	107	56	2	0	22	5			19	1			24	2				
Ortiz 2022	62	53	13	0	25	11			17	7								

Name	n-con	n-seq	Fever-total		Fatigue-total		Fatigue-Grade 3+		Headache-total		Headache-Grade 3+		Chills-total		Chills-Grade3+		Erythra	
			re-con	re-seq	re-con	re-seq	re-con	re-seq	re-con	re-seq	re-con	re-seq	re-con	re-seq	re-con	re-seq	re-con	re-seq
**Weston 2012**	726	707	54	52	220	165	22	15	225	197	24	25	70	44	8	5		
**Weston 2009**	110	108	4	3	20	18	0	1	17	17	2	1	5	4	0	0		
**Wang 2022**	240	240	2	3	1	1			2	2							1	1
**Herbinger 2014**	196	195	4	8	51	72			41	66			8	20				
**Frenck 2012**	261-399	259-382	9	3	226	188			263	216			96	61			13	13

Name	n-con	n-seq	Gastro-total		Gastro-grade 3+		Arthralgia-total		Arthralgia-grade 3+		Muscle pain-total		Muscle pain-grade 3+		Malaise-total		Malaise-grade 3+	
			re-con	re-seq	re-con	re-seq	re-con	re-seq	re-con	re-seq	re-con	re-seq	re-con	re-seq	re-con	re-seq	re-con	re-seq
Schwarz 2011	431-476	433-483	89	81			73	59			126	76						
Levin 2018	not reoprted																	
Anyinam 2017	173	176	12	16	1	1	16	11	0	0	20	15	1	0				
Sadoff 2021	90	90	13	2			32	5			41	15						
Kerzner 2007	378	376																
Severance 2022	600	596					56	69			142	127						
Nakashima 2018	79-81	76-79					11	11										
Thompson 2019	439	437	2	4			0	0			2	1						
Toback 2022 (combined)	205	199	11	13	0	0	15	3	0	0	58	40	0	0	30	33	2	2
Lazarus 2021-ChA+CQV	53-63	51-60	24	10	0	0	18	5	0	0	28	7	1	0	25	9	3	0
Lazarus 2021-BNT+CQV	57-67	41-57	23	5	0	1	19	4	0	0	37	6	1	0	28	10	1	0
Lazarus 2021-ChA+TV	61-70	63-69	7	2	0	0	14	5	0	0	24	11	0	0	14	7	1	0
Lazarus 2021-BNT+TV	37-41	30-37	2	2	0	0	3	1	0	0	6	3	0	0	4	4	0	0
Lazarus 2021-ChA+RQV	58-61	52-59	10	8	0	2	9	1	1	0	24	7	1	0	17	9	1	0
Lazarus 2021-BNT+RQV	19-27	18-23	10	5	0	0	8	4	0	0	16	7	0	0	11	0	1	0
Song 2017	373	382					27	25			75	49						
Zimmermann 2013	not reoprted																	
Izikson 2022	100	89	36	8	3	0	25	8	4	0	49	21	6	0	46	13	7	0
Schwarz 2017	409	411	57	32	5	2	89	37			135	55						
Song-2 2015	107	56					11	4			24	6			10	2		
Ortiz 2022	62	53	2	1			8	4			28	7			19	6		
Weston 2012	726	707	121	101	16	11	137	87	10	10	266	193	27	15				

Name	n-con	n-seq	Gastro-total		Gastro-grade 3+		Arthralgia-total		Arthralgia-grade 3+		Muscle pain-total		Muscle pain-grade 3+		Malaise-total		Malaise-grade 3+	
			re-con	re-seq	re-con	re-seq	re-con	re-seq	re-con	re-seq	re-con	re-seq	re-con	re-seq	re-con	re-seq	re-con	re-seq
Weston 2009	110	108	18	12	2	1	14	13	0	0	26	18	2	0				
Wang 2022	240	240	1	3							2	1						
Herbinger 2014	196	195	8	21			10	27			39	62			25	57		
Frenck 2012	261-399	259-382	95	68			102	73			252	123						

n-con= Number of participants in the concomitant vaccination group, n-seq= Number of participants in the sequence vaccination group, re-con= Number of responders in the concomitant vaccination group, re-seq= Number of responders in the sequence vaccination group. The Toback 2022-aged group had only a total of 28 individuals in adverse event group and adverse event rate of seasonal influenza was low, therefore the error after data correction was higher in Toback 2022-aged group, so the 2 subgroups of the Toback 2022 group were combined and analyzed in the SIV adverse events group

eTable13. Subgroup analysis and meta-regression results of SCR in SARS-COV-2 vaccine group

SCR<br>Subgroup analysis	Risk Ratios (RRs)	groups	participants	P value
**VRIDVs type**				
recombinant vaccine	1.002 (0.966 to 1.039)	5	980	0.72
mRNA Vaccine	0.994 (0.949 to 1.042)	4	470	
inactivated vaccine	0.978 (0.935 to 1.024)	1	459	
**Concomitant administered vaccine type**				
inactivated vaccine	0.987 (0.956 to 1.018)	6	1072	0.13
recombinant vaccine	1.108 (0.987 to 1.243)	2	180	
split vaccine	0.977 (0.941 to 1.016)	2	657	
**Mean age (year)**				
≥ 65	0.979 (0.933 to 1.026)	4	541	0.45
< 65	0.999 (0.971 to 1.028)	6	1368	
**Female rate (%)**				
≥ 55	0.999 (0.965 to 1.035)	7	1222	0.55
< 55	0.985 (0.957 to 1.014)	3	687	
**Booster vaccine**				
(Exclude wang et al)				
yes	1.008 (0.965 to 1.053)	7	858	0.44
no	0.984 (0.959 to 1.011)	2	592	
**Placebo used**				
yes	0.98 (0.956 to 1.005)	4	660	0.19
no	1.019 (0.967 to 1.074)	6	1249	
**Adjuvant used**				
yes	na			
No				

Meta-regression	Coefficient (95% confidence intervals)	z-value	P value
**Intrcpt (k=10)**	-0.044 (-0.12 to 0.034)	-1.11	0.27
**Mean age**	0.0006 (-0.0009 to 0.0021)	0.81	0.42
**Female rate**	0.013 (-0.18 to 0.21)	0.13	0.89
**placebo**	0.017 (-0.027 to 0.061)	0.76	0.45
**Adjuvant**	na		
**Booster (k=9)**	-0.031 (-0.13 to 0.068)	-0.61	0.54

eTable14. Subgroup analysis and meta-regression results of GMT in SARS-COV-2 vaccine group

GMT<br>Subgroup analysis	Risk Ratios (RRs)	groups	participants	P value
**VRIDVs type**				
recombinant vaccine	0.844 (0.725 to 0.982)	5	984	< 0.0001
mRNA Vaccine	0.914 (0.852 to 0.98)	4	481	
inactivated vaccine	0.722 (0.706 to 0.738)	1	459	
**Concomitant administered vaccine type**				
inactivated vaccine	0.875 (0.767 to 0.999)	6	1084	0.85
recombinant vaccine	0.843 (0.813 to 0.875)	2	183	
split vaccine	0.834 (0.682 to 1.02)	2	657	
**Mean age (year)**				
≥ 65	0.995 (0.958 to 1.034)	4	543	< 0.0001
< 65	0.806 (0.732 to 0.888)	6	1381	
**Female rate (%)**				
≥ 55	0.853 (0.79 to 0.921)	7	1235	0.82
< 55	0.877 (0.699 to 1.101)	3	689	
**Booster vaccine**				
(Exclude wang et al)				
yes	0.925 (0.874 to 0.978)	7	873	< 0.0001
no	0.682 (0.626 to 0.743)	2	592	
**Placebo used**				
yes	0.918 (0.86 to 0.979)	4	675	0.04
no	0.765 (0.651 to 0.899)	6	1249	
**Adjuvant used**				
yes	na			
No				

Meta-regression	Coefficient (95% confidence intervals)	z-value	P value
**Intrcpt (k=10)**	-0.918 (-1.062 to -0.774)	-12.47	<0.0001
**Mean age**	0.0097 (0.0082 to 0.0113)	12.59	<0.0001
**Female rate**	0.282 (0.089 to 0.474)	2.87	0.0041
**placebo**	0.127 (0.091 to 0.163)	6.83	<0.0001
**Adjuvant**	na		
**Booster (k=9)**	0.089 (0.021 to 0.16)	0.088	0.929

eTable15. Subgroup analysis and meta-regression results of GMFR in SARS-COV-2 vaccine group

**GMFR**<br>**Subgroup analysis**	**Risk Ratios (RRs)**	**groups**	**participants**	**P value**
**VRIDVs type**				
recombinant vaccine	0.641 (0.619 to 0.663)	2	592	< 0.0001
mRNA Vaccine	0.965 (0.915 to 1.017)	1	198	
inactivated vaccine	0.726 (0.71 to 0.743)	1	459	
**Concomitant administered vaccine type**				
inactivated vaccine	0.641 (0.619 to 0.663)	2	592	0.0092
recombinant vaccine	na	0		
split vaccine	0.835 (0.686 to 1.017)	2	657	
**Mean age (year)**				
≥ 65	0.961 (0.912 to 1.013)	2	322	< 0.0001
< 65	0.683 (0.626 to 0.745)	2	927	
**Female rate (%)**	0.815 (0.676 to 0.982)			
≥ 55	0.815 (0.676 to 0.982)	3	781	0.01
< 55	0.641 (0.619 to 0.663)	1	468	
**Booster vaccine**<br>(Exclude wang et al)				
yes	0.965 (0.915 to 1.017)	1	198	< 0.0001
no	0.641 (0.619 to 0.663)	2	592	
**Placebo used**				
yes	na			
no				
**Adjuvant used**				
yes	na			
No				

**Meta-regression**	**Coefficient**<br>**(95% confidence intervals)**	**z-value**	**P value**
**Intrcpt (k=4)**	-1.1 (-1.33 to -0.83)	-8.50	<0.0001
**Mean age**	0.017 (0.011 to 0.023)	5.7	<.0001
**Female rate**	0.66 (-1.18 to 2.49)	0.7	0.48
**placebo**	na		
**Adjuvant**	na		
**Booster (k=3)**	na		

eTable16. Subgroup analysis and meta-regression results of fever in SARS-COV-2 vaccine group

**Fever**				
**Subgroup analysis**	**Risk Ratios (RRs)**	**groups**	**participants**	**P value**
**VRIDVs type**				
recombinant vaccine	1.0167 (0.6863 to 1.5062)	5	1735	0.89
mRNA Vaccine	1.0353 (0.6683 to 1.604)	4	458	
inactivated vaccine	0.6667 (0.1124 to 3.9541)	1	480	
**Concomitant administered vaccine type**				
inactivated vaccine	1.0535 (0.7394 to 1.5012)	6	1821	0.99
recombinant vaccine	0.9833 (0.2981 to 3.2433)	2	168	
split vaccine	1.0432 (0.5143 to 2.1161)	2	684	
**Mean age (year)**				
≥ 65	0.9526 (0.5746 to 1.5793)	4	655	0.77
< 65	1.0419 (0.7331 to 1.4808)	6	2018	
**Female rate (%)**				
≥ 55	0.9747 (0.6893 to 1.3783)	7	1344	0.7
< 55	1.0993 (0.652 to 1.8536)	3	1329	
**Booster vaccine** (Exclude wang et al)				
yes	0.9039 (0.6554 to 1.2465)	7	829	0.038
no	2.1582 (1.0133 to 4.5967)	2	1364	
**Placebo used**				
yes	0.8585 (0.6025 to 1.2233)	6	625	0.11
no	1.4127 (0.8495 to 2.3494)	4	2048	
**Adjuvant used**				
yes				
No				

**Meta-regression**	**Coefficient (95% confidence intervals)**	**z-value**	**P value**
**Intrcpt (k=10)**	1.07 (-1.40 to 3.55)	0.85	0.39
**Mean age**	-0.013 (-0.038 to 0.012)	-1.04	0.3
**Female rate**	0.09 (-3.55 to 3.74)	0.05	0.96
**placebo**	-0.56 (-1.27 to 0.15)	-1.55	0.12
**Adjuvant**	na		
**Booster (k=9)**	-0.92 (-2.79 to 0.95)	-0.96	0.34

eTable17. Subgroup analysis and meta-regression results of fatigue in SARS-COV-2 vaccine group

**Fatigue**<br>**Subgroup analysis**	**Risk Ratios (RRs)**	**groups**	**participants**	**P value**
**VRIDVs type**				
recombinant vaccine	1.0497 (0.8808 to 1.251)	5	1747	0.81
mRNA Vaccine	1.0763 (0.904 to 1.2814)	4	467	
inactivated vaccine	0.5 (0.0456 to 5.4774)	1	480	
**Concomitant administered vaccine type**				
inactivated vaccine	1.1572 (1.0107 to 1.3249)	6	1836	0.059
recombinant vaccine	1.2553 (0.9883 to 1.5945)	2	174	
split vaccine	0.897 (0.7336 to 1.0969)	2	684	
**Mean age (year)**				
≥ 65	0.9482 (0.8016 to 1.1215)	4	662	0.02
< 65	1.2165 (1.068 to 1.3856)	6	2032	
**Female rate (%)**				
≥ 55	1.053 (0.9361 to 1.1846)	7	1358	0.21
< 55	1.2153 (1.0061 to 1.4681)	3	1336	
**Booster vaccine** (Exclude wang et al)				
yes	1.053 (0.9438 to 1.1748)	7	850	0.1011
no	1.3226 (1.0305 to 1.6976)	2	1364	
**Placebo used**				
yes	1.116 (0.9794 to 1.2717)	6	646	0.96
no	1.1101 (0.9426 to 1.3073)	4	2048	
**Adjuvant used**				
yes				
No				

**Meta-regression**	**Coefficient (95% confidence intervals)**	**z-value**	**P value**
**Intrcpt (k=10)**	0.9905 (0.21 to 1.77)	2.49	0.013
**Mean age**	-0.0088 (-0.017 to -0.0007)	-2.14	0.033
**Female rate**	-0.84 (-2.17 to 0.49)	-1.22	0.22
**placebo**	0.099 (-0.14 to 0.34)	0.07	0.94
**Adjuvant**			
**Booster (k=9)**	0.026 (-0.76 to 0.81)	0.06	0.95

eTable18. Subgroup analysis and meta-regression results of headache in SARS-COV-2 vaccine group

**Headache**<br>**Subgroup analysis**	**Risk Ratios (RRs)**	**groups**	**participants**	**P value**
**VRIDVs type**				
recombinant vaccine	1.0497 (0.8808 to 1.251)	5	1742	0.81
mRNA Vaccine	1.0763 (0.904 to 1.2814)	4	463	
inactivated vaccine	0.5 (0.0456 to 5.4774)	1	480	
**Concomitant administered vaccine type**				
inactivated vaccine	1.0443 (0.8928 to 1.2215)	6	1832	0.42
recombinant vaccine	1.2747 (0.9187 to 1.7686)	2	169	
split vaccine	0.9647 (0.7388 to 1.2596)	2	684	
**Mean age (year)**				
≥ 65	1.0089 (0.812 to 1.2536)	4	659	0.61
< 65	1.0803 (0.9266 to 1.2594)	6	2026	
**Female rate (%)**				
≥ 55	1.1222 (0.9674 to 1.3018)	7	1352	0.27
< 55	0.9683 (0.7777 to 1.2058)	3	1333	
**Booster vaccine**<br>(Exclude wang et al)				
yes	1.106 (0.961 to 1.2728)	7	841	0.36
no	0.9644 (0.7447 to 1.2489)	2	1364	
**Placebo used**				
yes	1.1542 (0.9784 to 1.3617)	6	637	0.16
no	0.9645 (0.798 to 1.1656)	4	2048	
**Adjuvant used**				
yes				
No				

**Meta-regression**	**Coefficient (95% confidence intervals)**	**z-value**	**P value**
**Intrcpt (k=10)**	-0.14 (-1.02 to 0.75)	-0.3	0.76
**Mean age**	-0.002 (-0.012 to 0.009)	-0.29	0.76
**Female rate**	0.37 (-1.27 to 2.01)	0.45	0.66
**placebo**	0.14 (-0.17 to 0.45)	0.89	0.37
**Adjuvant**	na		
**Booster (k=9)**	0.08 (-0.70 to 0.86)	0.19	0.84

eTable19. Subgroup analysis and meta-regression results of malaise in SARS-COV-2 vaccine group

Malaise				
**Subgroup analysis**	**Risk Ratios (RRs)**	**groups**	**participants**	**P value**
**VRIDVs type**				
recombinant vaccine	1.1025 (0.8696 to 1.3978)	5	1741	0.62
mRNA Vaccine	1.0155 (0.814 to 1.267)	4	459	
inactivated vaccine	na		0	
**Concomitant administered vaccine type**				
inactivated vaccine	1.1413 (0.9147 to 1.4239)	6	1827	
recombinant vaccine	1.2305 (0.7715 to 1.9626)	2	169	
split vaccine	0.8393 (0.6376 to 1.1048)	1	204	
**Mean age (year)**				
≥ 65	0.8033 (0.6253 to 1.032)	4	655	0.007
< 65	1.2658 (1.0193 to 1.5718)	5	1545	
**Female rate (%)**				
≥ 55	1.0454 (0.8627 to 1.2666)	6	871	0.8
< 55	1.0924 (0.8067 to 1.4794)	3	1329	
**Booster vaccine**				
yes	1.0079 (0.8409 to 1.2082)	7	836	0.3026
no	1.2507 (0.8655 to 1.8071)	2	1364	
**Placebo used**				
yes	1.1142 (0.8788 to 1.4126)	6	632	0.56
no	1.0124 (0.8084 to 1.2679)	3	1568	
**Adjuvant used**				
yes				
No				

Meta-regression	Coefficient (95% confidence intervals)	z-value	P value
**Intrcpt (k=9)**	2.12 (-0.49 to 4.74)	1.59	0.11
**Mean age**	-0.035 (-0.068 to -0.0012)	-2.03	0.043
**Female rate**	-1.27 (-4.97 to 2.42)	-0.68	0.5
**Booster**	0.88 (-0.64 to 2.39)	1.13	0.26
**Placebo**	-0.26 (-0.87 to 0.36)	-0.81	0.42
**Adjuvant**	na		

eTable20. Subgroup analysis and meta-regression results of muscle pain in SARS-COV-2 vaccine group

**Muscle pain**<br>**Subgroup analysis**	**Risk Ratios (RRs)**	**groups**	**participants**	**P value**
**VRIDVs type**				
recombinant vaccine	1.2992 (1.0799 to 1.5632)	5	1740	0.18
mRNA Vaccine	1.0203 (0.843 to 1.2349)	4	460	
inactivated vaccine	2 (0.1826 to 21.9096)	1	480	
**Concomitant administered vaccine type**				
inactivated vaccine	1.3274 (1.114 to 1.5818)	6	1829	0.007
recombinant vaccine	1.2373 (0.8693 to 1.7611)	2	167	
split vaccine	0.8151 (0.634 to 1.048)	2	684	
**Mean age (year)**				
≥ 65	0.9421 (0.7497 to 1.1839)	4	657	0.02
< 65	1.313 (1.1126 to 1.5494)	6	2023	
**Female rate (%)**				
≥ 55	1.0959 (0.9275 to 1.2949)	7	1349	0.21
< 55	1.3044 (1.0464 to 1.6261)	3	1331	
**Booster vaccine** (Exclude wang et al)				
yes	1.1469 (0.9781 to 1.3449)	7	836	0.6233
no	1.2339 (0.9662 to 1.5785)	2	1364	
**Placebo used**				
yes	1.3734 (1.1186 to 1.6862)	6	632	0.048
no	1.0459 (0.8762 to 1.2485)	4	2048	
**Adjuvant used**				
yes				
No				

**Meta-regression**	**Coefficient (95% confidence intervals)**	**z-value**	**P value**
**Intrcpt (k=10)**	0.83 (-0.08 to 1.75)	1.77	0.076
**Mean age**	-0.01 (-0.021 to 0.001)	-1.75	0.079
**Female rate**	-0.58 (-2.64 to 1.47)	-0.55	0.58
**placebo**	0.356 (0.0018 to 0.709)	1.97	0.049
**Adjuvant**	na		
**Booster (k=9)**	-0.43 (-1.49 to 0.62)	-0.81	0.42

eTable21. Subgroup analysis and meta-regression results of gastrointestinal symptom in SARS-COV-2 vaccine group

**gastrointestinal symptom Subgroup analysis**	**Risk Ratios (RRs)**	**groups**	**participants**	**P value**
**VRIDVs type**				
recombinant vaccine	1.0799 (0.7765 to 1.5018)	5	1731	0.44
mRNA Vaccine	1.4064 (1.0388 to 1.904)	4	455	
inactivated vaccine	3 (0.1228 to 73.2762)	1	480	
**Concomitant administered vaccine type**				
inactivated vaccine	1.372 (1.0052 to 1.8725)	6	1818	0.2
recombinant vaccine	0.8118 (0.4842 to 1.3611)	2	164	
split vaccine	1.3669 (0.9092 to 2.0549)	2	684	
**Mean age (year)**				
≥ 65	1.1222 (0.7832 to 1.608)	4	654	0.48
< 65	1.3239 (0.9973 to 1.7575)	6	2012	
**Female rate (%)**				
≥ 55	1.3546 (1.0554 to 1.7385)	7	1338	0.21
< 55	0.9532 (0.5844 to 1.555)	3	1328	
**Booster vaccine** (Exclude wang et al)				
yes	1.2695 (1.0003 to 1.611)	7	822	0.6193
no	1.0727 (0.5771 to 1.9941)	2	1364	
**Placebo used**				
yes	1.2396 (0.9255 to 1.6603)	6	618	0.96
no	1.252 (0.8885 to 1.7643)	4	2048	
**Adjuvant used**				
yes				
No				

**Meta-regression**	**Coefficient (95% confidence intervals)**	**z-value**	**P value**
**Intrcpt (k=10)**	-1.1 (-3.8 to 1.59)	-0.8	0.42
**Mean age**	-0.015 (-0.04 to 0.013)	-1.04	0.29
**Female rate**	4.15 (-0.23 to 8.54)	1.85	0.063
**placebo**	-0.39 (-1.17 to 0.39)	-0.99	0.32
**Adjuvant**	na		
**Booster (k=9)**	0.66 (-1.53 to 2.85)	0.59	0.55

eTable22. Subgroup analysis and meta-regression results of chills in SARS-COV-2 vaccine group

**Chills**				
**Subgroup analysis**	**Risk Ratios (RRs)**	**groups**	**participants**	**P value**
**VRIDVs type**				
recombinant vaccine	0.8227 (0.5114 to 1.3233)	3	368	0.075
mRNA Vaccine	1.3922 (1.0011 to 1.9361)	4	458	
inactivated vaccine	na		0	
**Concomitant administered vaccine type**				
inactivated vaccine	1.0118 (0.6992 to 1.464)	4	456	0.22
recombinant vaccine	0.8981 (0.4297 to 1.8771)	2	166	
split vaccine	1.6343 (1.0181 to 2.6234)	1	204	
**Mean age (year)**				
≥ 65	1.2968 (0.8815 to 1.9078)	3	410	0.42
< 65	1.0388 (0.7127 to 1.514)	4	416	
**Female rate (%)**				
≥ 55	1.2358 (0.922 to 1.6565)	5	620	0.31
< 55	0.8401 (0.4214 to 1.6751)	2	206	
**Booster vaccine**				
yes	na			
no				
**Placebo used**				
yes	0.9856 (0.7081 to 1.3717)	6	622	0.086
no	1.6343 (1.0181 to 2.6234)	1	204	
**Adjuvant used**				
yes				
No				

**Meta-regression**	**Coefficient (95% confidence intervals)**	**z-value**	**P value**
**Intrcpt**	2.28 (-5.53 to 10.1)	0.57	0.57
**Mean age**	-0.022 (-0.085 to 0.04)	-0.69	0.49
**Female rate**	-0.41 (-7.01 to 6.2)	-0.12	0.9
**Booster**	na		
**Placebo**	-0.86 (-1.9 to 0.14)	-1.68	0.094
**Adjuvant**	na		

eTable23. Subgroup analysis and meta-regression results of local pain in SARS-COV-2 vaccine group

**local pain**<br>**Subgroup analysis**	**Risk Ratios (RRs)**	**groups**	**participants**	**P value**
**VRIDVs type**				
recombinant vaccine	1.0813 (0.9571 to 1.2217)	5	1753	0.58
mRNA Vaccine	1.0028 (0.9252 to 1.0869)	4	473	
inactivated vaccine	0.9643 (0.5864 to 1.5858)	1	480	
**Co-administered vaccine type**				
inactivated vaccine	1.0871 (0.9803 to 1.2056)	6	1848	0.33
recombinant vaccine	0.957 (0.8075 to 1.1342)	2	174	
split vaccine	0.9763 (0.8366 to 1.1393)	2	684	
**Mean age (year)**				
≥ 65	0.946 (0.8341 to 1.0729)	4	668	0.086
< 65	1.0876 (0.9863 to 1.1993)	6	2038	
**Female rate (%)**				
≥ 55	0.9968 (0.916 to 1.0847)	7	1364	0.2
< 55	1.1156 (0.9593 to 1.2975)	3	1342	
**Booster vaccine**<br>(Exclude wang et al)				
yes	0.978 (0.9056 to 1.0562)	7	862	0.022
no	1.2435 (1.0283 to 1.5037)	2	1364	
**Placebo used**				
yes	0.9772 (0.8891 to 1.0741)	6	658	0.12
no	1.107 (0.9787 to 1.2522)	4	2048	
**Adjuvant used**				
yes				
No				

**Meta-regression**	**Coefficient (95% confidence intervals)**	**z-value**	**P value**
**Intrcpt (k=10)**	0.44 (-0.089 to 0.97)	1.63	0.10
**Mean age**	-0.007 (-0.013 to -0.0013)	-2.4	0.016
**Female rate**	0.057 (-0.77 to 0.88)	0.14	0.89
**placebo**	-0.11 (-0.28 to 0.058)	-1.29	0.19
**Adjuvant**	na		
**Booster (k=9)**	-0.069 (-0.57 to 0.44)	-0.27	0.79

eTable24. Subgroup analysis and meta-regression results of erythema in SARS-COV-2 vaccine group

Erythema				
**Subgroup analysis**	**Risk Ratios (RRs)**	**groups**	**participants**	**P value**
**VRIDVs type**				
recombinant vaccine	1.0395 (0.6959 to 1.5527)	5	1735	0.42
mRNA Vaccine	0.8232 (0.5817 to 1.1649)	4	455	
inactivated vaccine	0.4 (0.0784 to 2.0416)	1	480	
**Concomitant administered vaccine type**				
inactivated vaccine	1.0774 (0.7478 to 1.5524)	6	1820	0.3
recombinant vaccine	0.7842 (0.4148 to 1.4826)	2	166	
split vaccine	0.6903 (0.4381 to 1.0876)	2	684	
**Mean age (year)**				
≥ 65	0.8414 (0.5688 to 1.2446)	4	657	0.62
< 65	0.9593 (0.6779 to 1.3576)	6	2013	
**Female rate (%)**				
≥ 55	0.8906 (0.6722 to 1.1799)	7	1339	0.96
< 55	0.8772 (0.4613 to 1.6679)	3	1331	
**Booster vaccine** (Exclude wang et al)				
yes	0.9409 (0.7204 to 1.2289)	7	826	0.48
no	0.5882 (0.1631 to 2.1211)	2	1364	
**Placebo used**				
yes	1.0498 (0.7584 to 1.4531)	6	622	0.11
no	0.673 (0.4364 to 1.0377)	4	2048	
**Adjuvant used**				
yes				
No				

**Meta-regression**	**Coefficient (95% confidence intervals)**	**z-value**	**P value**
**Intrcpt (k=10)**	-2.31 (-5.81 to 1.19)	-1.29	0.19
**Mean age**	0.015 (-0.015 to 0.045)	1	0.32
**Female rate**	1.67 (-2.36 to 5.71)	0.81	0.42
**placebo**	0.53 (-0.11 to 1.16)	1.63	0.1
**Adjuvant**	na		
**Booster (k=9)**	-0.89 (-2.99 to 1.21)	-0.83	0.41

eTable25. Subgroup analysis and meta-regression results of swelling in SARS-COV-2 vaccine group

**Swelling**<br>**Subgroup analysis**	**Risk Ratios (RRs)**	**groups**	**participants**	**P value**
**VRIDVs type**				
recombinant vaccine	0.7781 (0.4884 to 1.2396)	4	1734	0.48
mRNA Vaccine	0.6789 (0.4583 to 1.0056)	4	454	
inactivated vaccine	0.3333 (0.0914 to 1.2162)	1	480	
**Concomitant administered vaccine type**				
inactivated vaccine	0.852 (0.5674 to 1.2794)	5	1819	0.26
recombinant vaccine	0.664 (0.3279 to 1.3449)	2	165	
split vaccine	0.487 (0.2857 to 0.83)	2	684	
**Mean age (year)**				
≥ 65	0.7277 (0.4567 to 1.1596)	3	654	0.73
< 65	0.6556 (0.4507 to 0.9534)	6	2014	
**Female rate (%)**				
≥ 55	0.6046 (0.4379 to 0.8346)	6	1340	0.09
< 55	1.19 (0.5799 to 2.442)	3	1328	
**Booster vaccine** (Exclude wang et al)				
yes	0.7088 (0.5218 to 0.9629)	7	824	0.6801
no	0.98 (0.2165 to 4.4368)	1	1121	
**Placebo used**				
yes	0.7894 (0.5499 to 1.1332)	6	620	0.2
no	0.53 (0.3216 to 0.8736)	3	2048	
**Adjuvant used**				
yes				
No				

**Meta-regression**	**Coefficient (95% confidence intervals)**	**z-value**	**P value**
**Intrcpt (k=9)**	-0.35 (-4.09 to 3.39)	-0.18	0.85
**Mean age**	0.0068 (-0.025 to 0.038)	0.42	0.67
**Female rate**	-1.23 (-5.76 to 3.3)	-0.53	0.59
**placebo**	0.49 (-0.22 to 1.21)	-0.53	0.59
**Adjuvant**	na		
**Booster (k=8)**	-2.18 (-5.37 to 1)	-1.34	0.18

eTable26. Subgroup analysis and meta-regression results of Induration in SARS-COV-2 vaccine group

Induration				
**Subgroup analysis**	**Risk Ratios (RRs)**	**groups**	**participants**	**P value**
**VRIDVs type**				
recombinant vaccine	0.881 (0.5784 to 1.3418)	3	370	0.18
mRNA Vaccine	0.5835 (0.4 to 0.8511)	4	453	
inactivated vaccine	1.5 (0.4287 to 5.2483)	1	480	
**Concomitant administered vaccine type**				
inactivated vaccine	0.7672 (0.5426 to 1.0849)	4	455	0.77
recombinant vaccine	0.5737 (0.2789 to 1.1802)	2	164	
split vaccine	0.7531 (0.4332 to 1.3091)	2	684	
**Mean age (year)**				
≥ 65	0.5709 (0.3642 to 0.895)	3	414	0.16
< 65	0.8563 (0.6072 to 1.2077)	5	889	
**Female rate (%)**				
≥ 55	0.7886 (0.5836 to 1.0658)	6	1093	0.27
< 55	0.529 (0.2777 to 1.0075)	2	210	
**Booster vaccine** (Exclude wang et al)				
yes	na			
no				
**Placebo used**				
yes	0.7222 (0.5285 to 0.9868)	6	619	0.89
no	0.7531 (0.4332 to 1.3091)	2	684	
**Adjuvant used**				
yes				
No				

Meta-regression	Coefficient (95% confidence intervals)	z-value	P value
**Intrcpt (k=8)**	-1.43 (-8.02 to 5.16)	-0.43	0.67
**Mean age**	0.0041 (-0.054 to 0.045)	-0.16	0.87
**Female rate**	2.42 (-4.03 to 8.87)	0.74	0.46
**placebo**	-0.12 (-0.95 to 0.72)	-0.27	0.78
**Adjuvant**	na		
**Booster (k=7)**	na		

eTable27. Subgroup analysis and meta-regression results of A/H1N1-SCR in seasonal influenza vaccine group

A/H1N1-SCR				
**Subgroup analysis**	**Risk Ratios (RRs)**	**groups**	**participants**	**P value**
**VRIDV type**				
split vaccine	1.017 (0.974 to 1.061)	12	7156	0.85
recombinant vaccine	0.942 (0.715 to 1.243)	2	182	
inactivated vaccine	1.021 (0.953 to 1.094)	9	2203	
**Concomitant administered vaccine type**				
polysaccharide vaccine	1.02 (0.95 to 1.096)	5	5017	0.75
attenuated live vaccine	0.968 (0.867 to 1.081)	2	1540	
recombinant vaccine	1.02 (0.943 to 1.103)	7	1778	
mRNA Vaccine	1.12 (0.936 to 1.341)	4	455	
inactivated vaccine	1.018 (0.963 to 1.076)	5	3452	
**Mean age (year)**				
≥ 65	1.04 (0.98 to 1.104)	10	4093	0.31
< 65	1.001 (0.956 to 1.047)	13	5448	
**Female rate (%)**				
≥ 55	1.009 (0.962 to 1.059)	15	6105	0.63
< 55	1.027 (0.972 to 1.085)	8	3436	
**Booster vaccine**				
yes	na			
no				
**Placebo used**				
yes	1.017 (0.964 to 1.073)	15	5470	0.96
no	1.015 (0.968 to 1.065)	8	4071	
**Adjuvant used**				
yes	1.029 (0.921 to 1.15)	5	1168	0.8
no	1.015 (0.977 to 1.054)	18	8373	

Meta-regression	Coefficient (95% confidence intervals)	z-value	P value
**Intrcpt**	0.067 (-0.38 to 0.51)	0.29	0.77
**Mean age**	-0.0001 (-0.0032 to 0.003)	-0.06	0.95
**Female rate**	-0.069 (-0.69 to 0.55)	-0.22	0.83
**Booster**	na		
**Placebo**	-0.0035 (-0.16 to 0.15)	-0.05	0.96
**Adjuvant**	-0.023 (-0.11 to 0.06)	-0.54	0.59

eTable28. Subgroup analysis and meta-regression results of A/H1N1-SPR in seasonal influenza vaccine group

**A/H1N1-SPR**				
**Subgroup analysis**	**Risk Ratios (RRs)**	**groups**	**participants**	**P value**
**VRIDV type**				
split vaccine	1.003 (0.99 to 1.016)	13	8311	0.039
recombinant vaccine	1.077 (0.925 to 1.255)	1	158	
inactivated vaccine	0.961 (0.93 to 0.993)	3	1288	
**Concomitant administered vaccine type**				
polysaccharide vaccine	0.999 (0.98 to 1.019)	7	4503	0.62
attenuated live vaccine	0.98 (0.952 to 1.007)	2	1540	
recombinant vaccine	1.001 (0.957 to 1.046)	2	952	
mRNA Vaccine	0.994 (0.927 to 1.066)	1	192	
inactivated vaccine	1.007 (0.988 to 1.027)	5	2570	
**Mean age (year)**				
≥ 65	0.994 (0.978 to 1.011)	9	5112	0.51
< 65	1.003 (0.984 to 1.021)	8	4645	
**Female rate (%)**				
≥ 55	0.998 (0.980 to 1.015)	10	5675	0.89
< 55	0.999 (0.983 to 1.015)	7	4082	
**Booster vaccine**				
yes	na			
no				
**Placebo used**				
yes	1.002 (0.986 to 1.018)	8	5511	0.51
no	0.994 (0.975 to 1.012)	9	4246	
**Adjuvant used**				
yes	0.946 (0.901 to 0.992)	2	918	0.018
no	1.004 (0.991 to 1.016)	15	8839	

**Meta-regression**	**Coefficient (95% confidence intervals)**	**z-value**	**P value**
**Intrcpt**	0.14 (-0.028 to 0.3)	1.62	0.1
**Mean age**	-0.0006 (-0.0015 to 0.0002)	-1.42	0.15
**Female rate**	-0.17 (-0.41 to 0.078)	-1.34	0.18
**Booster**			
**Placebo**	-0.027 (-0.094 to 0.04)	-0.79	0.43
**Adjuvant**	-0.0083 (-0.035 to 0.019)	-0.60	0.55

eTable29. Subgroup analysis and meta-regression results of A/H1N1-GMT in seasonal influenza vaccine group

**A/H1N1-GMT**				
**Subgroup analysis**	**Risk Ratios (RRs)**	**groups**	**participants**	**P value**
**VRIDV type**				
split vaccine	1.039 (0.977 to 1.103)	14	9911	0.98
recombinant vaccine	1.012 (0.715 to 1.432)	2	184	
inactivated vaccine	1.03 (0.922 to 1.15)	9	2152	
**Concomitant administered vaccine type**				
polysaccharide vaccine	1.03 (0.918 to 1.156)	6	5021	0.57
attenuated live vaccine	0.973 (0.864 to 1.096)	2	1540	
recombinant vaccine	1.047 (0.939 to 1.168)	7	1779	
mRNA Vaccine	1.092 (1.009 to 1.182)	4	455	
inactivated vaccine	1.005 (0.869 to 1.162)	6	3452	
**Mean age (year)**				
≥ 65	1.054 (0.972 to 1.144)	11	5721	0.53
< 65	1.018 (0.947 to 1.095)	14	6526	
**Female rate (%)**				
≥ 55	1.023 (0.946 to 1.107)	17	8071	0.54
< 55	1.052 (1.008 to 1.098)	8	4176	
**Booster vaccine**				
yes	na			
no				
**Placebo used**				
yes	1.038 (0.976 to 1.103)	16	7339	0.81
no	1.023 (0.919 to 1.138)	9	4908	
**Adjuvant used**				
yes	1.078 (0.898 to 1.294)	5	1117	0.59
no	1.024 (0.969 to 1.082)	20	11130	

**Meta-regression**	**Coefficient (95% confidence intervals)**	**z-value**	**P value**
**Intrcpt**	0.034 (-0.51 to 0.57)	0.12	0.9
**Mean age**	-0.0002 (-0.0048 to 0.0044)	-0.10	0.92
**Female rate**	-0.013 (-0.77 to 0.75)	-0.03	0.97
**Booster**	na		
**Placebo**	0.056 (-0.096 to 0.21)	0.72	0.47
**Adjuvant**	0.017 (-0.092 to 0.13)	0.3	0.76

eTable30. Subgroup analysis and meta-regression results of A/H3N2-SCR in seasonal influenza vaccine group

**A/H3N2-SCR**				
**Subgroup analysis**	**Risk Ratios (RRs)**	**groups**	**participants**	**P value**
**VRIDV type**				
split vaccine	1.016 (0.98 to 1.054)	12	7156	0.17
recombinant vaccine	1.172 (0.94 to 1.462)	2	182	
inactivated vaccine	1.082 (1.003 to 1.166)	9	2201	
**Concomitant administered vaccine type**				
polysaccharide vaccine	1.075 (0.982 to 1.177)	5	3237	0.67
attenuated live vaccine	1.011 (0.956 to 1.07)	2	1510	
recombinant vaccine	1.069 (0.968 to 1.181)	7	1775	
mRNA Vaccine	0.989 (0.792 to 1.236)	4	454	
inactivated vaccine	1.013 (0.972 to 1.056)	5	2563	
**Mean age (year)**				
≥ 65	1.063 (1.007 to 1.122)	10	4092	0.17
< 65	1.014 (0.973 to 1.056)	13	5447	
**Female rate (%)**				
≥ 55	1.026 (0.985 to 1.069)	15	6104	0.57
< 55	1.046 (0.991 to 1.105)	8	3435	
**Booster vaccine**				
yes	na			
no				
**Placebo used**				
yes	1.013 (0.966 to 1.062)	15	5468	0.21
no	1.056 (1.01 to 1.104)	8	4071	
**Adjuvant used**				
yes	1.193 (1.055 to 1.349)	5	1167	0.015
no	1.018 (0.984 to 1.053)	18	8372	

**Meta-regression**	**Coefficient (95% confidence intervals)**	**z-value**	**P value**
**Intrcpt**	0.074 (-0.35 to 0.51)	0.34	0.73
**Mean age**	0.002 (-0.0005 to 0.0044)	1.56	0.12
**Female rate**	-0.28 (-0.85 to 0.28)	-0.98	0.33
**Booster**	na		
**Placebo**	0.17 (0.024 to 0.31)	2.29	0.022
**Adjuvant**	-0.019 (-0.076 to 0.038)	-0.64	0.52

eTable31. Subgroup analysis and meta-regression results of A/H3N2-SPR in seasonal influenza vaccine group

A/H3N2-SPR<br>Subgroup analysis	Risk Ratios (RRs)	groups	participants	P value
**VRIDV type**				
split vaccine	1 (0.987 to 1.013)	13	8306	0.401
recombinant vaccine	0.923 (0.809 to 1.053)	1	158	
inactivated vaccine	0.994 (0.981 to 1.006)	3	1288	
**Concomitant administered vaccine type**				
polysaccharide vaccine	0.994 (0.977 to 1.012)	7	4498	0.85
attenuated live vaccine	1.006 (0.976 to 1.037)	2	1540	
recombinant vaccine	1.017 (0.95 to 1.088)	2	952	
mRNA Vaccine	0.971 (0.928 to 1.016)	1	192	
inactivated vaccine	0.997 (0.986 to 1.008)	5	2570	
**Mean age (year)**				
≥ 65	0.997 (0.986 to 1.008)	9	5107	0.82
< 65	1 (0.978 to 1.021)	8	4645	
**Female rate (%)**				
≥ 55	1.001 (0.986 to 1.016)	10	5675	0.53
< 55	0.994 (0.976 to 1.012)	7	4077	
**Booster vaccine**				
yes	na			
no				
**Placebo used**				
yes	1 (0.984 to 1.017)	8	5506	0.63
no	0.995 (0.98 to 1.01)	9	4246	
**Adjuvant used**				
yes	0.993 (0.979 to 1.007)	2	918	0.55
no	0.999 (0.986 to 1.011)	15	8834	

Meta-regression	Coefficient (95% confidence intervals)	z-value	P value
**Intrcpt**	-0.11 (-0.29 to 0.073)	-1.18	0.24
**Mean age**	0.0004 (-0.0005 to 0.0013)	0.9	0.37
**Female rate**	0.14 (-0.094 to 0.37)	1.17	0.24
**Booster**	na		
**Placebo**	-0.015 (-0.047 to 0.017)	-0.92	0.36
**Adjuvant**	0.01 (-0.0048 to 0.025)	1.33	0.18

eTable32. Subgroup analysis and meta-regression results of A/H3N2-GMT in seasonal influenza vaccine group

**A/H3N2-GMT**				
**Subgroup analysis**	**Risk Ratios (RRs)**	**groups**	**participants**	**P value**
**VRIDV type**				
split vaccine	1.02 (0.954 to 1.091)	14	9907	0.52
recombinant vaccine	1.052 (1.021 to 1.084)	2	184	
inactivated vaccine	0.981 (0.838 to 1.149)	9	2151	
**Concomitant administered vaccine type**				
polysaccharide vaccine	0.971 (0.799 to 1.18)	6	3237	0.27
attenuated live vaccine	1.091 (1.075 to 1.108)	2	1510	
recombinant vaccine	1.049 (0.976 to 1.127)	7	1777	
mRNA Vaccine	1.052 (0.944 to 1.171)	4	454	
inactivated vaccine	0.96 (0.818 to 1.128)	6	2563	
**Mean age (year)**				
≥ 65	0.993 (0.875 to 1.126)	11	5717	0.67
< 65	1.023 (0.955 to 1.095)	14	6525	
**Female rate (%)**				
≥ 55	1.022 (0.936 to 1.116)	17	8070	0.577
< 55	0.986 (0.902 to 1.078)	8	4172	
**Booster vaccine**				
yes	na			
no				
**Placebo used**				
yes	1.021 (0.946 to 1.102)	16	7334	0.69
no	0.992 (0.875 to 1.125)	9	4908	
**Adjuvant used**				
yes	0.989 (0.773 to 1.265)	5	1117	0.85
no	1.013 (0.952 to 1.078)	20	11125	

**Meta-regression**	**Coefficient (95% confidence intervals)**	**z-value**	**P value**
**Intrcpt**	-0.13 (-0.79 to 0.53)	-0.39	0.7
**Mean age**	0.0009 (-0.0047 to 0.0065)	0.32	0.75
**Female rate**	0.13 (-0.8 to 1.05)	0.27	0.79
**Booster**			
**Placebo**	-0.024 (-0.21 to 0.16)	-0.25	0.8
**Adjuvant**	0.031 (-0.1 to 0.16)	0.45	0.65

eTable33. Subgroup analysis and meta-regression results of B-SCR in seasonal influenza vaccine group

**B-SCR**<br>**Subgroup analysis**	**Risk Ratios (RRs)**	**groups**	**participants**	**P value**
**VRIDV type**				
split vaccine	0.972 (0.934 to 1.012)	21	12013	0.014
recombinant vaccine	1.279 (0.942 to 1.738)	4	364	
inactivated vaccine	1.116 (1.005 to 1.239)	12	2866	
**Concomitant administered vaccine type**				
polysaccharide vaccine	1.022 (0.939 to 1.112)	8	5556	0.245
attenuated live vaccine	0.958 (0.875 to 1.049)	3	2311	
recombinant vaccine	0.998 (0.907 to 1.1)	12	3381	
mRNA Vaccine	1.311 (0.991 to 1.735)	7	829	
inactivated vaccine	0.973 (0.929 to 1.019)	7	3166	
**Mean age (year)**				
≥ 65	1.031 (0.966 to 1.101)	14	4468	0.188
< 65	0.978 (0.935 to 1.022)	23	10775	
**Female rate (%)**				
≥ 55	1.004 (0.959 to 1.052)	24	11142	0.535
< 55	0.979 (0.921 to 1.043)	13	4101	
**Booster vaccine**				
yes	na			
no				
**Placebo used**				
yes	0.987 (0.934 to 1.042)	25	9609	0.62
no	1.005 (0.957 to 1.056)	12	5634	
**Adjuvant used**				
yes	1.35 (1.08 to 1.689)	5	1168	0.006
no	0.983 (0.947 to 1.02)	32	14075	

**Meta-regression**	**Coefficient**<br>**(95% confidence intervals)**	**z-value**	**P value**
**Intrcpt**	-0.31 (-0.89 to 0.28)	-1.02	0.31
**Mean age**	0.0004 (-0.0033 to 0.004)	0.19	0.85
**Female rate**	0.43 (-0.36 to 1.2)	1.08	0.28
**Booster**	na		
**Placebo**	0.3 (0.039 to 0.55)	2.26	0.024
**Adjuvant**	0.015 (-0.064 to 0.094)	0.38	0.7

eTable34. Subgroup analysis and meta-regression results of B-SPR in seasonal influenza vaccine group

**B-SPR**				
**Subgroup analysis**	**Risk Ratios (RRs)**	**groups**	**participants**	**P value**
**VRIDV type**				
split vaccine	1.005 (0.987 to 1.022)	22	13203	0.19
recombinant vaccine	0.838 (0.688 to 1.022)	2	316	
inactivated vaccine	0.993 (0.932 to 1.057)	3	1288	
**Concomitant administered vaccine type**				
polysaccharide vaccine	1.008 (0.976 to 1.042)	11	6992	0.07
attenuated live vaccine	1.03 (0.976 to 1.087)	3	2354	
recombinant vaccine	1.004 (0.98 to 1.028)	4	1904	
mRNA Vaccine	1.006 (0.983 to 1.03)	2	384	
inactivated vaccine	0.97 (0.949 to 0.992)	7	3173	
**Mean age (year)**				
≥ 65	1.004 (0.981 to 1.027)	14	6824	0.74
< 65	0.998 (0.974 to 1.022)	13	7983	
**Female rate (%)**				
≥ 55	1.016 (0.988 to 1.045)	15	9125	0.045
< 55	0.983 (0.968 to 0.998)	12	5682	
**Booster vaccine**				
yes	na			
no				
**Placebo used**				
yes	1.018 (0.99 to 1.048)	13	8830	0.029
no	0.982 (0.966 to 0.998)	14	5977	
**Adjuvant used**				
yes	1.009 (0.932 to 1.091)	2	918	0.84
no	1 (0.983 to 1.018)	25	13889	

**Meta-regression**	**Coefficient (95% confidence intervals)**	**z-value**	**P value**
**Intrcpt**	-0.18 (-0.4 to 0.035)	-1.64	0.1
**Mean age**	0.0013 (-0.0002 to 0.0028)	1.66	0.096
**Female rate**	0.18 (-0.079 to 0.45)	1.37	0.17
**Booster**	na		
**Placebo**	-0.017 (-0.11 to 0.072)	-0.38	0.71
**Adjuvant**	0.012 (-0.014 to 0.038)	0.93	0.35

eTable35. Subgroup analysis and meta-regression results of B-GMT in seasonal influenza vaccine group

B-GMT<br>Subgroup analysis	Risk Ratios (RRs)	groups	participants	P value
**VRIDV type**				
split vaccine	0.972 (0.944 to 1.002)	22	14428	< 0.0001
recombinant vaccine	1.088 (1.046 to 1.131)	4	368	
inactivated vaccine	0.994 (0.966 to 1.022)	12	2817	
**Concomitant administered vaccine type**				
polysaccharide vaccine	0.992 (0.948 to 1.037)	8	7021	0.17
attenuated live vaccine	0.994 (0.9 to 1.097)	3	2354	
recombinant vaccine	1.009 (0.976 to 1.043)	12	3386	
mRNA Vaccine	1.009 (0.957 to 1.063)	7	832	
inactivated vaccine	0.945 (0.905 to 0.986)	8	4021	
**Mean age (year)**				
≥ 65	1.001 (0.973 to 1.03)	14	6934	0.39
< 65	0.983 (0.953 to 1.015)	24	10680	
**Female rate (%)**				
≥ 55	0.996 (0.969 to 1.022)	26	11969	0.4
< 55	0.974 (0.932 to 1.018)	12	5645	
**Booster vaccine**				
yes	na			
no				
**Placebo used**				
yes	1.01 (0.982 to 1.038)	25	11176	0.0059
no	0.957 (0.933 to 0.982)	13	6437	
**Adjuvant used**				
yes	1.017 (0.963 to 1.074)	5	1117	0.31
no	0.986 (0.963 to 1.01)	33	16497	

Meta-regression	Coefficient<br>(95% confidence intervals)	z-value	P value
**Intrcpt**	-0.078 (-0.29 to 0.13)	-0.73	0.46
**Mean age**	0.0007 (-0.0011 to 0.0026)	0.78	0.43
**Female rate**	-0.032 (-0.33 to 0.27)	-0.21	0.84
**Booster**			
**Placebo**	0.031 (-0.038 to 0.1)	0.88	0.38
**Adjuvant**	0.06 (0.018 to 0.1)	2.76	0.0058

eTable36. Subgroup analysis and meta-regression results of fever in seasonal influenza vaccine group

Fever<br>Subgroup analysis	Risk Ratios (RRs)	groups	participants	P value
**VRIDV type**				
split vaccine	2.2199 (1.2468 to 3.9526)	10	5168	0.4
recombinant vaccine	0.6414 (0.1102 to 3.7325)	1	155	
inactivated vaccine	2.3341 (0.9453 to 5.7628)	6	1648	
**Concomitant administered vaccine type**				
polysaccharide vaccine	1.5672 (0.9155 to 2.6828)	6	2806	0.0006
attenuated live vaccine	na	0	0	
recombinant vaccine	3.5815 (2.2736 to 5.6416)	5	1632	
mRNA Vaccine	1.4434 (0.0215 to 96.9733)	2	287	
inactivated vaccine	1.0592 (0.7494 to 1.4972)	4	2246	
**Mean age (year)**				
≥ 65	1.707 (0.9172 to 3.1769)	9	4212	0.27
< 65	2.6576 (1.6269 to 4.3411)	8	2759	
**Female rate (%)**				
≥ 55	1.8702 (0.9257 to 3.7785)	8	2518	0.73
< 55	2.1964 (1.1857 to 4.0687)	9	4629	
**Booster vaccine**				
yes				
no				
**Placebo used**				
yes	2.0352 (1.1085 to 3.7366)	8	2643	0.95
no	1.9818 (1.0173 to 3.861)	9	4328	
**Adjuvant used**				
yes	2.9508 (0.6125 to 14.2157)	3	1042	0.63
no	1.9822 (1.2184 to 3.2248)	14	5929	

Meta-regression	Coefficient (95% confidence intervals)	z-value	P value
**Intrcpt**	2.87 (-2.27 to 8.02)	1.09	
**Mean age**	-0.012 (-0.056 to 0.032)	-0.55	
**Female rate**	-2.74 (-9.84 to 4.35)	-0.76	
**Booster**			
**Placebo**	0.074 (-0.97 to 1.12)	0.14	
**Adjuvant**	0.77 (-1.2 to 2.8)	0.75	

eTable37. Subgroup analysis and meta-regression results of fatigue in seasonal influenza vaccine group

**Fatigue**<br>**Subgroup analysis**	**Risk Ratios (RRs)**	**groups**	**participants**	**P value**
**VRIDV type**				
split vaccine	1.4966 (1.1552 to 1.9389)	12	7563	0.81
recombinant vaccine	1.8072 (0.4634 to 7.0487)	3	329	
inactivated vaccine	1.7034 (1.2346 to 2.3503)	7	1783	
**Concomitant administered vaccine type**				
polysaccharide vaccine	1.1013 (0.9606 to 1.2626)	8	5206	< 0.0001
attenuated live vaccine	na	0	0	
recombinant vaccine	2.2382 (1.4922 to 3.3572)	6	1785	
mRNA Vaccine	2.0885 (1.6526 to 2.6394)	4	438	
inactivated vaccine	1.3165 (1.122 to 1.5446)	4	2246	
**Mean age (year)**				
≥ 65	1.5861 (1.1625 to 2.1642)	11	5280	0.9
< 65	1.6311 (1.1945 to 2.2273)	11	4395	
**Female rate (%)**				
≥ 55	1.6555 (1.234 to 2.2208)	12	5030	0.77
< 55	1.5528 (1.114 to 2.1645)	10	4821	
**Booster vaccine**				
yes				
no				
**Placebo used**				
yes	1.6767 (1.2619 to 2.2279)	13	5342	0.65
no	1.5096 (1.0593 to 2.1513)	9	4333	
**Adjuvant used**				
yes	2.0433 (1.2355 to 3.3792)	4	1134	0.3
no	1.5294 (1.2047 to 1.9417)	18	8541	

**Meta-regression**	**Coefficient (95% confidence intervals)**	**z-value**	**P value**
**Intrcpt**	-0.07 (-2.29 to 2.15)	-0.06	0.95
**Mean age**	-0.004 (-0.026 to 0.018)	-0.36	0.72
**Female rate**	1.2 (-1.68 to 4.08)	0.82	0.41
**Booster**			
**Placebo**	0.13 (-0.36 to 0.62)	0.52	0.6
**Adjuvant**	-0.35 (-0.37 to 0.99)	0.89	0.38

eTable38. Subgroup analysis and meta-regression results of headache in seasonal influenza vaccine group

**Headache**				
**Subgroup analysis**	**Risk Ratios (RRs)**	**groups**	**participants**	**P value**
**VRIDV type**				
split vaccine	1.2919 (0.9788 to 1.7051)	13	8331	0.08
recombinant vaccine	2.3202 (1.459 to 3.6898)	3	327	
inactivated vaccine	1.7241 (1.315 to 2.2604)	7	1778	
**Concomitant administered vaccine type**				
polysaccharide vaccine	1.1018 (0.9764 to 1.2433)	8	5220	< 0.0001
attenuated live vaccine	0.4681 (0.2045 to 1.0715)	1	754	
recombinant vaccine	1.9575 (1.4715 to 2.604)	6	1780	
mRNA Vaccine	2.5164 (1.8935 to 3.3444)	4	436	
inactivated vaccine	1.1283 (0.969 to 1.3139)	4	2246	
**Mean age (year)**				
≥ 65	1.5072 (1.1335 to 2.0041)	11	5257	0.96
< 65	1.4937 (1.1156 to 1.9998)	12	5179	
**Female rate (%)**				
≥ 55	1.5421 (1.1027 to 2.1567)	13	5814	0.81
< 55	1.4638 (1.1403 to 1.8792)	10	4798	
**Booster vaccine**				
yes				
no				
**Placebo used**				
yes	1.4579 (1.1149 to 1.9064)	14	6103	0.68
no	1.5874 (1.1639 to 2.165)	9	4333	
**Adjuvant used**				
yes	1.5588 (1.1843 to 2.0518)	4	1130	0.7
no	1.4539 (1.1553 to 1.8297)	19	9306	

**Meta-regression**	**Coefficient (95% confidence intervals)**	**z-value**	**P value**
**Intrcpt**	0.088 (-2.04 to 2.21)	0.08	0.94
**Mean age**	-0.0083 (-0.029 to 0.012)	-0.79	0.43
**Female rate**	1.52 (-1.27 to 4.31)	1.07	0.29
**Booster**			
**Placebo**	-0.093 (-0.55 to 0.36)	-0.40	0.69
**Adjuvant**	0.25 (-0.4 to 0.91)	0.76	0.44

eTable39. Subgroup analysis and meta-regression results of malaise in seasonal influenza vaccine group

Malaise<br>Subgroup analysis	Risk Ratios (RRs)	groups	participants	P value
**VRIDV type**				
split vaccine	3.0114 (1.9055 to 4.759)	2	304	0.2
recombinant vaccine	4.0321 (0.4636 to 35.065)	2	163	
inactivated vaccine	1.7039 (1.0653 to 2.7254)	6	1021	
**Concomitant administered vaccine type**				
polysaccharide vaccine	2.6168 (0.5937 to 11.5343)	1	163	0.56
attenuated live vaccine	na	0	0	
recombinant vaccine	1.6505 (0.9622 to 2.8313)	4	778	
mRNA Vaccine	2.6173 (1.7743 to 3.8609)	4	432	
inactivated vaccine	2.707 (1.167 to 6.2793)	1	115	
**Mean age (year)**				
≥ 65	2.4413 (1.5632 to 3.8128)	4	563	0.52
< 65	1.9755 (1.2205 to 3.1975)	6	925	
**Female rate (%)**				
≥ 55	2.399 (1.6545 to 3.4784)	5	569	0.37
< 55	1.7611 (0.9975 to 3.1093)	5	919	
**Booster vaccine**				
yes				
no				
**Placebo used**				
yes	1.7694 (1.1526 to 2.7163)	7	1021	0.09
no	2.9748 (1.9211 to 4.6066)	3	467	
**Adjuvant used**				
yes	1.8063 (0.9531 to 3.4235)	3	374	0.66
no	2.1463 (1.392 to 3.3092)	7	1114	

Meta-regression	Coefficient (95% confidence intervals)	z-value	P value
**Intrcpt**	-0.98 (-2.6 to 0.65)	-1.18	0.24
**Mean age**	0.0066 (-0.022 to 0.035)	0.46	0.65
**Female rate**	3.19 (0.38 to 6.01)	2.23	0.026
**Booster**			
**Placebo**	-0.6 (-1.27 to 0.07)	-1.75	0.08
**Adjuvant**	-0.092 (-1.01 to 0.83)	-0.20	0.84

eTable40. Subgroup analysis and meta-regression results of muscle pain in seasonal influenza vaccine group

**Muscle pain**<br>**Subgroup analysis**	**Risk Ratios (RRs)**	**groups**	**participants**	**P value**
**VRIDV type**				
split vaccine	1.7264 (1.4148 to 2.1067)	12	7491	0.25
recombinant vaccine	2.5774 (1.5497 to 4.2866)	2	166	
inactivated vaccine	2.153 (1.4984 to 3.0938)	7	1773	
**Concomitant administered vaccine type**				
polysaccharide vaccine	1.5009 (1.2455 to 1.8087)	7	4974	0.028
attenuated live vaccine				
recombinant vaccine	2.3577 (1.7477 to 3.1806)	6	1779	
mRNA Vaccine	2.4466 (1.6477 to 3.6329)	4	431	
inactivated vaccine	1.6925 (1.0634 to 2.6938)	4	2246	
**Mean age (year)**				
≥ 65	1.6817 (1.4126 to 2.0022)	10	5079	0.204
< 65	2.0993 (1.5641 to 2.8176)	11	4351	
**Female rate (%)**				
≥ 55	1.9433 (1.5057 to 2.5081)	13	4986	0.66
< 55	1.8481 (1.4696 to 2.3242)	8	4620	
**Booster vaccine**				
yes				
no				
**Placebo used**				
yes	1.9433 (1.5206 to 2.4835)	13	5257	0.74
no	1.8357 (1.4429 to 2.3355)	8	4173	
**Adjuvant used**				
yes	1.6012 (1.2922 to 1.9842)	5	1128	0.17
no	1.9745 (1.5955 to 2.4436)	16	8302	

**Meta-regression**	**Coefficient (95% confidence intervals)**	**z-value**	**P value**
**Intrcpt**	0.66 (-1.63 to 2.94)	0.56	0.57
**Mean age**	-0.007 (-0.024 to 0.01)	-0.79	0.43
**Female rate**	0.8 (-2.19 to 3.79)	0.52	0.59
**Booster**			
**Placebo**	-0.0034 (-0.39 to 0.38)	-0.01	0.98
**Adjuvant**	-0.021(-0.69 to 0.28)	-0.83	0.41

eTable41. Subgroup analysis and meta-regression results of gastrointestinal symptoms in seasonal influenza vaccine group

**gastrointestinal symptoms**<br>**Subgroup analysis**	**Risk Ratios (RRs)**	**groups**	**participants**	**P value**
**VRIDV type**				
split vaccine	1.4095 (1.0508 to 1.8905)	11	6178	0.67
recombinant vaccine	1.4536 (0.7818 to 2.7028)	2	162	
inactivated vaccine	1.9531 (1.0038 to 3.8)	5	845	
**Concomitant administered vaccine type**				
polysaccharide vaccine	1.1608 (0.9546 to 1.4114)	4	2743	< 0.0001
attenuated live vaccine	na	0	0	
recombinant vaccine	1.8051 (1.1731 to 2.7777)	6	1771	
mRNA Vaccine	2.8529 (1.7111 to 4.7567)	4	425	
inactivated vaccine	1.1863 (0.9457 to 1.4881)	4	2246	
**Mean age (year)**				
≥ 65	1.5326 (0.883 to 2.6601)	8	4136	0.95
< 65	1.5589 (1.2409 to 1.9584)	10	3049	
**Female rate (%)**				
≥ 55	1.6888 (1.2242 to 2.3296)	9	2766	0.43
< 55	1.3854 (0.9512 to 2.0178)	9	4595	
**Booster vaccine**				
yes				
no				
**Placebo used**				
yes	1.4531 (1.0568 to 1.998)	12	3930	0.65
no	1.6505 (1.0497 to 2.5952)	6	3255	
**Adjuvant used**				
yes	2.0154 (0.5344 to 7.6008)	2	207	0.66
no	1.501 (1.1611 to 1.9405)	16	6978	

**Meta-regression**	**Coefficient (95% confidence intervals)**	**z-value**	**P value**
**Intrcpt**	-2.64 (-5.3 to 0.02)	-1.94	0.052
**Mean age**	0.0058 (-0.015 to 0.027)	0.54	0.59
**Female rate**	5.07 (1.62 to 8.51)	2.89	0.0039
**Booster**			
**Placebo**	-0.14 (-0.59 to 0.31)	-0.61	0.54
**Adjuvant**	0.57 (-0.73 to 1.87)	0.86	0.39

eTable42. Subgroup analysis and meta-regression results of chills in seasonal influenza vaccine group

Chills<br>Subgroup analysis	Risk Ratios (RRs)	groups	participants	P value
**VRIDV type**				
split vaccine	2.2945 (1.4868 to 3.5408)	8	4669	0.39
recombinant vaccine	7.4261 (1.3885 to 39.7164)	2	164	
inactivated vaccine	2.2328 (1.491 to 3.3436)	6	1363	
**Concomitant administered vaccine type**				
polysaccharide vaccine	1.5717 (1.2939 to 1.9091)	5	2747	< 0.0001
attenuated live vaccine	na	0	0	
recombinant vaccine	3.5865 (2.6001 to 4.9471)	5	1370	
mRNA Vaccine	4.0239 (1.9151 to 8.4548)	4	428	
inactivated vaccine	1.523 (1.0743 to 2.1591)	2	1651	
**Mean age (year)**				
≥ 65	2.6349 (1.6321 to 4.2538)	9	4161	0.64
< 65	2.2744 (1.5095 to 3.427)	7	2035	
**Female rate (%)**				
≥ 55	2.5081 (1.5695 to 4.0079)	9	2313	0.86
< 55	2.376 (1.5466 to 3.6502)	7	4059	
**Booster vaccine**				
yes				
no				
**Placebo used**				
yes	2.3729 (1.5692 to 3.5885)	10	2618	0.9
no	2.4682 (1.5164 to 4.0174)	6	3578	
**Adjuvant used**				
yes	2.77 (1.2588 to 6.0952)	4	1127	0.72
no	2.3685 (1.6781 to 3.3429)	12	5069	

Meta-regression	Coefficient (95% confidence intervals)	z-value	P value
**Intrcpt**	-0.83 (-7.89 to 6.24)	-0.23	0.82
**Mean age**	0.014 (-0.046 to 0.073)	0.45	0.65
**Female rate**	1.46 (-5.37 to 8.29)	0.42	0.68
**Booster**			
**Placebo**	0.1 (-0.7 to 0.9)	0.25	0.81
**Adjuvant**	0.0016 (-1.14 to 1.15)	0	1

eTable43. meta-regression results of erythra in seasonal influenza vaccine group

**Erythra (k=5)**			
**Meta-regression**	**Coefficient (95% confidence intervals)**	**z-value**	**P value**
**Intrcpt**	4.54 (-29.9 to 38.97)	0.26	0.8
**Mean age**	-0.048 (-0.31 to 0.21)	-0.37	0.71
**Female rate**	-4.12 (-43.08 to 34.84)	-0.21	0.84
**Booster**			
**Placebo**	1.43 (-1.9 to 4.75)	0.84	0.4
**Adjuvant**			

eTable44. Subgroup analysis and meta-regression results of arthralgia in seasonal influenza vaccine group

**Arthralgia**<br>**Subgroup analysis**	**Risk Ratios (RRs)**	**groups**	**participants**	**P value**
**VRIDV type**				
split vaccine	1.6046 (1.1681 to 2.2043)	10	6008	0.46
recombinant vaccine	1.7069 (0.6707 to 4.3438)	3	325	
inactivated vaccine	2.3491 (1.4105 to 3.9121)	7	1768	
**Concomitant administered vaccine type**				
polysaccharide vaccine	1.1412 (0.9331 to 1.3957)	7	4131	< 0.0001
attenuated live vaccine				
recombinant vaccine	3.4277 (2.2594 to 5.2)	6	1775	
mRNA Vaccine	2.6898 (1.6188 to 4.4693)	4	429	
inactivated vaccine	1.4811 (1.1778 to 1.8627)	3	1766	
**Mean age (year)**				
≥ 65	1.686 (1.2212 to 2.3277)	10	4341	0.66
< 65	1.8957 (1.2559 to 2.8615)	10	3760	
**Female rate (%)**				
≥ 55	1.9648 (1.2454 to 3.0997)	11	3519	0.58
< 55	1.6923 (1.3117 to 2.1834)	9	4758	
**Booster vaccine**				
yes				
no				
**Placebo used**				
yes	2.1432 (1.3997 to 3.2818)	12	4248	0.23
no	1.5715 (1.1859 to 2.0824)	8	3853	
**Adjuvant used**				
yes	1.9225 (1.0460 to 3.5335)	5	1127	0.78
no	1.7494 (1.3092 to 2.3378)	15	6974	

**Meta-regression**	**Coefficient (95% confidence intervals)**	**z-value**	**P value**
**Intrcpt**	-0.047 (-3.42 to 3.33)	-0.027	0.98
**Mean age**	-0.006 (-0.035 to 0.02)	-0.42	0.68
**Female rate**	1.56 (-2.58 to 5.71)	0.74	0.46
**Booster**			
**Placebo**	0.25 (-0.34 to 0.83)	0.83	0.96
**Adjuvant**	0.019 (-0.79 to 0.83)	0.047	0.96

eTable45. Subgroup analysis and meta-regression results of local pain in seasonal influenza vaccine group

Local Pain				
**Subgroup analysis**	**Risk Ratios (RRs)**	**groups**	**participants**	**P value**
**VRIDV type**				
split vaccine	1.43 (1.1908 to 1.7172)	14	9426	0.048
recombinant vaccine	1.0499 (0.8357 to 1.3189)	3	329	
inactivated vaccine	1.5262 (1.2058 to 1.9317)	7	1792	
**Concomitant administered vaccine type**				
polysaccharide vaccine	1.7587 (1.3108 to 2.3598)	8	5441	0.0002
attenuated live vaccine	1.0068 (0.8915 to 1.137)	2	1627	
recombinant vaccine	1.4731 (1.1719 to 1.8517)	6	1791	
mRNA Vaccine	1.1298 (0.9396 to 1.3585)	4	443	
inactivated vaccine	1.491 (1.2018 to 1.8497)	4	2245	
**Mean age (year)**				
≥ 65	1.5891 (1.4128 to 1.7874)	11	5472	0.07
< 65	1.2875 (1.0604 to 1.5633)	13	6075	
**Female rate (%)**				
≥ 55	1.3134 (1.0921 to 1.5795)	14	6708	0.16
< 55	1.5612 (1.3318 to 1.8302)	10	5454	
**Booster vaccine**				
yes				
no				
**Placebo used**				
yes	1.3608 (1.1175 to 1.6571)	15	7213	0.42
no	1.5 (1.3153 to 1.7106)	9	4334	
**Adjuvant used**				
yes	1.6811 (1.3963 to 2.0241)	4	1134	0.1
no	1.3781 (1.1849 to 1.6029)	20	10413	

**Meta-regression**	**Coefficient (95% confidence intervals)**	**z-value**	**P value**
**Intrcpt**	1.32 (-0.02 to 2.65)	1.94	0.053
**Mean age**	-0.0041 (-0.018 to 0.0094)	-0.60	0.55
**Female rate**	-1.26 (-2.99 to 0.47)	-1.43	0.15
**Booster**			
**Placebo**	-0.099 (-0.38 to 0.18)	-0.70	0.49
**Adjuvant**	0.23 (-0.17 to 0.63)	1.12	0.26

eTable46. Subgroup analysis and meta-regression results of swelling in seasonal influenza vaccine group

**Swelling**<br>**Subgroup analysis**	**Risk Ratios (RRs)**	**groups**	**participants**	**P value**
**VRIDV type**				
split vaccine	1.5385 (1.072 to 2.2081)	14	9187	0.48
recombinant vaccine	0.9432 (0.4448 to 2.0002)	3	325	
inactivated vaccine	1.2387 (0.6819 to 2.25)	7	1763	
**Concomitant administered vaccine type**				
polysaccharide vaccine	1.9304 (1.0182 to 3.6601)	8	5202	0.32
attenuated live vaccine	1.0534 (0.7737 to 1.4342)	2	1627	
recombinant vaccine	1.3816 (0.7572 to 2.5211)	6	1775	
mRNA Vaccine	0.5886 (0.141 to 2.456)	4	426	
inactivated vaccine	1.4888 (0.9147 to 2.4234)	4	2245	
**Mean age (year)**				
≥ 65	1.7113 (1.3062 to 2.242)	11	5466	0.09
< 65	1.1133 (0.7304 to 1.6969)	13	5809	
**Female rate (%)**				
≥ 55	1.2492 (0.8296 to 1.881)	14	6442	0.3
< 55	1.6302 (1.2086 to 2.1987)	10	5448	
**Booster vaccine**				
yes				
no				
**Placebo used**				
yes	1.3365 (0.8273 to 2.1592)	15	6941	0.77
no	1.4456 (1.1135 to 1.8769)	9	4334	
**Adjuvant used**				
yes	1.5756 (0.6696 to 3.7072)	4	1128	0.74
no	1.3537 (0.9958 to 1.8404)	20	10147	

**Meta-regression**	**Coefficient (95% confidence intervals)**	**z-value**	**P value**
**Intrcpt**	0.098 (-3.53 to 3.72)	0.05	0.96
**Mean age**	0.016 (-0.019 to 0.05)	0.88	0.38
**Female rate**	-1.3 (-5.86 to 3.27)	-0.56	0.58
**Booster**			
**Placebo**	0.0057 (-0.65 to 0.67)	0.02	0.99
**Adjuvant**	0.11 (-0.93 to 1.15)	0.2	0.84

eTable47. Subgroup analysis and meta-regression results of itch in seasonal influenza vaccine group

**Itch**<br>**Subgroup analysis**	**Risk Ratios (RRs)**	**groups**	**participants**	**P value**
**VRIDV type**				
split vaccine	0.9927 (0.67 to 1.4708)	6	3553	0.75
recombinant vaccine	1.221 (0.7046 to 2.1159)	3	322	
inactivated vaccine	0.8589 (0.3565 to 2.0697)	4	442	
**Concomitant administered vaccine type**				
polysaccharide vaccine	1.1657 (0.6338 to 2.1441)	2	1311	0.32
attenuated live vaccine	1.0057 (0.6439 to 1.5708)	2	1627	
recombinant vaccine	1.8595 (0.7513 to 4.6023)	4	549	
mRNA Vaccine	0.3599 (0.1062 to 1.2191)	3	235	
inactivated vaccine	0.9402 (0.3746 to 2.3597)	2	595	
**Mean age (year)**				
≥ 65	0.8909 (0.5194 to 1.5279)	5	1699	0.53
< 65	1.0959 (0.7632 to 1.5737)	8	2618	
**Female rate (%)**				
≥ 55	1.0707 (0.7399 to 1.5492)	5	2683	0.72
< 55	0.9548 (0.5701 to 1.5993)	8	2072	
**Booster vaccine**				
yes				
no				
**Placebo used**				
yes	0.989 (0.6841 to 1.4299)	10	3562	0.67
no	1.1308 (0.6733 to 1.8991)	3	755	
**Adjuvant used**				
yes	0.1935 (0.0348 to 1.0764)	2	208	0.04
no	1.1255 (0.8257 to 1.5343)	11	4109	

**Meta-regression**	**Coefficient (95% confidence intervals)**	**z-value**	**P value**
**Intrcpt**	2.47 (-2.83 to 7.76)	0.91	0.36
**Mean age**	-0.011 (-0.057 to 0.034)	-0.49	0.62
**Female rate**	-3.61 (-10.34 to 3.12)	-1.05	0.29
**Booster**			
**Placebo**	0.4 (-0.72 to 1.53)	0.71	0.48
**Adjuvant**	-1.81 (-3.7 to 0.07)	-1.89	0.059

eTable48. Subgroup analysis and meta-regression results of erythema in seasonal influenza vaccine group

Erythema<br>Subgroup analysis	Risk Ratios (RRs)	groups	participants	P value
**VRIDV type**				
split vaccine	1.4829 (1.0655 to 2.0638)	14	9181	0.3
recombinant vaccine	0.9636 (0.6222 to 1.4923)	3	325	
inactivated vaccine	1.3232 (0.7842 to 2.2327)	7	1764	
**Concomitant administered vaccine type**				
polysaccharide vaccine	1.8631 (0.9854 to 3.5226)	8	5196	0.34
attenuated live vaccine	1.2614 (0.9511 to 1.673)	2	1627	
recombinant vaccine	0.9184 (0.6569 to 1.284)	6	1776	
mRNA Vaccine	1.3059 (0.578 to 2.9504)	4	426	
inactivated vaccine	1.2257 (1.026 to 1.4641)	4	2245	
**Mean age (year)**				
≥ 65	1.5783 (1.1497 to 2.1667)	11	5466	0.22
< 65	1.1843 (0.8497 to 1.6507)	13	5804	
**Female rate (%)**				
≥ 55	1.3757 (0.9598 to 1.9717)	14	6437	0.78
< 55	1.2889 (0.9506 to 1.7478)	10	5448	
**Booster vaccine**				
yes				
no				
**Placebo used**				
yes	1.2748 (0.8905 to 1.825)	15	6936	0.66
no	1.4159 (1.0486 to 1.912)	9	4334	
**Adjuvant used**				
yes	1.7442 (1.2185 to 2.4966)	4	1128	0.15
no	1.2691 (0.9815 to 1.6409)	20	10142	

Meta-regression	Coefficient (95% confidence intervals)	z-value	P value
**Intrcpt**	-0.017 (-3.05 to 3.01)	-0.01	0.99
**Mean age**	0.0047 (-0.024 to 0.033)	0.32	0.75
**Female rate**	0.05 (-3.78 to 3.88)	0.03	0.98
**Booster**			
**Placebo**	-0.086 (-0.63 to 0.46)	-0.31	0.76
**Adjuvant**	0.38 (-0.46 to 1.21)	0.88	0.38

eFigure 1. Immunogenicity of concomitant vaccination vs sequential vaccination for SARS-COV-2 SCR group in the meta-analysis.

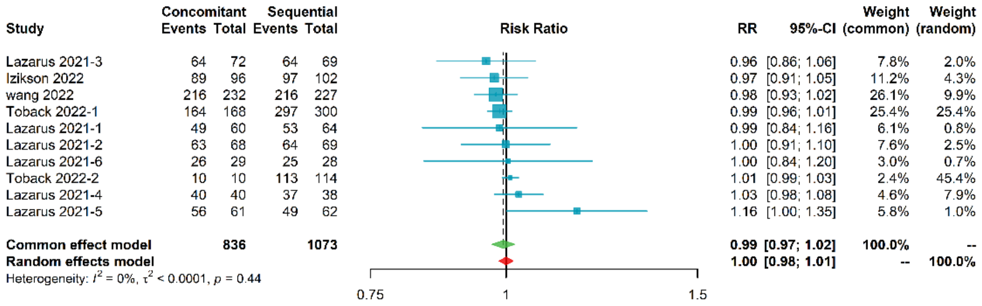

eFigure 2. Immunogenicity of concomitant vaccination vs sequential vaccination for SARS-COV-2 GMT group in the meta-analysis.

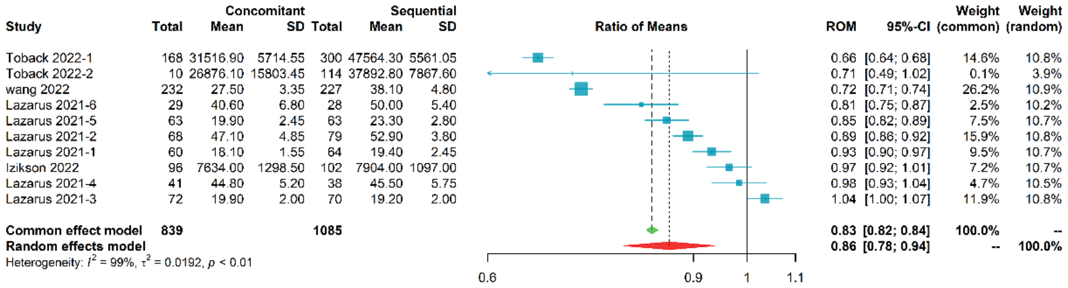

eFigure 3. Immunogenicity of concomitant vaccination vs sequential vaccination for SARS-COV-2 GMFR group in the meta-analysis.

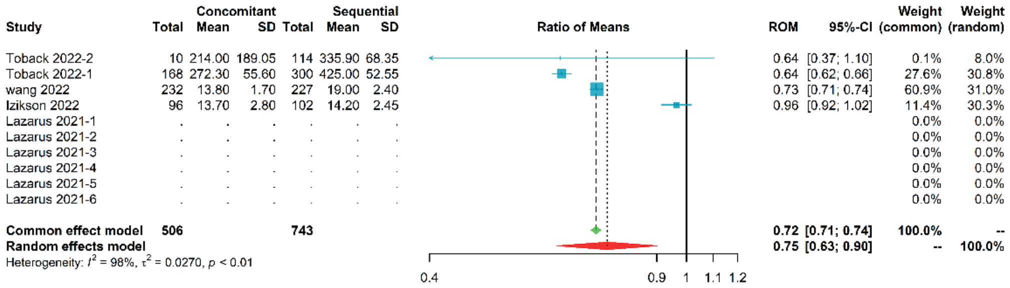

eFigure 4. Immunogenicity of concomitant vaccination vs sequential vaccination for seasonal influenza A/H1N1 strain SCR group in the meta-analysis

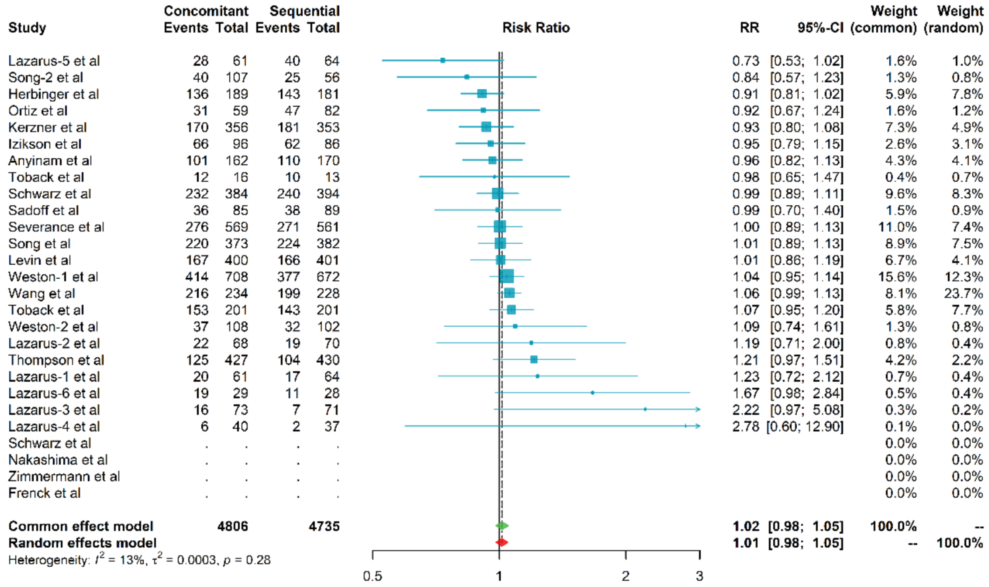

eFigure 5. Immunogenicity of concomitant vaccination vs sequential vaccination for seasonal influenza A/H1N1 strain SPR group in the meta-analysis

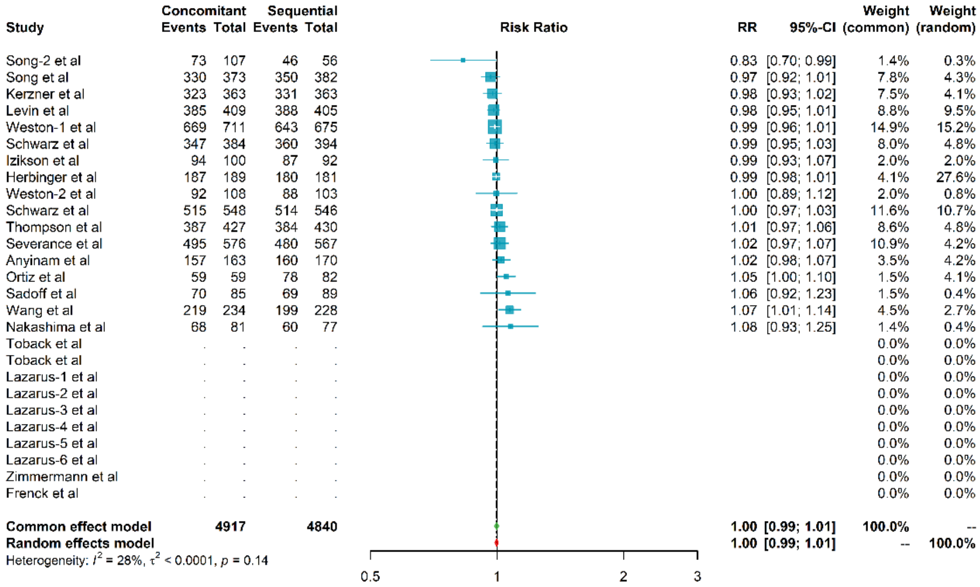

eFigure 6. Immunogenicity of concomitant vaccination vs sequential vaccination for seasonal influenza A/H1N1 strain GMT group in the meta-analysis

Study	Concomitant group Total	Concomitant group Mean	Concomitant group SD	Sequential group Total	Sequential group Mean	Sequential group SD	ROM	95%-CI	Weight (common)	Weight (random)
Song-2 et al	55	55.50	18.30	56	70.60	19.55	0.79	[0.70; 0.88]	0.2%	3.6%
Herbinger et al	189	565.00	150.50	181	702.00	192.00	0.80	[0.76; 0.85]	0.7%	4.1%
Lazarus-5 et al	63	7.90	0.95	64	9.30	1.10	0.85	[0.81; 0.89]	1.2%	4.2%
Weston-2 et al	108	79.90	16.65	103	89.30	18.35	0.89	[0.85; 0.95]	0.7%	4.1%
Kerzner et al	363	122.90	16.10	363	134.20	21.95	0.92	[0.90; 0.94]	4.4%	4.3%
Zimmermann et al	463	177.00	26.00	448	189.00	27.50	0.94	[0.92; 0.95]	5.7%	4.3%
Schwarz et al	384	187.50	22.05	394	194.30	22.55	0.97	[0.95; 0.98]	7.6%	4.3%
Izikson et al	96	363.00	50.00	86	366.00	109.50	0.99	[0.93; 1.06]	0.4%	4.0%
Lazarus-1 et al	61	6.10	0.80	64	6.10	0.80	1.00	[0.96; 1.05]	1.0%	4.2%
Frenck et al	530	331.80	32.80	531	330.60	134.05	1.00	[0.97; 1.04]	1.6%	4.2%
Thompson et al	427	115.00	11.40	430	113.00	11.50	1.02	[1.00; 1.03]	11.3%	4.3%
Schwarz et al	548	195.50	19.90	546	191.90	19.55	1.02	[1.01; 1.03]	14.0%	4.3%
Levin et al	409	207.30	23.05	405	200.50	21.30	1.03	[1.02; 1.05]	9.2%	4.3%
Weston-1 et al	711	189.10	18.30	675	181.60	16.90	1.04	[1.03; 1.05]	20.4%	4.3%
Lazarus-2 et al	68	6.70	0.70	70	6.30	0.80	1.06	[1.02; 1.11]	1.4%	4.2%
Ortiz et al	59	547.70	132.55	53	503.60	171.25	1.09	[0.97; 1.21]	0.2%	3.7%
Toback et al	201	186.50	38.80	201	170.60	32.85	1.09	[1.05; 1.14]	1.3%	4.2%
Lazarus-3 et al	73	5.20	0.55	71	4.60	0.55	1.13	[1.09; 1.17]	1.5%	4.2%
Severance et al	576	127.35	18.50	567	112.60	12.25	1.13	[1.11; 1.15]	9.2%	4.3%
Lazarus-4 et al	41	5.90	0.85	37	5.20	0.90	1.13	[1.06; 1.22]	0.4%	4.0%
Lazarus-6 et al	29	10.90	1.70	28	9.00	1.85	1.21	[1.10; 1.33]	0.2%	3.8%
Song et al	373	169.40	23.10	382	136.50	16.35	1.24	[1.22; 1.26]	6.1%	4.3%
Sadoff et al	85	215.00	64.50	89	168.00	50.50	1.28	[1.17; 1.40]	0.3%	3.9%
Wang et al	234	462.00	99.87	228	342.00	80.65	1.35	[1.30; 1.41]	1.2%	4.2%
Toback et al	16	159.00	92.15	13	112.80	142.90	1.41	[0.67; 2.97]	0.0%	0.5%
Anyinam et al	.	.	.	.	.	.			0.0%	0.0%
Nakashima et al	.	.	.	.	.	.			0.0%	0.0%
**Common effect model**	**6162**			**6085**			**1.04**	**[1.03; 1.04]**	**100.0%**	--
**Random effects model**							**1.03**	**[0.98; 1.09]**	--	**100.0%**

Heterogeneity: $I^2 = 98\%$, $\tau^2 = 0.0174$, $p < 0.01$

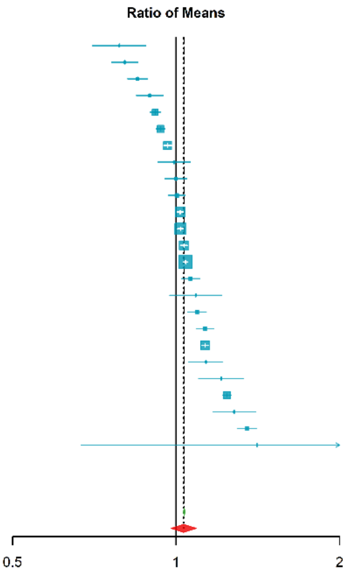

eFigure 7. Immunogenicity of concomitant vaccination vs sequential vaccination for seasonal influenza A/H3N2 strain SCR group in the meta-analysis

Study	Concomitant Events	Concomitant Total	Sequential Events	Sequential Total	RR	95%-CI	Weight (common)	Weight (random)
Ortiz et al	29	59	51	82	0.79	[0.58; 1.08]	1.7%	1.8%
Thompson et al	119	427	136	430	0.88	[0.72; 1.08]	5.3%	3.5%
Herbinger et al	134	189	145	181	0.89	[0.79; 0.99]	5.8%	7.8%
Lazarus-2 et al	20	68	23	70	0.90	[0.54; 1.47]	0.9%	0.7%
Izikson et al	42	96	41	86	0.92	[0.67; 1.26]	1.7%	1.7%
Toback et al	8	16	7	13	0.93	[0.46; 1.87]	0.3%	0.4%
Wang et al	216	234	214	228	0.98	[0.94; 1.03]	8.4%	14.3%
Sadoff et al	47	85	50	89	0.98	[0.76; 1.28]	1.9%	2.3%
Kerzner et al	276	356	274	353	1.00	[0.92; 1.08]	10.7%	11.1%
Schwarz et al	136	384	139	394	1.00	[0.83; 1.21]	5.3%	4.0%
Song-2 et al	37	107	19	56	1.02	[0.65; 1.60]	1.0%	0.9%
Levin et al	305	400	299	401	1.02	[0.94; 1.11]	11.6%	11.1%
Anyinam et al	61	162	62	170	1.03	[0.78; 1.37]	2.4%	2.1%
Weston-2 et al	72	108	64	102	1.06	[0.87; 1.30]	2.6%	3.7%
Weston-1 et al	560	708	494	672	1.08	[1.01; 1.14]	19.7%	13.3%
Toback et al	136	201	125	201	1.09	[0.94; 1.26]	4.9%	6.0%
Severance et al	158	569	142	561	1.10	[0.90; 1.33]	5.6%	3.9%
Lazarus-5 et al	41	61	37	64	1.16	[0.88; 1.53]	1.4%	2.2%
Lazarus-6 et al	21	29	17	28	1.19	[0.82; 1.73]	0.7%	1.3%
Song et al	226	373	190	382	1.22	[1.07; 1.39]	7.3%	6.9%
Lazarus-3 et al	8	73	6	70	1.28	[0.47; 3.50]	0.2%	0.2%
Lazarus-4 et al	5	40	3	37	1.54	[0.40; 6.01]	0.1%	0.1%
Lazarus-1 et al	21	60	14	64	1.60	[0.90; 2.85]	0.5%	0.6%
Schwarz et al	.	.	.	.			0.0%	0.0%
Nakashima et al	.	.	.	.			0.0%	0.0%
Zimmermann et al	.	.	.	.			0.0%	0.0%
Frenck et al	.	.	.	.			0.0%	0.0%
**Common effect model**		**4805**		**4734**	**1.03**	**[1.00; 1.07]**	**100.0%**	--
**Random effects model**					**1.02**	**[0.98; 1.07]**	--	**100.0%**

Heterogeneity: $I^2 = 26\%$, $\tau^2 = 0.0028$, $p = 0.13$

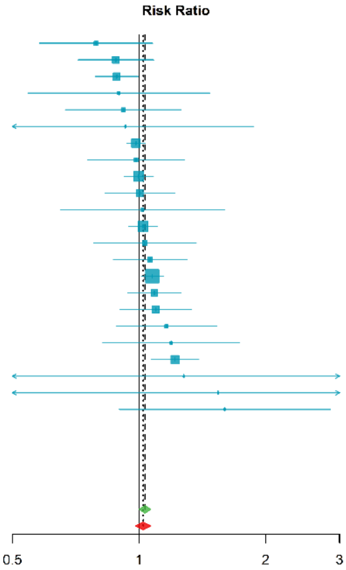

eFigure 8. Immunogenicity of concomitant vaccination vs sequential vaccination for seasonal influenza A/H3N2 strain SPR group in the meta-analysis

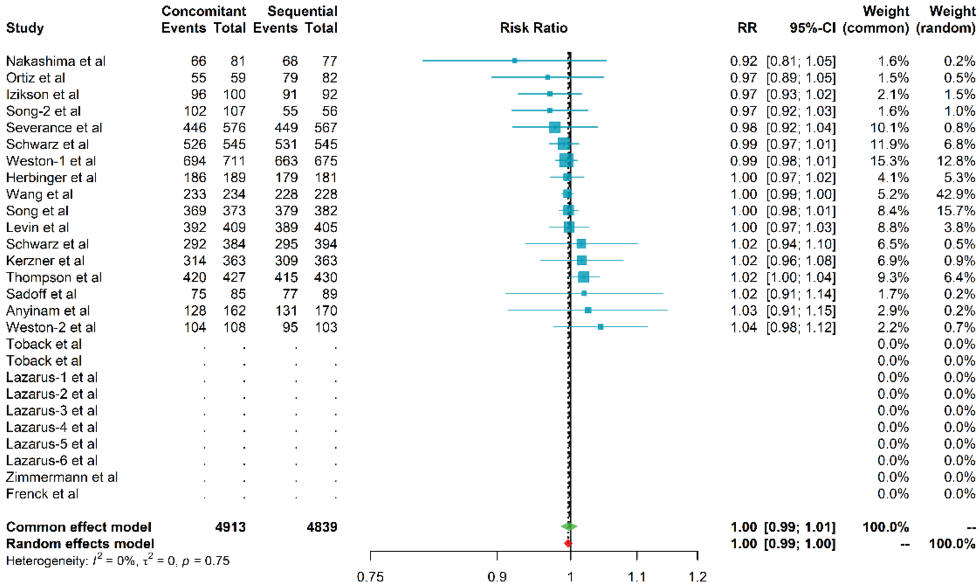

eFigure 9. Immunogenicity of concomitant vaccination vs sequential vaccination for seasonal influenza A/H3N2 strain GMT group in the meta-analysis

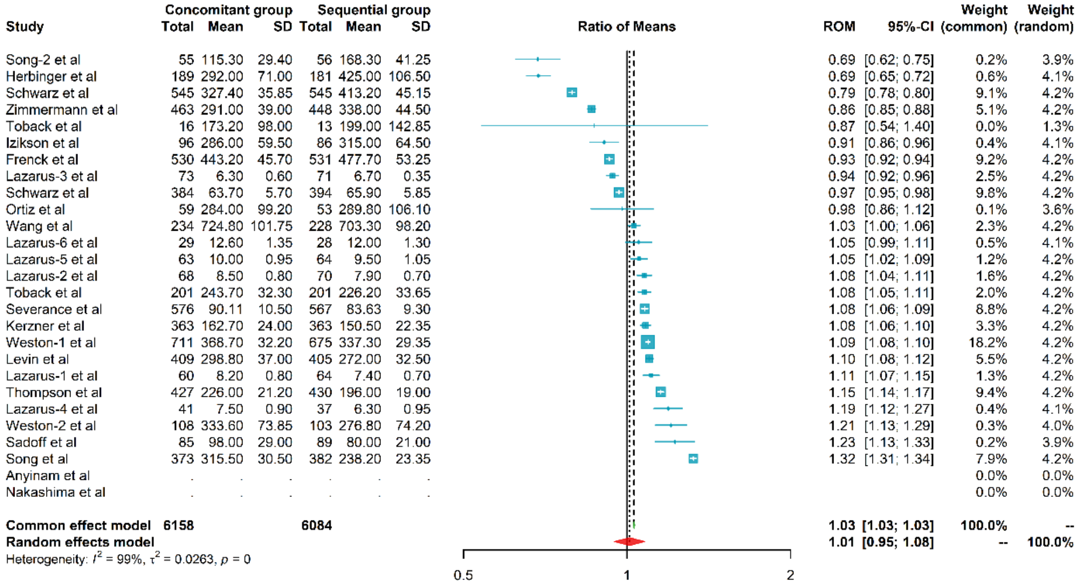

eFigure 10. Immunogenicity of concomitant vaccination vs sequential vaccination for seasonal influenza B strain SCR group in the meta-analysis

Study	Concomitant Events	Concomitant Total	Sequential Events	Sequential Total	RR	95%-CI	Weight (common)	Weight (random)
Toback 2022-2-Victoria	2	16	4	13	0.41	[0.09; 1.88]	0.2%	0.1%
Lazarus 2021-3-Victoria	1	73	2	71	0.49	[0.05; 5.24]	0.1%	0.0%
Ortiz 2022-Victoria	17	59	39	82	0.61	[0.38; 0.96]	1.1%	0.6%
Lazarus 2021-1-Yamagata	9	61	13	64	0.73	[0.33; 1.58]	0.4%	0.2%
Anyinam 2017-Victoria	55	162	73	170	0.79	[0.60; 1.04]	2.4%	1.6%
Ortiz 2022-Yamagata	11	59	19	82	0.80	[0.41; 1.56]	0.5%	0.3%
Izikson 2022-Yamagata	17	96	18	86	0.85	[0.47; 1.53]	0.6%	0.3%
Lazarus 2021-5-Victoria	13	61	16	64	0.85	[0.45; 1.62]	0.5%	0.3%
Schwarz 2017-Texas	143	384	169	394	0.87	[0.73; 1.03]	5.7%	3.9%
Kerzner 2007-Shanghai	211	356	233	353	0.90	[0.80; 1.01]	8.0%	8.3%
Weston 2012-2	41	108	43	102	0.90	[0.65; 1.25]	1.5%	1.1%
Lazarus 2021-4-Victoria	1	40	1	37	0.92	[0.06; 14.26]	0.0%	0.0%
Thompson 2019-Massachusetts	99	427	106	430	0.94	[0.74; 1.19]	3.6%	2.1%
Anyinam 2017-Yamagata	54	162	60	170	0.94	[0.70; 1.27]	2.0%	1.3%
Wang 2022-Yamagata	193	234	199	228	0.94	[0.87; 1.02]	6.9%	15.9%
Thompson 2019-Brisbane	91	427	96	430	0.95	[0.74; 1.23]	3.3%	1.9%
Herbinger 2014-Malaysia	147	189	147	181	0.96	[0.86; 1.06]	5.1%	9.8%
Wang 2022-Victoria	183	234	184	228	0.97	[0.88; 1.06]	6.4%	11.8%
Toback 2022-1-Yamagata	65	201	65	201	1.00	[0.75; 1.33]	2.2%	1.5%
Lazarus 2021-2-Yamagata	9	68	9	68	1.00	[0.42; 2.36]	0.3%	0.2%
Levin 2018-Yamagata	151	400	150	401	1.01	[0.84; 1.21]	5.1%	3.6%
Levin 2018-Victoria	124	400	123	401	1.01	[0.82; 1.24]	4.2%	2.7%
Severance 2022-Victoria	166	569	161	561	1.02	[0.85; 1.22]	5.5%	3.5%
Severance 2022-Yamagata	177	569	171	561	1.02	[0.86; 1.22]	5.9%	3.8%
Weston 2012-1	463	708	425	672	1.03	[0.96; 1.12]	14.9%	15.4%
Sadoff 2021-Phuket?	39	85	39	89	1.05	[0.75; 1.46]	1.3%	1.1%
Schwarz 2017-Brisbane	154	384	148	394	1.07	[0.89; 1.27]	5.0%	3.7%
Lazarus 2021-5-Yamagata	23	61	22	64	1.10	[0.69; 1.75]	0.7%	0.6%
Sadoff 2021-Brisbane	36	85	33	89	1.14	[0.79; 1.65]	1.1%	0.9%
Izikson 2022-Victoria	29	96	21	86	1.24	[0.77; 2.00]	0.8%	0.5%
Song 2017-Massachusetts	117	373	86	382	1.39	[1.10; 1.77]	2.9%	2.1%
Toback 2022-1-Victoria	29	201	20	201	1.45	[0.85; 2.48]	0.7%	0.4%
Song-2 2015-Massachusetts	22	107	7	56	1.64	[0.75; 3.61]	0.3%	0.2%
Lazarus 2021-6-Victoria	11	29	6	28	1.77	[0.76; 4.13]	0.2%	0.2%
Lazarus 2021-1-Victoria	7	61	4	64	1.84	[0.57; 5.96]	0.1%	0.1%
Lazarus 2021-6-Yamagata	17	29	7	28	2.34	[1.15; 4.77]	0.2%	0.2%
Lazarus 2021-2-Victoria	6	68	2	70	3.09	[0.65; 14.77]	0.1%	0.0%
Schwarz 2011	.	.	.	.			0.0%	0.0%
Nakashima 2018	.	.	.	.			0.0%	0.0%
Zimmermann 2013-Brisbane	.	.	.	.			0.0%	0.0%
Frenck 2012-Malaysia	.	.	.	.			0.0%	0.0%
**Common effect model**		**7642**		**7601**	**1.00**	**[0.96; 1.03]**	**100.0%**	--
**Random effects model**					**0.98**	**[0.95; 1.02]**	--	**100.0%**

Heterogeneity: $I^2 = 17\%$, $\tau^2 = 0.0005$, $p = 0.19$

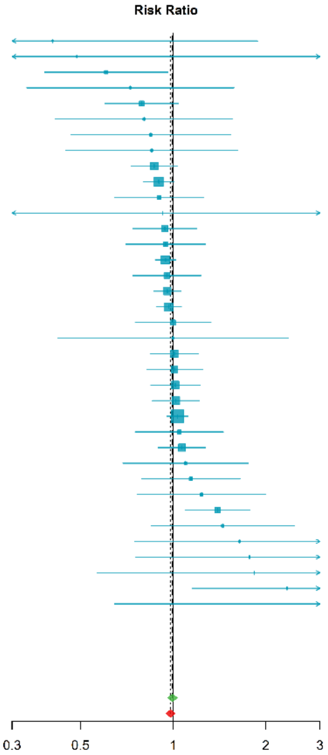

eFigure 11. Immunogenicity of concomitant vaccination vs sequential vaccination for seasonal influenza B strain SPR group in the meta-analysis

Study	Concomitant Events	Concomitant Total	Sequential Events	Sequential Total	RR	95%-CI	Weight (common)	Weight (random)
Ortiz 2022-Yamagata	15	59	34	82	0.61	[0.37; 1.02]	0.5%	0.0%
Nakashima 2018-Texas	33	81	45	77	0.70	[0.50; 0.96]	0.8%	0.0%
Ortiz 2022-Victoria	32	59	60	82	0.74	[0.57; 0.97]	0.9%	0.0%
Kerzner 2007-Shanghai	312	363	326	363	0.96	[0.91; 1.01]	5.9%	1.1%
Herbinger 2014-Malaysia	147	189	147	181	0.96	[0.86; 1.06]	2.7%	0.3%
Anyinam 2017-Victoria	154	162	167	170	0.97	[0.93; 1.01]	3.0%	1.9%
Wang 2022-Yamagata	220	234	221	228	0.97	[0.93; 1.01]	4.1%	2.0%
Nakashima 2018-Phuket?	49	81	48	77	0.97	[0.76; 1.24]	0.9%	0.1%
Wang 2022-Victoria	197	234	195	228	0.98	[0.91; 1.06]	3.6%	0.5%
Sadoff 2021-Brisbane	48	85	51	89	0.99	[0.76; 1.28]	0.9%	0.0%
Weston 2012-1	685	711	654	675	0.99	[0.97; 1.01]	12.2%	7.8%
Schwarz 2017-Texas	372	384	382	394	1.00	[0.97; 1.02]	6.8%	4.9%
Schwarz 2017-Brisbane	383	384	393	394	1.00	[0.99; 1.01]	7.0%	48.2%
Izikson 2022-Victoria	99	100	91	92	1.00	[0.97; 1.03]	1.7%	3.6%
Severance 2022-Victoria	317	576	311	567	1.00	[0.90; 1.11]	5.7%	0.3%
Song 2017-Massachusetts	271	373	276	382	1.01	[0.92; 1.10]	4.9%	0.4%
Anyinam 2017-Yamagata	162	162	169	170	1.01	[0.99; 1.02]	3.0%	21.3%
Schwarz 2011	449	548	444	546	1.01	[0.95; 1.07]	8.1%	1.0%
Weston 2012-2	107	108	101	103	1.01	[0.98; 1.04]	1.9%	2.9%
Izikson 2022-Yamagata	99	100	90	92	1.01	[0.98; 1.05]	1.7%	2.4%
Song-2 2015-Massachusetts	84	107	43	56	1.02	[0.86; 1.22]	1.0%	0.1%
Severance 2022-Yamagata	302	576	288	567	1.03	[0.92; 1.15]	5.3%	0.2%
Thompson 2019-Massachusetts	281	427	272	430	1.04	[0.94; 1.15]	4.9%	0.3%
Thompson 2019-Brisbane	210	427	200	430	1.06	[0.92; 1.22]	3.6%	0.2%
Levin 2018-Yamagata	272	409	249	405	1.08	[0.98; 1.20]	4.5%	0.3%
Levin 2018-Victoria	220	409	201	405	1.08	[0.95; 1.24]	3.7%	0.2%
Sadoff 2021-Phuket?	47	85	45	89	1.09	[0.83; 1.45]	0.8%	0.0%
Toback 2022-1-Victoria	.	.	.	.			0.0%	0.0%
Toback 2022-1-Yamagata	.	.	.	.			0.0%	0.0%
Toback 2022-2-Victoria	.	.	.	.			0.0%	0.0%
Lazarus 2021-1-Victoria	.	.	.	.			0.0%	0.0%
Lazarus 2021-2-Victoria	.	.	.	.			0.0%	0.0%
Lazarus 2021-3-Victoria	.	.	.	.			0.0%	0.0%
Lazarus 2021-4-Victoria	.	.	.	.			0.0%	0.0%
Lazarus 2021-5-Victoria	.	.	.	.			0.0%	0.0%
Lazarus 2021-6-Victoria	.	.	.	.			0.0%	0.0%
Lazarus 2021-1-Yamagata	.	.	.	.			0.0%	0.0%
Lazarus 2021-2-Yamagata	.	.	.	.			0.0%	0.0%
Lazarus 2021-5-Yamagata	.	.	.	.			0.0%	0.0%
Lazarus 2021-6-Yamagata	.	.	.	.			0.0%	0.0%
Zimmermann 2013-Brisbane	.	.	.	.			0.0%	0.0%
Frenck 2012-Malaysia	.	.	.	.			0.0%	0.0%
**Common effect model**		**7433**		**7374**	**1.00**	**[0.98; 1.02]**	**100.0%**	**--**
**Random effects model**					**1.00**	**[0.99; 1.01]**	**--**	**100.0%**

Heterogeneity: I^2 = 11%, τ^2 < 0.0001, p = 0.30

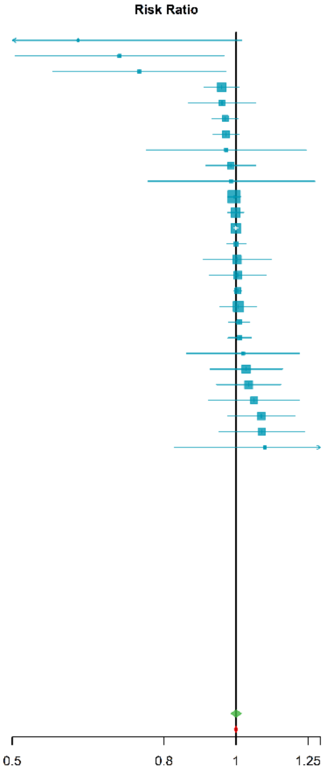

eFigure 12. Immunogenicity of concomitant vaccination vs sequential vaccination for seasonal influenza B strain GMT group in the meta-analysis

Study	Concomitant Total	Concomitant Mean	Concomitant SD	Sequential Total	Sequential Mean	Sequential SD	ROM	95%-CI	Weight (common)	Weight (random)
Toback 2022-2-Victoria	16	11.80	8.95	13.0	21.90	23.75	0.54	[0.27; 1.08]	0.0%	0.1%
Ortiz 2022-Victoria	55	46.10	14.80	52.0	61.80	18.45	0.75	[0.66; 0.84]	0.1%	1.7%
Frenck 2012-Malaysia	530	67.70	7.40	531.0	77.80	8.30	0.87	[0.86; 0.88]	5.6%	3.2%
Kerzner 2007-Shanghai	363	119.80	16.15	363.0	136.50	17.10	0.88	[0.86; 0.89]	2.7%	3.1%
Ortiz 2022-Yamagata	59	20.80	6.60	53.0	23.05	7.30	0.90	[0.80; 1.01]	0.1%	1.7%
Izikson 2022-Yamagata	96	429.00	87.50	86.0	471.00	105.00	0.91	[0.86; 0.97]	0.2%	2.6%
Schwarz 2017-Texas	384	170.20	14.75	394.0	181.60	15.55	0.94	[0.93; 0.95]	6.5%	3.2%
Weston 2012-1	711	210.10	16.30	675.0	222.80	17.60	0.94	[0.94; 0.95]	14.0%	3.2%
Lazarus 2021-1-Yamagata	61	3.60	0.40	64.0	3.80	0.40	0.95	[0.91; 0.98]	0.7%	2.9%
Herbinger 2014-Malaysia	189	110.00	28.00	181.0	116.00	30.00	0.95	[0.90; 1.00]	0.3%	2.7%
Wang 2022-Victoria	234	95.60	14.55	228.0	100.80	15.70	0.95	[0.92; 0.98]	1.2%	3.1%
Wang 2022-Yamagata	234	154.00	20.75	228.0	161.70	20.35	0.95	[0.93; 0.98]	1.7%	3.1%
Severance 2022-Victoria	576	35.58	3.40	567.0	37.13	3.60	0.96	[0.95; 0.97]	7.6%	3.2%
Song 2017-Massachusetts	373	45.00	3.75	382.0	46.70	3.75	0.96	[0.95; 0.97]	7.0%	3.2%
Lazarus 2021-1-Victoria	61	2.70	0.40	64.0	2.80	0.40	0.96	[0.92; 1.01]	0.4%	2.7%
Izikson 2022-Victoria	96	377.00	56.50	86.0	390.00	69.00	0.97	[0.92; 1.01]	0.4%	2.8%
Lazarus 2021-2-Yamagata	68	3.80	0.40	70.0	3.90	0.50	0.97	[0.94; 1.01]	0.6%	2.9%
Sadoff 2021-Brisbane	85	39.00	8.00	89.0	40.00	10.00	0.98	[0.91; 1.04]	0.2%	2.5%
Song-2 2015-Massachusetts	55	59.20	13.40	56.0	60.10	12.50	0.99	[0.91; 1.07]	0.1%	2.2%
Zimmermann 2013-Brisbane	463	305.00	32.50	448.0	308.00	34.00	0.99	[0.98; 1.00]	4.8%	3.2%
Toback 2022-1-Yamagata	201	38.80	7.35	201.0	39.00	6.30	0.99	[0.96; 1.03]	0.8%	3.0%
Sadoff 2021-Phuket?	85	35.00	7.50	89.0	35.00	9.50	1.00	[0.93; 1.08]	0.2%	2.4%
Schwarz 2011	548	90.40	9.30	546.0	88.50	9.15	1.02	[1.01; 1.03]	6.4%	3.2%
Schwarz 2017-Brisbane	384	423.50	32.75	394.0	413.90	31.70	1.02	[1.01; 1.03]	8.1%	3.2%
Severance 2022-Yamagata	576	33.82	3.00	567.0	33.03	3.10	1.02	[1.01; 1.03]	8.5%	3.2%
Toback 2022-1-Victoria	201	9.70	2.00	201.0	9.40	1.75	1.03	[0.99; 1.07]	0.6%	2.9%
Weston 2012-2	108	221.20	40.55	103.0	214.30	43.20	1.03	[0.98; 1.09]	0.4%	2.7%
Lazarus 2021-2-Victoria	68	2.50	2.30	70.0	2.40	0.30	1.04	[0.84; 1.30]	0.0%	0.8%
Lazarus 2021-5-Yamagata	63	4.90	0.60	64.0	4.70	0.60	1.04	[1.00; 1.09]	0.5%	2.9%
Lazarus 2021-4-Victoria	41	2.40	0.30	37.0	2.30	0.30	1.04	[0.99; 1.10]	0.3%	2.6%
Thompson 2019-Massachusetts	427	45.00	4.65	430.0	43.00	4.65	1.05	[1.03; 1.06]	4.7%	3.2%
Levin 2018-Victoria	409	33.90	3.60	405.0	32.30	3.45	1.05	[1.03; 1.07]	4.4%	3.2%
Levin 2018-Yamagata	409	46.70	5.00	405.0	43.90	4.45	1.06	[1.05; 1.08]	4.6%	3.2%
Thompson 2019-Brisbane	427	28.00	2.85	430.0	26.00	2.50	1.08	[1.06; 1.09]	5.4%	3.2%
Lazarus 2021-3-Victoria	73	2.30	0.25	71.0	2.10	0.25	1.10	[1.06; 1.14]	0.7%	2.9%
Lazarus 2021-6-Victoria	29	3.30	0.75	28.0	3.00	0.55	1.10	[0.99; 1.22]	0.1%	1.8%
Lazarus 2021-6-Yamagata	29	5.60	0.90	28.0	5.00	0.85	1.12	[1.03; 1.22]	0.1%	2.2%
Lazarus 2021-5-Victoria	63	3.70	0.50	0.5	3.30	0.50	1.12	[0.74; 1.71]	0.0%	0.2%
Anyinam 2017-Yamagata	.	.	.	.	.	.			0.0%	0.0%
Anyinam 2017-Victoria	.	.	.	.	.	.			0.0%	0.0%
Nakashima 2018	.	.	.	.	.	.			0.0%	0.0%
**Common effect model**	**8850**			**8699.5**			**0.98**	**[0.98; 0.99]**	**100.0%**	--
**Random effects model**							**0.99**	**[0.96; 1.01]**	**--**	**100.0%**

Heterogeneity: $I^2 = 97\%$, $\tau^2 = 0.0039$, $p < 0.01$

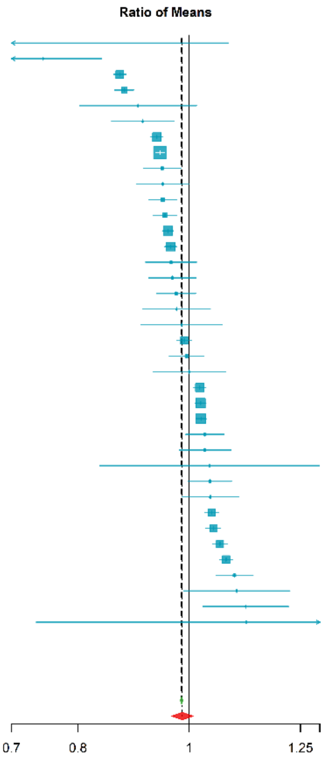

eFigure 13. Adverse events incidence of concomitant vaccination vs sequential vaccination for SARS-COV-2 fever group in the meta-analysis

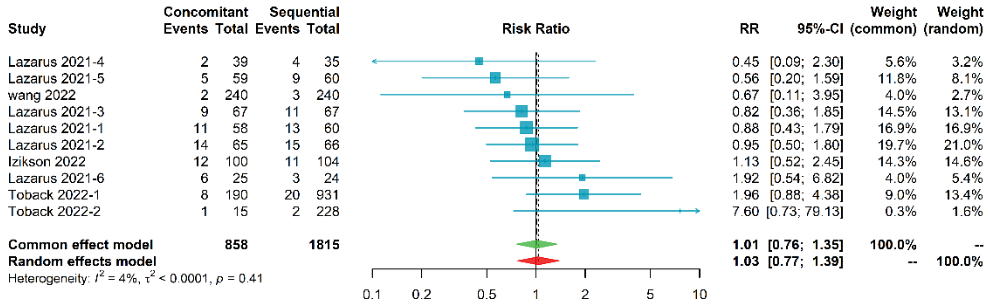

eFigure 14. Adverse events incidence of concomitant vaccination vs sequential vaccination for SARS-COV-2 >= Grade-3 fever group in the meta-analysis

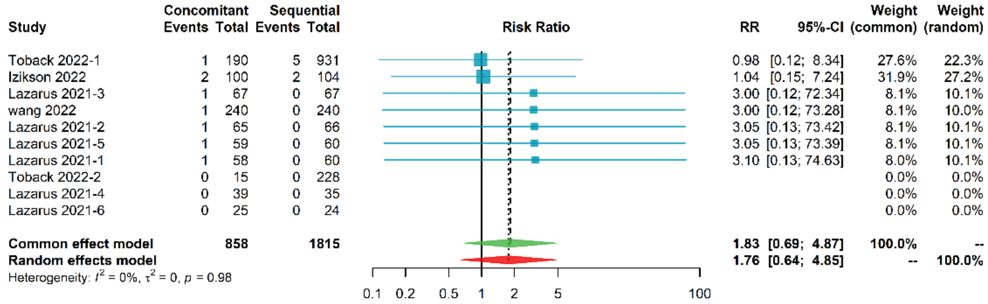

eFigure 15. Adverse events incidence of concomitant vaccination vs sequential vaccination for SARS-COV-2 fatigue group in the meta-analysis

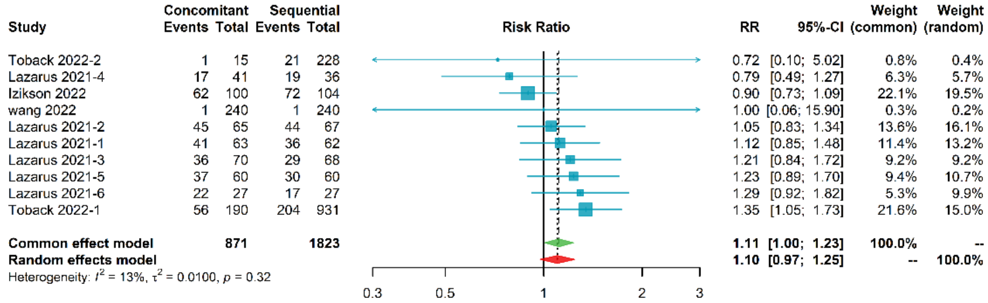

eFigure 16. Adverse events incidence of concomitant vaccination vs sequential vaccination for SARS-COV-2 >= Grade-3 fatigue group in the meta-analysis

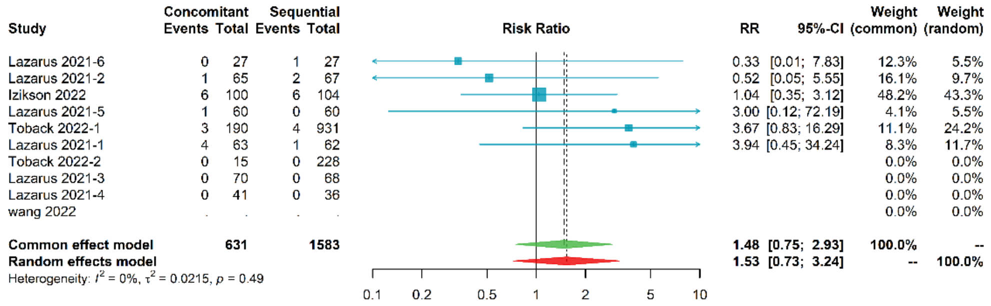

eFigure 17. Adverse events incidence of concomitant vaccination vs sequential vaccination for SARS-COV-2 headache group in the meta-analysis

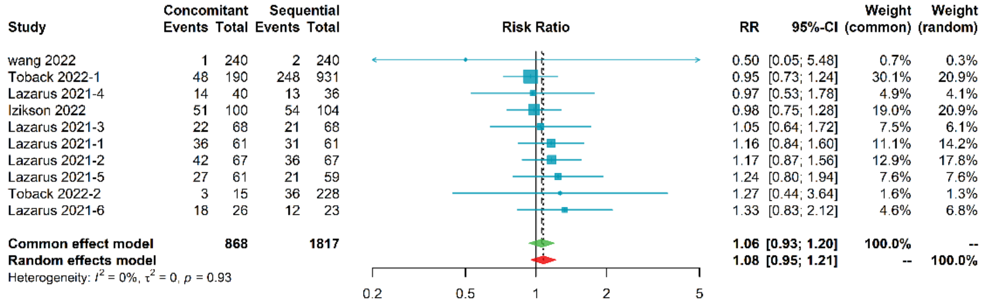

eFigure 18. Adverse events incidence of concomitant vaccination vs sequential vaccination for SARS-COV-2 >= Grade-3 headache group in the meta-analysis

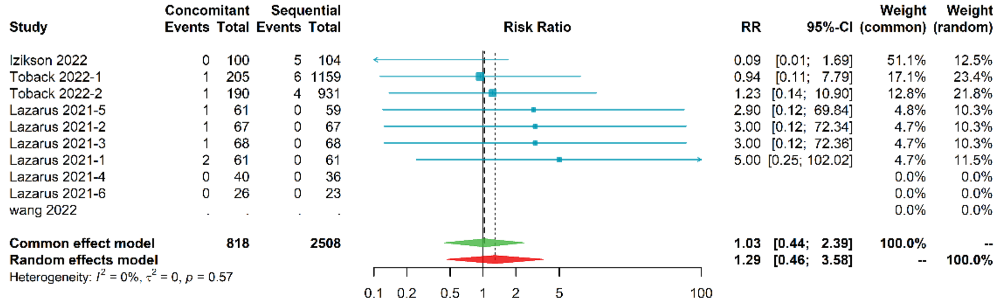

eFigure 19. Adverse events incidence of concomitant vaccination vs sequential vaccination for SARS-COV-2 malaise group in the meta-analysis

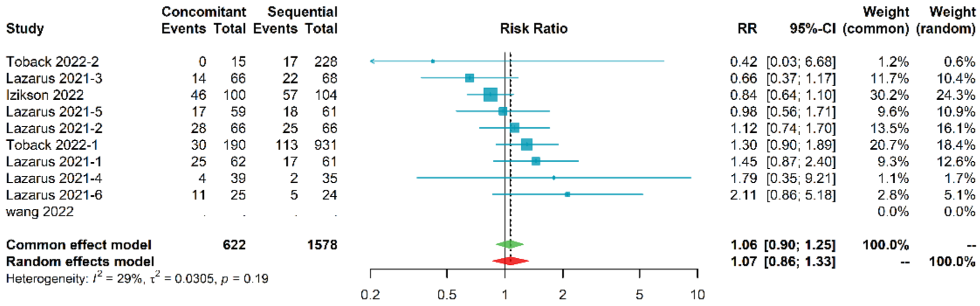

eFigure 20. Adverse events incidence of concomitant vaccination vs sequential vaccination for SARS-COV-2 >= Grade-3 malaise group in the meta-analysis

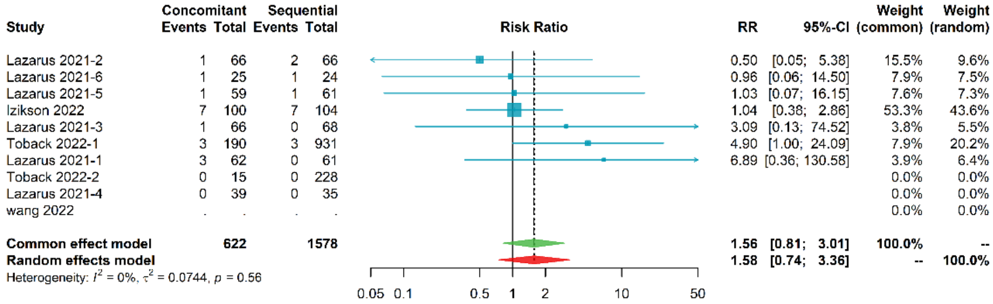

eFigure 21. Adverse events incidence of concomitant vaccination vs sequential vaccination for SARS-COV-2 muscle pain group in the meta-analysis

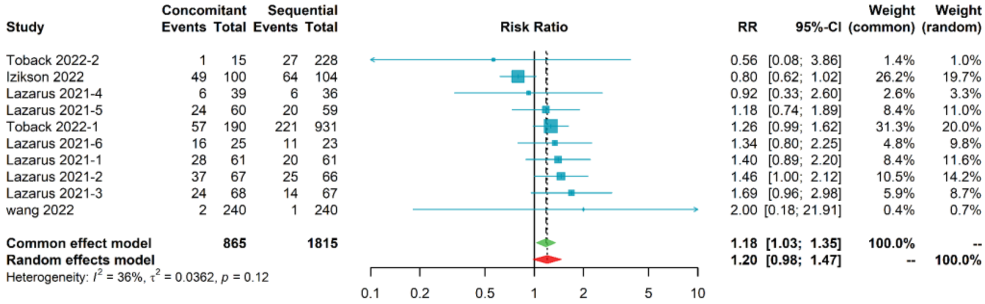

eFigure 22. Adverse events incidence of concomitant vaccination vs sequential vaccination for SARS-COV-2 >= Grade-3 muscle pain group in the meta-analysis

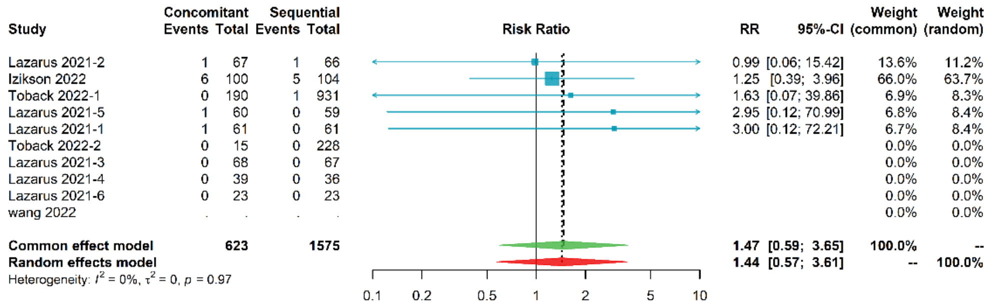

eFigure 23. Adverse events incidence of concomitant vaccination vs sequential vaccination for SARS-COV-2 gastrointestinal symptom group in the meta-analysis

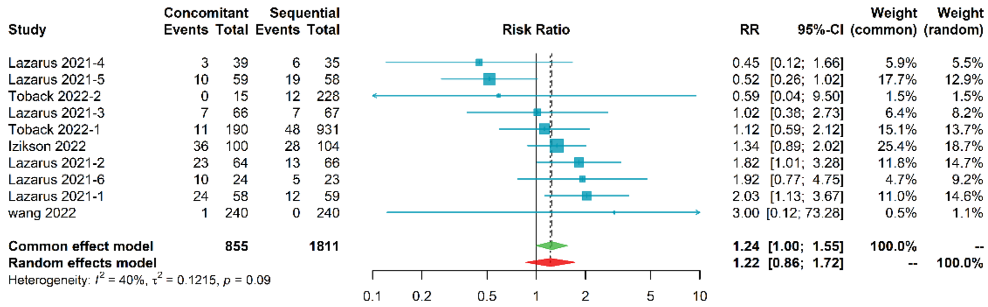

eFigure 24. Adverse events incidence of concomitant vaccination vs sequential vaccination for SARS-COV-2 local pain group in the meta-analysis

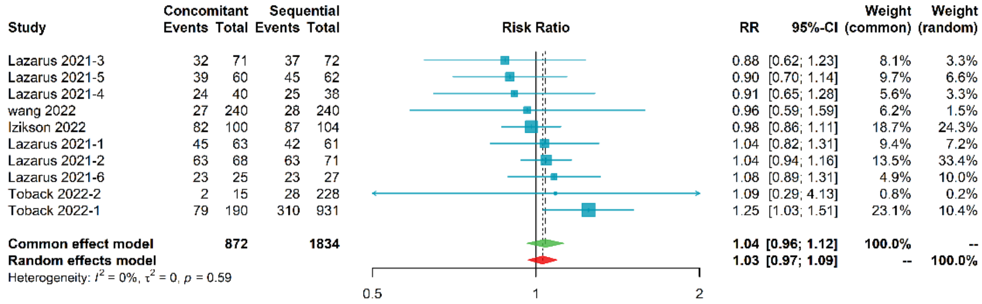

eFigure 25. Adverse events incidence of concomitant vaccination vs sequential vaccination for SARS-COV-2 >= Grade-3 local pain group in the meta-analysis

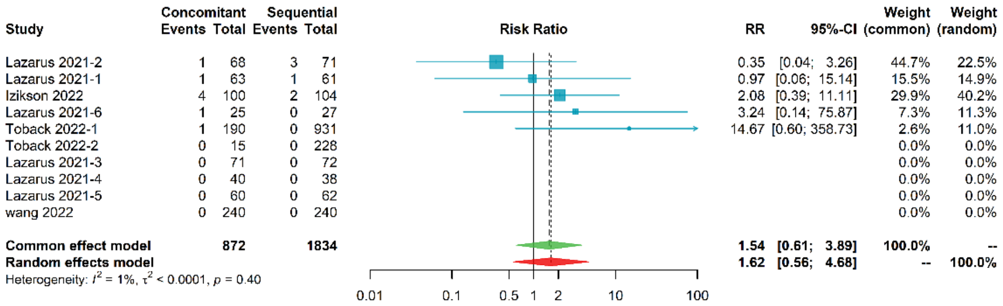

eFigure 26. Adverse events incidence of concomitant vaccination vs sequential vaccination for SARS-COV-2 chills group in the meta-analysis

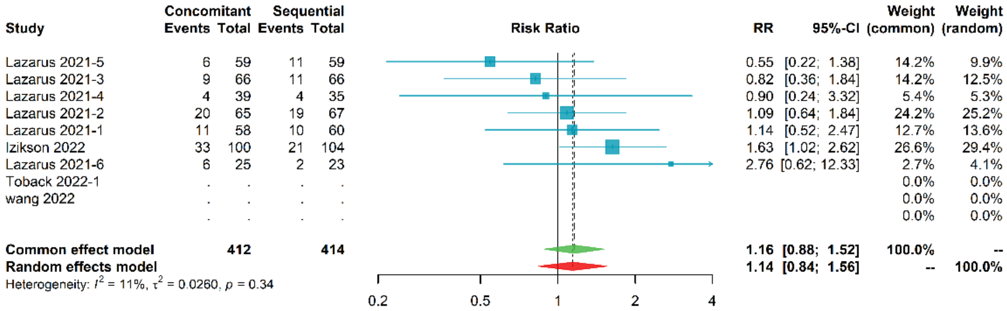

eFigure 27. Adverse events incidence of concomitant vaccination vs sequential vaccination for SARS-COV-2 tenderness group in the meta-analysis

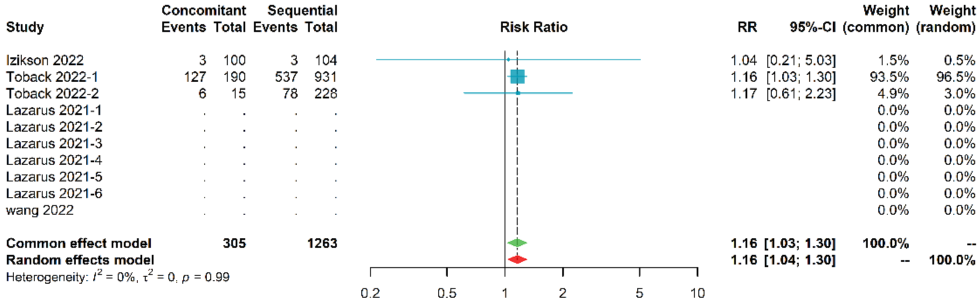

eFigure 28. Adverse events incidence of concomitant vaccination vs sequential vaccination for SARS-COV-2 erythema group in the meta-analysis

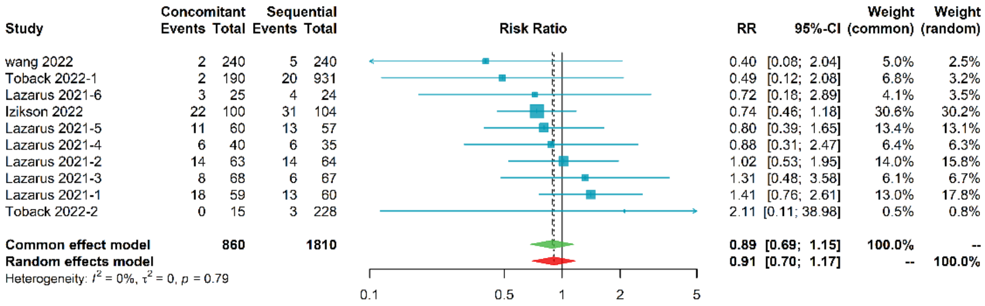

eFigure 29. Adverse events incidence of concomitant vaccination vs sequential vaccination for SARS-COV-2 swelling group in the meta-analysis

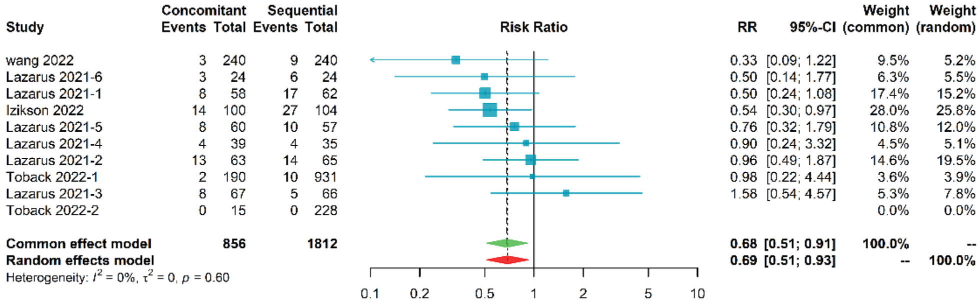

eFigure 30. Adverse events incidence of concomitant vaccination vs sequential vaccination for SARS-COV-2 induration group in the meta-analysis

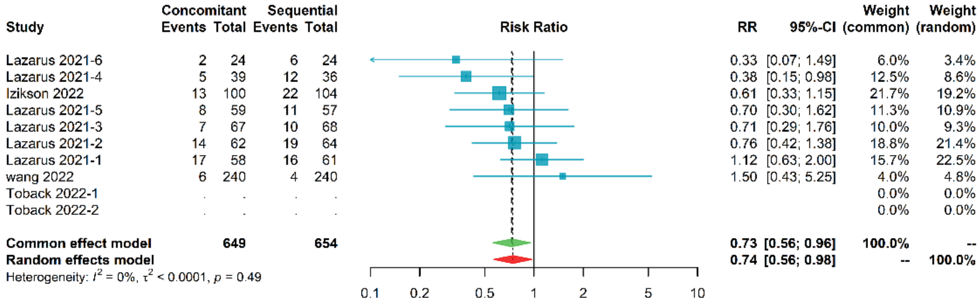

eFigure 31. Adverse events incidence of concomitant vaccination vs sequential vaccination for seasonal influenza vaccine fever group in the meta-analysis

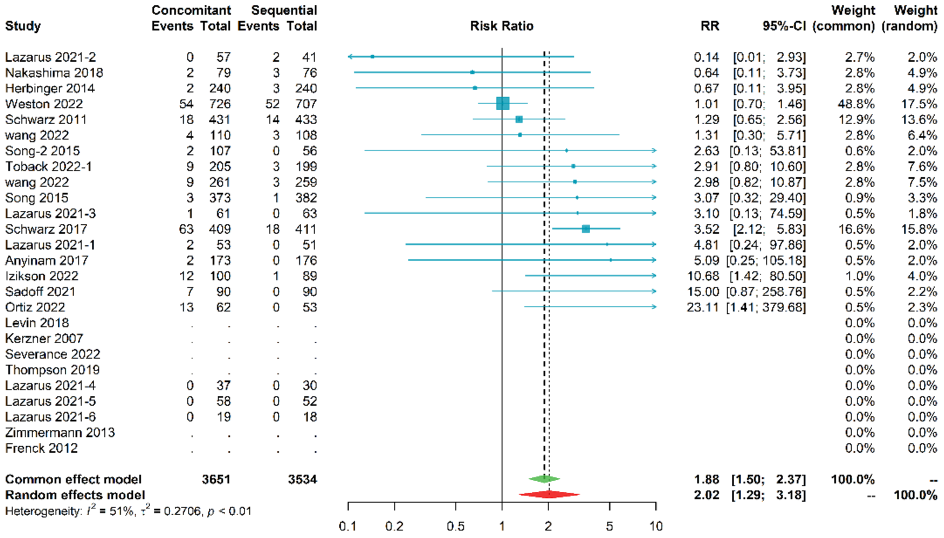

eFigure 32. Adverse events incidence of concomitant vaccination vs sequential vaccination for seasonal influenza vaccine fatigue group in the meta-analysis

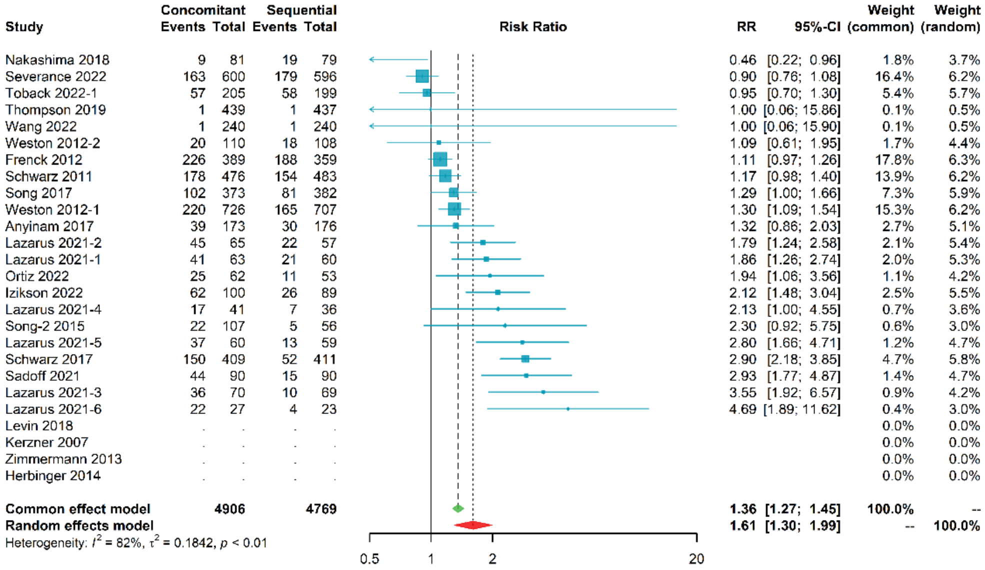

eFigure 33. Adverse events incidence of concomitant vaccination vs sequential vaccination for seasonal influenza vaccine >= Grade-3 fatigue group in the meta-analysis

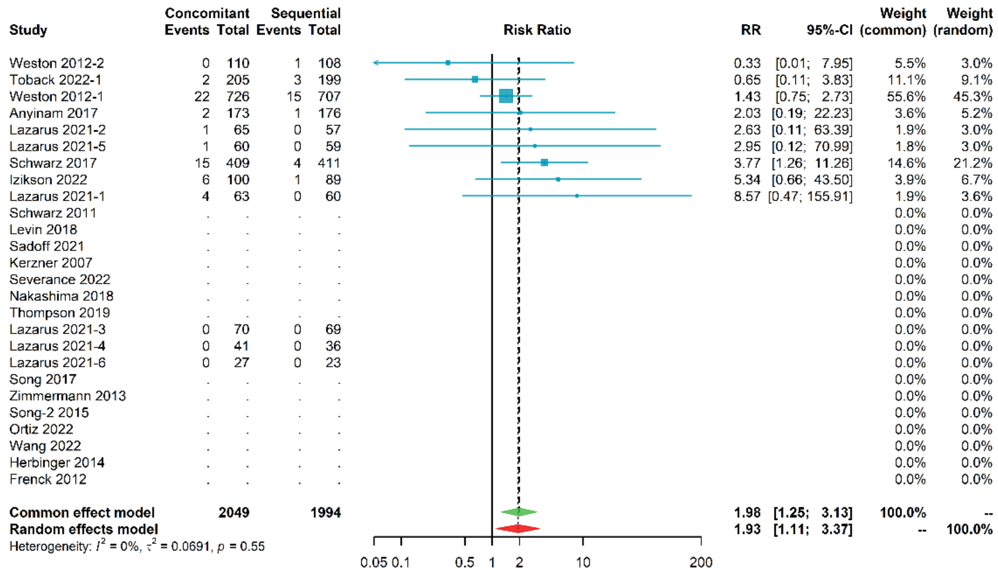

eFigure 34. Adverse events incidence of concomitant vaccination vs sequential vaccination for seasonal influenza vaccine headache group in the meta-analysis

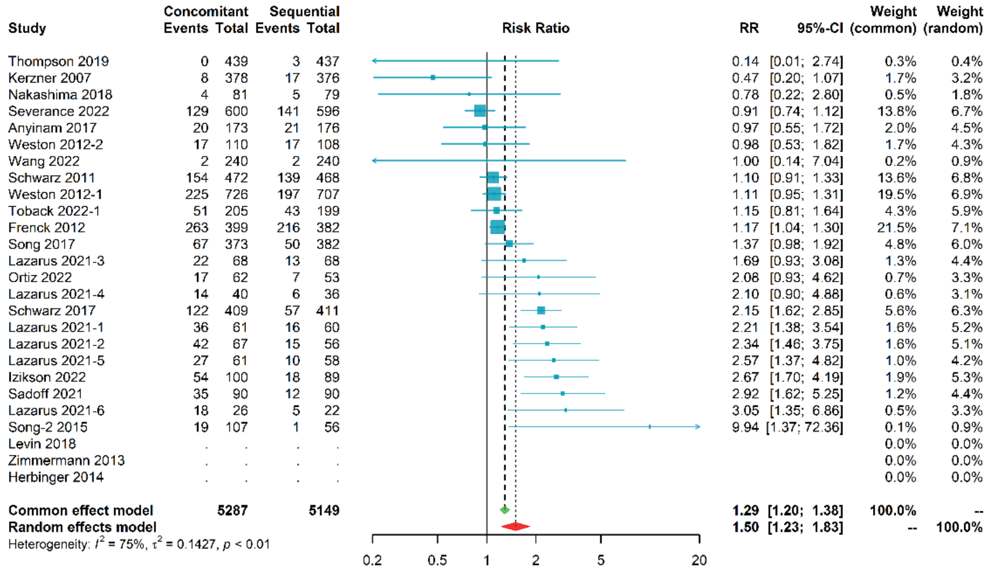

eFigure 35. Adverse events incidence of concomitant vaccination vs sequential vaccination for seasonal influenza vaccine >= Grade-3 headache group in the meta-analysis

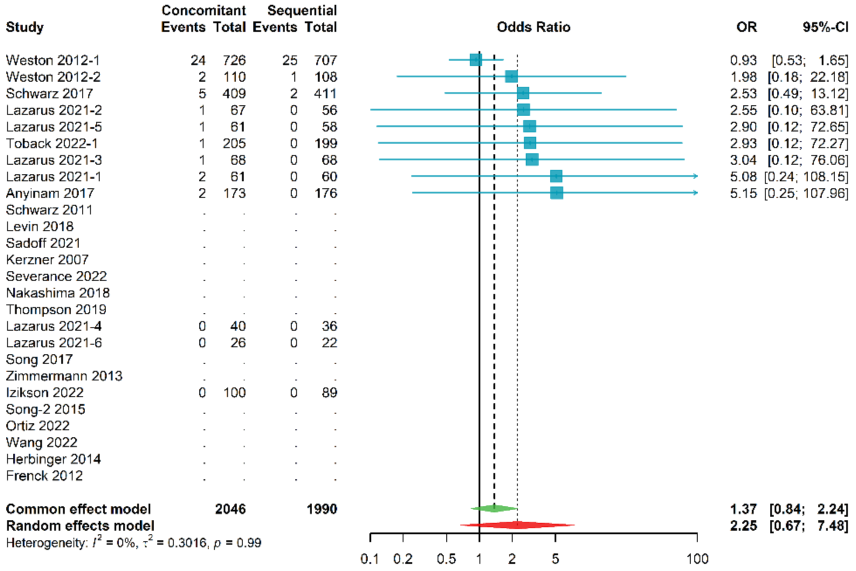

eFigure 36. Adverse events incidence of concomitant vaccination vs sequential vaccination for seasonal influenza vaccine chills group in the meta-analysis

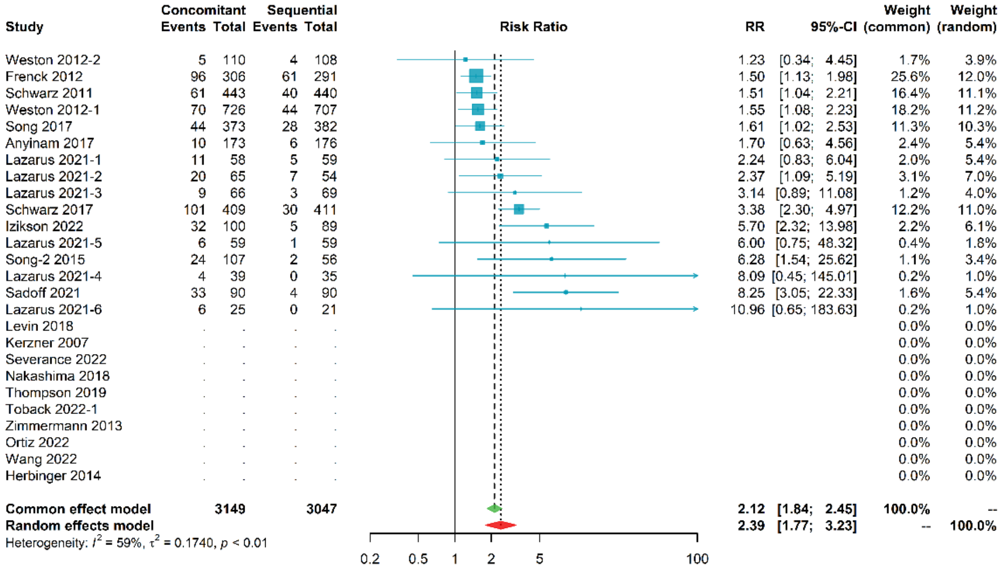

eFigure 37. Adverse events incidence of concomitant vaccination vs sequential vaccination for seasonal influenza vaccine >= Grade-3 chills group in the meta-analysis

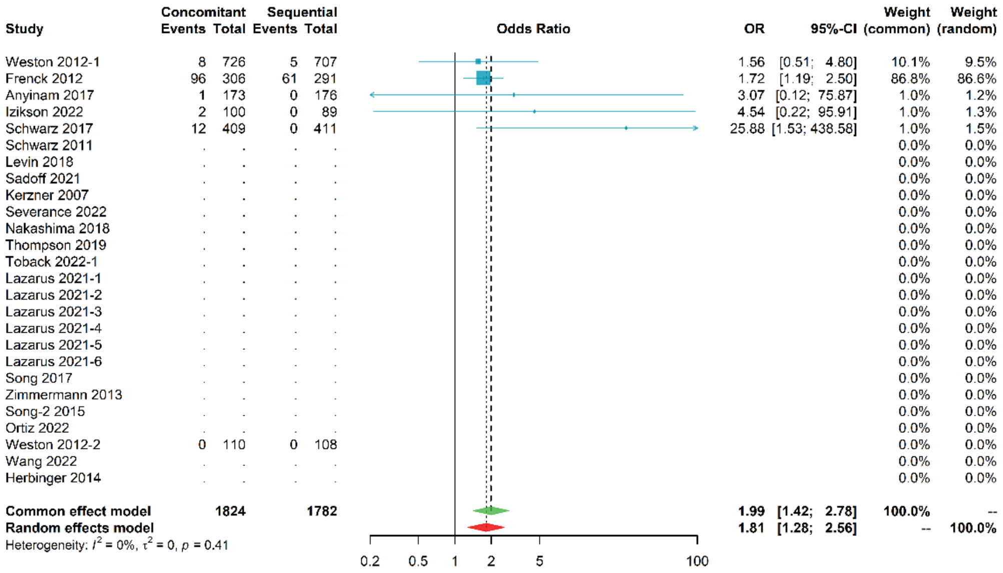

eFigure 38. Adverse events incidence of concomitant vaccination vs sequential vaccination for seasonal influenza vaccine erythra group in the meta-analysis

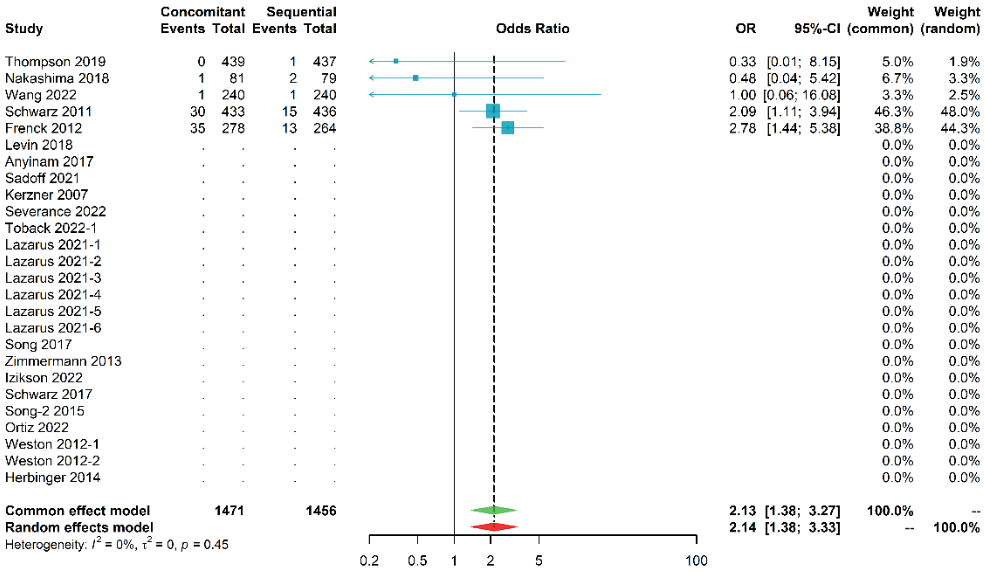

eFigure 39. Adverse events incidence of concomitant vaccination vs sequential vaccination for seasonal influenza vaccine gastrointestinal symptom group in the meta-analysis

Study	Concomitant Events	Concomitant Total	Sequential Events	Sequential Total	RR	95%-CI	Weight (common)	Weight (random)
Wang 2022	1	240	3	240	0.33	[0.03; 3.18]	0.8%	1.1%
Thompson 2019	2	439	4	437	0.50	[0.09; 2.70]	1.1%	1.9%
Anyinam 2017	12	173	16	176	0.76	[0.37; 1.57]	4.2%	6.2%
Toback 2022-1	11	205	13	199	0.82	[0.38; 1.79]	3.5%	5.7%
Lazarus 2021-4	2	39	2	35	0.90	[0.13; 6.04]	0.6%	1.5%
Schwarz 2011	89	450	81	452	1.10	[0.84; 1.45]	21.4%	11.2%
Weston 2012-1	121	726	101	707	1.17	[0.91; 1.49]	27.1%	11.5%
Lazarus 2021-5	10	59	8	58	1.23	[0.52; 2.89]	2.1%	5.1%
Frenck 2012	95	315	68	301	1.33	[1.02; 1.75]	18.4%	11.2%
Weston 2012-2	18	110	12	108	1.47	[0.75; 2.91]	3.2%	6.6%
Ortiz 2022	2	62	1	53	1.71	[0.16; 18.33]	0.3%	1.0%
Lazarus 2021-6	10	24	5	21	1.75	[0.71; 4.30]	1.4%	4.8%
Schwarz 2017	57	409	32	411	1.79	[1.19; 2.70]	8.5%	9.5%
Lazarus 2021-1	24	58	10	59	2.44	[1.28; 4.64]	2.6%	6.9%
Lazarus 2021-3	7	66	2	67	3.55	[0.77; 16.48]	0.5%	2.2%
Lazarus 2021-2	23	64	5	53	3.81	[1.55; 9.33]	1.5%	4.8%
Izikson 2022	36	100	8	89	4.00	[1.97; 8.15]	2.2%	6.3%
Sadoff 2021	13	90	2	90	6.50	[1.51; 27.98]	0.5%	2.4%
Levin 2018	.	.	.	.			0.0%	0.0%
Kerzner 2007	.	.	.	.			0.0%	0.0%
Severance 2022	.	.	.	.			0.0%	0.0%
Nakashima 2018	.	.	.	.			0.0%	0.0%
Song 2017	.	.	.	.			0.0%	0.0%
Zimmermann 2013	.	.	.	.			0.0%	0.0%
Song-2 2015	.	.	.	.			0.0%	0.0%
Herbinger 2014	.	.	.	.			0.0%	0.0%
**Common effect model**		**3629**		**3556**	**1.39**	**[1.23; 1.57]**	**100.0%**	--
**Random effects model**					**1.52**	**[1.18; 1.95]**	--	**100.0%**

Heterogeneity: $I^2 = 53\%$, $\tau^2 = 0.1265$, $p < 0.01$

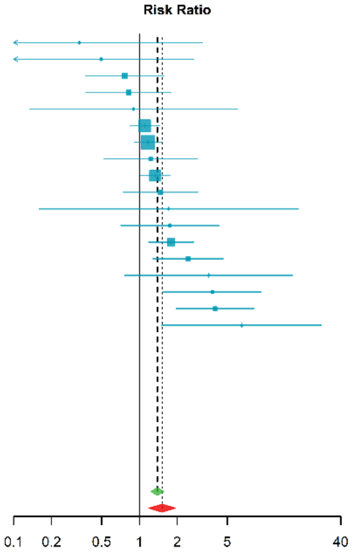

eFigure 40. Adverse events incidence of concomitant vaccination vs sequential vaccination for seasonal influenza vaccine >= Grade-3 gastrointestinal symptom group in the meta-analysis

Study	Concomitant Events	Concomitant Total	Sequential Events	Sequential Total	RR	95%-CI	Weight (common)	Weight (random)
Lazarus 2021-5	0	59	2	58	0.20	[0.01; 4.01]	12.7%	4.0%
Lazarus 2021-2	0	64	1	53	0.28	[0.01; 6.65]	8.3%	3.6%
Anyinam 2017	1	173	1	176	1.02	[0.06; 16.14]	5.0%	4.8%
Weston 2012-1	16	726	11	707	1.42	[0.66; 3.03]	56.2%	63.2%
Weston 2012-2	2	110	1	108	1.96	[0.18; 21.34]	5.1%	6.4%
Schwarz 2017	5	409	2	411	2.51	[0.49; 12.87]	10.1%	13.7%
Izikson 2022	3	100	0	89	6.23	[0.33; 119.04]	2.7%	4.2%
Schwarz 2011	.	.	.	.			0.0%	0.0%
Levin 2018	.	.	.	.			0.0%	0.0%
Sadoff 2021	.	.	.	.			0.0%	0.0%
Kerzner 2007	.	.	.	.			0.0%	0.0%
Severance 2022	.	.	.	.			0.0%	0.0%
Nakashima 2018	.	.	.	.			0.0%	0.0%
Thompson 2019	.	.	.	.			0.0%	0.0%
Toback 2022-1	0	205	0	199			0.0%	0.0%
Lazarus 2021-1	0	58	0	59			0.0%	0.0%
Lazarus 2021-3	0	66	0	67			0.0%	0.0%
Lazarus 2021-4	0	39	0	35			0.0%	0.0%
Lazarus 2021-6	0	24	0	21			0.0%	0.0%
Song 2017	.	.	.	.			0.0%	0.0%
Zimmermann 2013	.	.	.	.			0.0%	0.0%
Song-2 2015	.	.	.	.			0.0%	0.0%
Ortiz 2022	.	.	.	.			0.0%	0.0%
Wang 2022	.	.	.	.			0.0%	0.0%
Herbinger 2014	.	.	.	.			0.0%	0.0%
Frenck 2012	.	.	.	.			0.0%	0.0%
**Common effect model**		**2033**		**1983**	**1.41**	**[0.80; 2.50]**	**100.0%**	--
**Random effects model**					**1.43**	**[0.78; 2.61]**	--	**100.0%**

Heterogeneity: $I^2 = 0\%$, $\tau^2 = 0$, $p = 0.65$

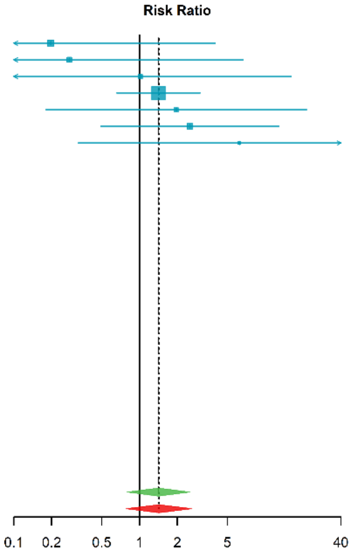

eFigure 41. Adverse events incidence of concomitant vaccination vs sequential vaccination for seasonal influenza vaccine arthralgia group in the meta-analysis

Study	Concomitant Events	Concomitant Total	Sequential Events	Sequential Total	RR	95%-CI	Weight (common)	Weight (random)
Kerzner 2007	56	600	69	596	0.81	[0.58; 1.13]	15.9%	8.0%
Severance 2022	11	81	11	79	0.98	[0.45; 2.12]	2.6%	5.0%
Weston 2012-2	14	110	13	108	1.06	[0.52; 2.14]	3.0%	5.4%
Song 2017	27	373	25	382	1.11	[0.65; 1.87]	5.7%	6.6%
Schwarz 2011	73	452	59	451	1.23	[0.90; 1.70]	13.6%	8.1%
Frenck 2012	102	309	73	296	1.34	[1.04; 1.73]	17.2%	8.5%
Song-2 2015	11	107	4	56	1.44	[0.48; 4.31]	1.2%	3.4%
Anyinam 2017	16	173	11	176	1.48	[0.71; 3.10]	2.5%	5.2%
Weston 2012-1	137	726	87	707	1.53	[1.20; 1.96]	20.3%	8.5%
Lazarus 2021-6	8	26	4	22	1.69	[0.59; 4.87]	1.0%	3.6%
Ortiz 2022	8	62	4	53	1.71	[0.55; 5.36]	1.0%	3.2%
Schwarz 2017	89	409	37	411	2.42	[1.69; 3.46]	8.5%	7.8%
Lazarus 2021-4	3	39	1	35	2.69	[0.29; 24.71]	0.2%	1.2%
Lazarus 2021-3	14	68	5	67	2.76	[1.05; 7.23]	1.2%	4.0%
Izikson 2022	25	100	8	89	2.78	[1.32; 5.85]	1.9%	5.2%
Lazarus 2021-1	18	60	5	59	3.54	[1.41; 8.91]	1.2%	4.2%
Lazarus 2021-2	19	65	4	53	3.87	[1.40; 10.69]	1.0%	3.7%
Toback 2022-1	15	205	3	199	4.85	[1.43; 16.51]	0.7%	2.9%
Sadoff 2021	32	90	5	90	6.40	[2.61; 15.68]	1.2%	4.3%
Lazarus 2021-5	9	59	1	58	8.85	[1.16; 67.63]	0.2%	1.3%
Levin 2018	.	.	.	.			0.0%	0.0%
Nakashima 2018	.	.	.	.			0.0%	0.0%
Thompson 2019	0	439	0	437			0.0%	0.0%
Zimmermann 2013	.	.	.	.			0.0%	0.0%
Wang 2022	.	.	.	.			0.0%	0.0%
Herbinger 2014	.	.	.	.			0.0%	0.0%
**Common effect model**		**4553**		**4424**	**1.55**	**[1.39; 1.73]**	**100.0%**	**--**
**Random effects model**					**1.77**	**[1.37; 2.28]**	**--**	**100.0%**

Heterogeneity: I^2 = 64%, τ^2 = 0.1818, p < 0.01

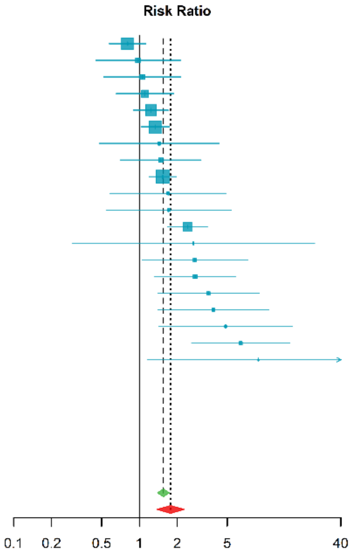

eFigure 42. Adverse events incidence of concomitant vaccination vs sequential vaccination for seasonal influenza vaccine muscle pain group in the meta-analysis

Study	Concomitant Events	Concomitant Total	Sequential Events	Sequential Total	RR	95%-CI	Weight (common)	Weight (random)
Severance 2022	142	600	127	596	1.11	[0.90; 1.37]	15.8%	8.2%
Weston 2012-1	266	726	193	707	1.34	[1.15; 1.57]	24.2%	8.8%
Levin 2018	20	173	15	176	1.36	[0.72; 2.56]	1.8%	4.0%
Toback 2022-1	58	205	40	199	1.41	[0.99; 2.00]	5.0%	6.7%
Weston 2012-2	26	110	18	108	1.42	[0.83; 2.43]	2.3%	4.8%
Song 2017	75	373	49	382	1.57	[1.13; 2.18]	6.0%	6.9%
Schwarz 2011	126	468	76	456	1.62	[1.25; 2.08]	9.5%	7.8%
Frenck 2012	252	385	123	326	1.73	[1.48; 2.03]	16.5%	8.7%
Lazarus 2021-4	6	39	3	35	1.79	[0.48; 6.64]	0.4%	1.4%
Thompson 2019	2	439	1	437	1.99	[0.18; 21.88]	0.1%	0.5%
Wang 2022	2	240	1	240	2.00	[0.18; 21.91]	0.1%	0.5%
Izikson 2022	49	100	21	89	2.08	[1.36; 3.17]	2.8%	5.9%
Song-2 2015	24	107	6	56	2.09	[0.91; 4.82]	1.0%	2.8%
Lazarus 2021-6	16	25	7	23	2.10	[1.06; 4.17]	0.9%	3.7%
Lazarus 2021-3	24	68	11	68	2.18	[1.16; 4.09]	1.4%	4.0%
Schwarz 2017	135	409	55	411	2.47	[1.86; 3.27]	6.8%	7.5%
Sadoff 2021	41	90	15	90	2.73	[1.63; 4.57]	1.9%	5.0%
Lazarus 2021-5	24	60	7	58	3.31	[1.55; 7.09]	0.9%	3.2%
Ortiz 2022	28	62	7	53	3.42	[1.63; 7.19]	0.9%	3.3%
Lazarus 2021-1	28	61	7	60	3.93	[1.86; 8.31]	0.9%	3.3%
Lazarus 2021-2	37	67	6	53	4.88	[2.23; 10.68]	0.8%	3.1%
Anyinam 2017	.	.	.	.			0.0%	0.0%
Kerzner 2007	.	.	.	.			0.0%	0.0%
Nakashima 2018	.	.	.	.			0.0%	0.0%
Zimmermann 2013	.	.	.	.			0.0%	0.0%
Herbinger 2014	.	.	.	.			0.0%	0.0%
**Common effect model**		**4807**		**4623**	**1.66**	**[1.54; 1.78]**	**100.0%**	**--**
**Random effects model**					**1.88**	**[1.59; 2.22]**	**--**	**100.0%**

Heterogeneity: I^2 = 64%, τ^2 = 0.0769, p < 0.01

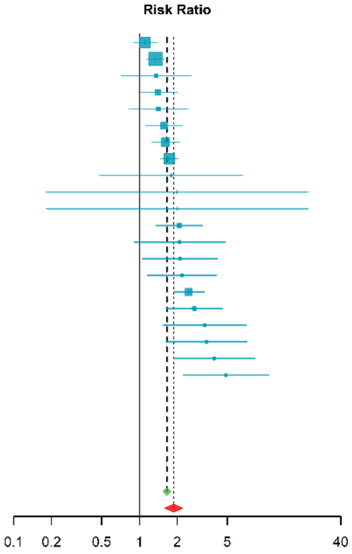

eFigure 43. Adverse events incidence of concomitant vaccination vs sequential vaccination for seasonal influenza vaccine >= Grade-3 muscle pain group in the meta-analysis

Study	Concomitant Events	Concomitant Total	Sequential Events	Sequential Total	RR	95%-CI	Weight (common)	Weight (random)
Weston 2012-1	27	726	15	707	1.75	[0.94; 3.27]	83.1%	80.5%
Lazarus 2021-2	1	67	0	53	2.38	[0.10; 57.20]	3.0%	3.1%
Lazarus 2021-5	1	60	0	58	2.90	[0.12; 69.78]	2.8%	3.1%
Lazarus 2021-1	1	61	0	60	2.95	[0.12; 71.03]	2.8%	3.1%
Anyinam 2017	1	173	0	176	3.05	[0.13; 74.40]	2.7%	3.1%
Weston 2012-2	2	110	0	108	4.91	[0.24; 101.08]	2.8%	3.4%
Izikson 2022	6	100	0	89	11.58	[0.66; 202.62]	2.9%	3.8%
Schwarz 2011	.	.	.	.			0.0%	0.0%
Levin 2018	.	.	.	.			0.0%	0.0%
Sadoff 2021	.	.	.	.			0.0%	0.0%
Kerzner 2007	.	.	.	.			0.0%	0.0%
Severance 2022	.	.	.	.			0.0%	0.0%
Nakashima 2018	.	.	.	.			0.0%	0.0%
Thompson 2019	.	.	.	.			0.0%	0.0%
Toback 2022-1	0	205	0	199			0.0%	0.0%
Lazarus 2021-3	0	68	0	68			0.0%	0.0%
Lazarus 2021-4	0	39	0	35			0.0%	0.0%
Lazarus 2021-6	0	25	0	23			0.0%	0.0%
Song 2017	.	.	.	.			0.0%	0.0%
Zimmermann 2013	.	.	.	.			0.0%	0.0%
Schwarz 2017	.	.	.	.			0.0%	0.0%
Song-2 2015	.	.	.	.			0.0%	0.0%
Ortiz 2022	.	.	.	.			0.0%	0.0%
Wang 2022	.	.	.	.			0.0%	0.0%
Herbinger 2014	.	.	.	.			0.0%	0.0%
Frenck 2012	.	.	.	.			0.0%	0.0%
**Common effect model**		**1634**		**1576**	**2.24**	**[1.30; 3.88]**	**100.0%**	--
**Random effects model**					**2.07**	**[1.18; 3.61]**	--	**100.0%**

Heterogeneity: $I^2 = 0\%$, $\tau^2 = 0$, $p = 0.91$

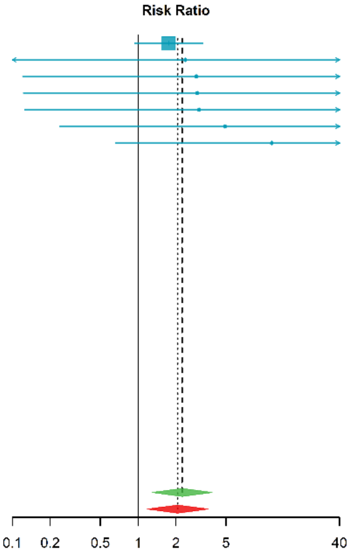

eFigure 44. Adverse events incidence of concomitant vaccination vs sequential vaccination for seasonal influenza vaccine malaise group in the meta-analysis

Study	Concomitant Events	Concomitant Total	Sequential Events	Sequential Total	RR	95%-CI	Weight (common)	Weight (random)
Toback 2022-1	30	205	33	199	0.88	[0.56; 1.39]	34.5%	16.5%
Lazarus 2021-4	4	39	4	37	0.95	[0.26; 3.52]	4.2%	5.5%
Lazarus 2021-5	17	59	9	58	1.86	[0.90; 3.82]	9.4%	11.7%
Lazarus 2021-3	14	66	7	69	2.09	[0.90; 4.85]	7.1%	9.9%
Lazarus 2021-2	28	66	10	55	2.33	[1.25; 4.37]	11.3%	13.2%
Song-2 2015	10	107	2	56	2.62	[0.59; 11.53]	2.7%	4.6%
Lazarus 2021-1	25	62	9	60	2.69	[1.37; 5.28]	9.4%	12.4%
Ortiz 2022	19	62	6	53	2.71	[1.17; 6.28]	6.7%	9.9%
Izikson 2022	46	100	13	89	3.15	[1.83; 5.43]	14.2%	14.7%
Lazarus 2021-6	11	25	0	21	19.39	[1.21; 310.30]	0.6%	1.5%
Schwarz 2011	.	.	.	.			0.0%	0.0%
Levin 2018	.	.	.	.			0.0%	0.0%
Anyinam 2017	.	.	.	.			0.0%	0.0%
Sadoff 2021	.	.	.	.			0.0%	0.0%
Kerzner 2007	.	.	.	.			0.0%	0.0%
Severance 2022	.	.	.	.			0.0%	0.0%
Nakashima 2018	.	.	.	.			0.0%	0.0%
Thompson 2019	.	.	.	.			0.0%	0.0%
Song 2017	.	.	.	.			0.0%	0.0%
Zimmermann 2013	.	.	.	.			0.0%	0.0%
Schwarz 2017	.	.	.	.			0.0%	0.0%
Weston 2012-1	.	.	.	.			0.0%	0.0%
Weston 2012-2	.	.	.	.			0.0%	0.0%
Wang 2022	.	.	.	.			0.0%	0.0%
Herbinger 2014	.	.	.	.			0.0%	0.0%
Frenck 2012	.	.	.	.			0.0%	0.0%
**Common effect model**		**791**		**697**	**1.99**	**[1.59; 2.49]**	**100.0%**	--
**Random effects model**					**2.04**	**[1.43; 2.91]**	--	**100.0%**

Heterogeneity: $I^2 = 56\%$, $\tau^2 = 0.1441$, $p = 0.02$

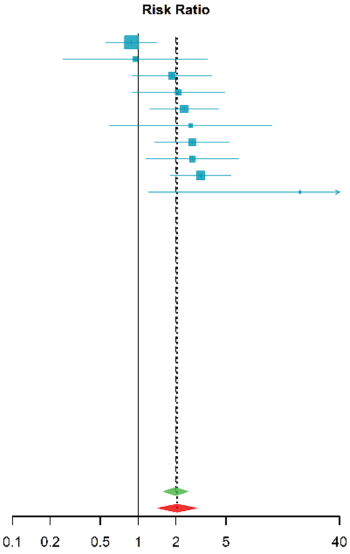

eFigure 45. Adverse events incidence of concomitant vaccination vs sequential vaccination for seasonal influenza vaccine >= Grade-3 malaise group in the meta-analysis

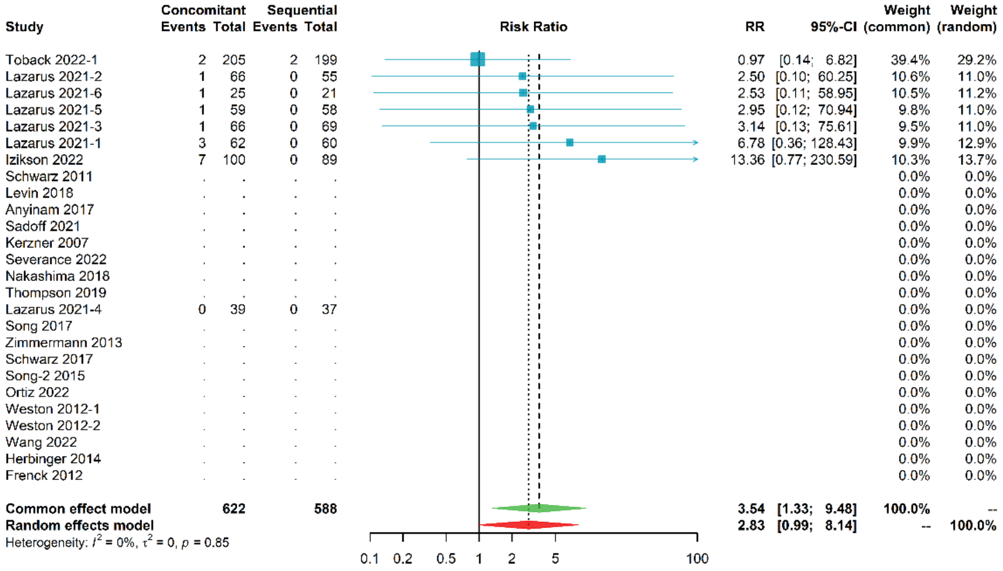

eFigure 46. Adverse events incidence of concomitant vaccination vs sequential vaccination for seasonal influenza vaccine local pain group in the meta-analysis

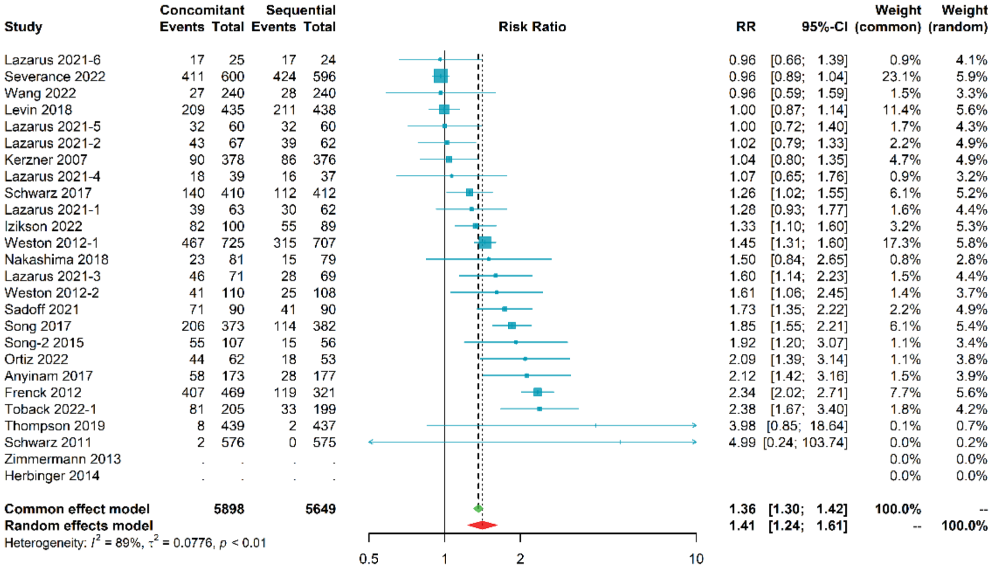

eFigure 47. Adverse events incidence of concomitant vaccination vs sequential vaccination for seasonal influenza vaccine >= Grade-3 local pain group in the meta-analysis

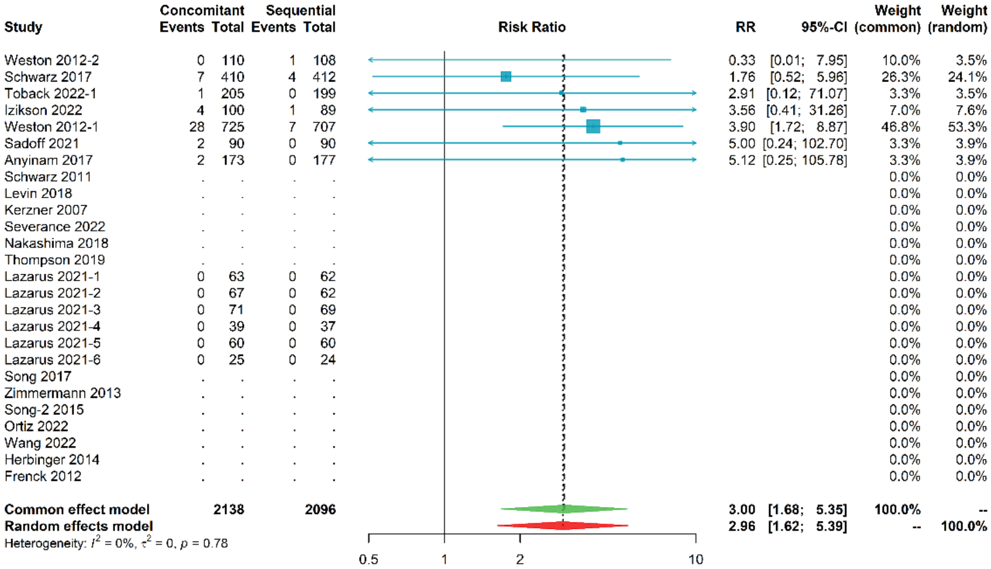

eFigure 48. Adverse events incidence of concomitant vaccination vs sequential vaccination for seasonal influenza vaccine erythema group in the meta-analysis

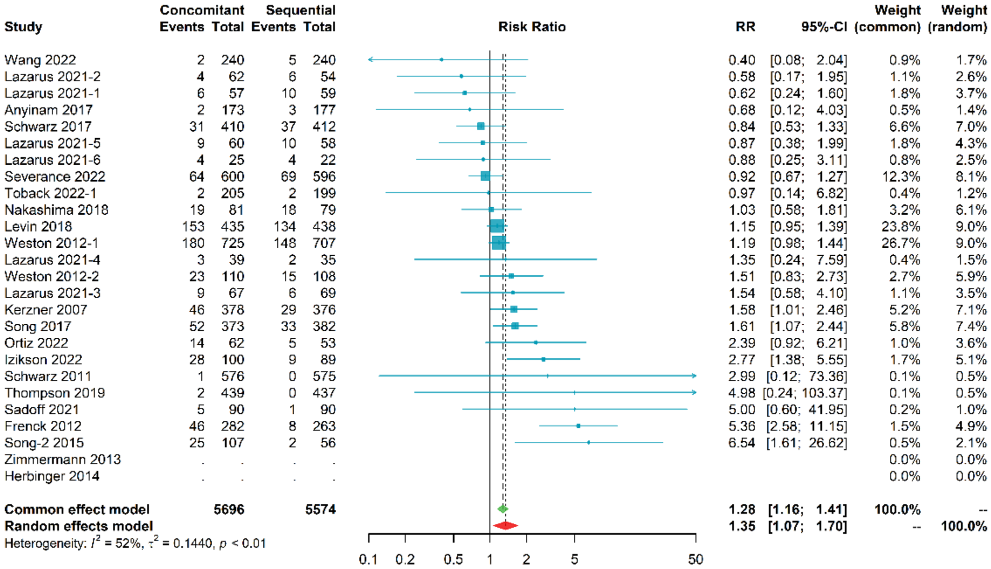

eFigure 49. Adverse events incidence of concomitant vaccination vs sequential vaccination for seasonal influenza vaccine >= Grade-3 erythema group in the meta-analysis

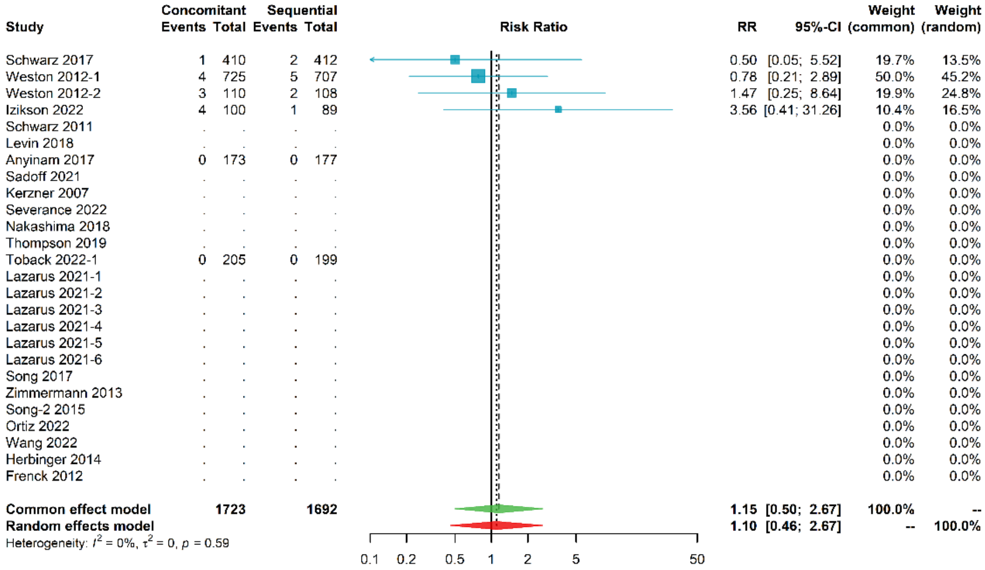

eFigure 50. Adverse events incidence of concomitant vaccination vs sequential vaccination for seasonal influenza vaccine itch group in the meta-analysis

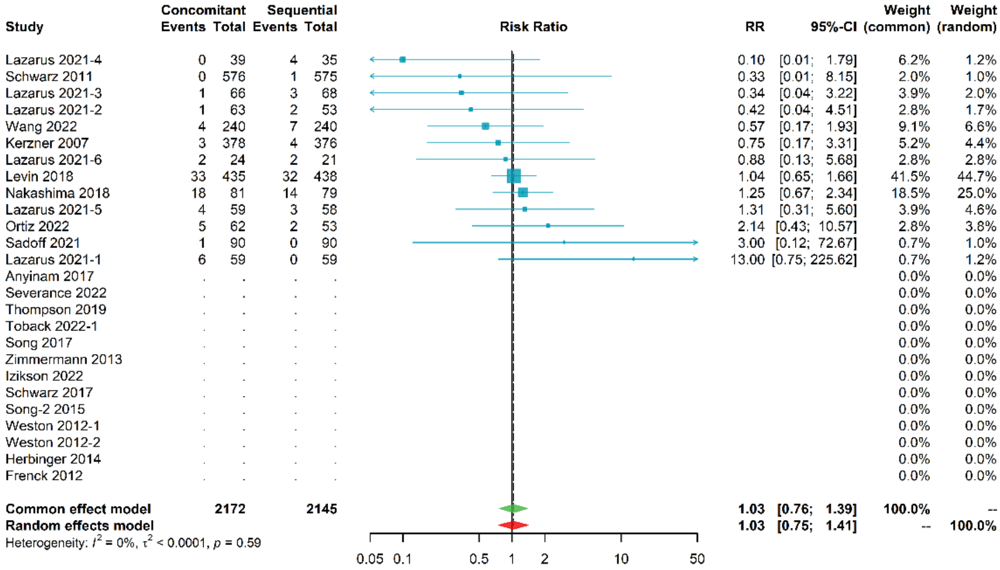

eFigure 51. Adverse events incidence of concomitant vaccination vs sequential vaccination for seasonal influenza vaccine swelling group in the meta-analysis

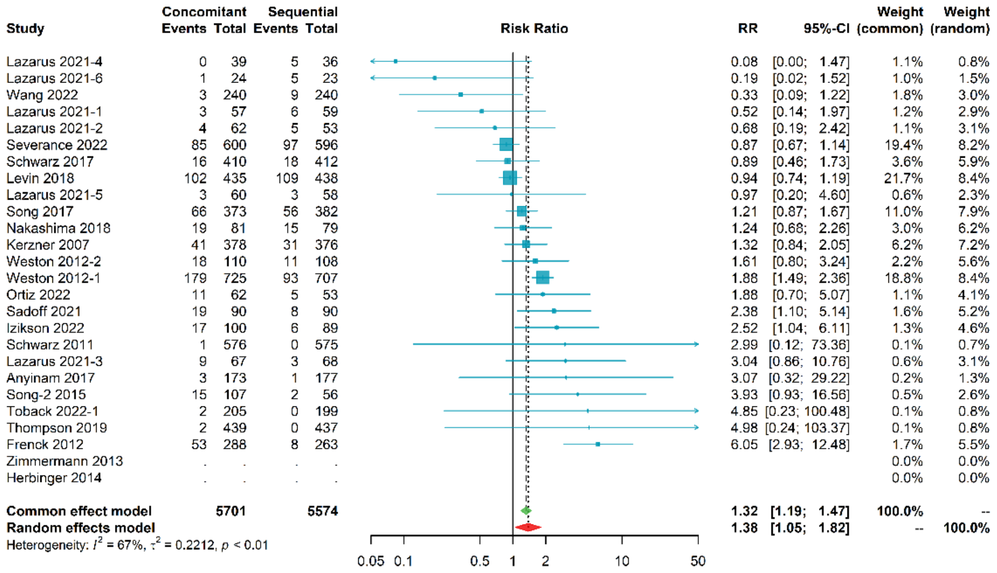

eFigure 52. Adverse events incidence of concomitant vaccination vs sequential vaccination for seasonal influenza vaccine >= Grade-3 swelling group in the meta-analysis

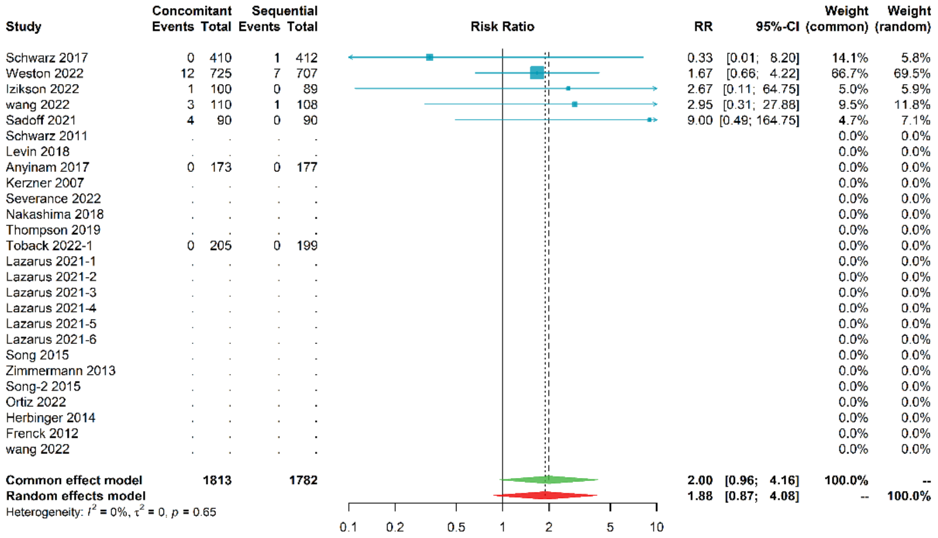

eFigure 53. Risk of Bias Assessments

Experimental	Randomization process	Deviations from intended interventions	Missing outcome data	Measurement of the outcome	Selection of the reported result	Overall		
Schwarz 2011	+	+	+	+	+	+	+	Low risk
Levin 2018	+	+	+	?	+	!	?	Some concerns
Anyinam 2017	+	?	+	+	+	!	?	High risk
Sadoff 2021	+	?	+	+	+	!		
Kerzner 2007	?	+	+	?	+	?		
Severance 2022	+	+	+	+	+	+		
Nakashima 2018	+	+	+	?	+	!		
Thompson 2019	+	+	+	+	+	+		
Toback 2022	+	+	+	+	+	+		
Lazarus 2021	+	+	+	+	?	!		
Song 2017	+	?	+	?	+	!		
Zimmermann 2013	+	+	+	+	+	+		
Izikson 2022	+	+	+	+	+	+		
Schwarz 2017	+	+	+	?	+	!		
Song 2015	?	?	+	?	+	?		
Ortiz 2022	+	?	+	?	+	?		
Weston 2009	?	+	+	?	+	?		
Weston 2009	+	?	+	?	+	?		
Frenck 2012	?	+	+	+	+	!		
Herbinger 2013	?	?	+	+	+	!		
Wang 2022	+	+	+	?	+	!		

The risk of bias for each trial was assessed using the Cochrane Handbook for Systematic Reviews of Interventions. Items were considered to have low, some concerned, or highly concerned risk of bias. We further assessed the risk of bias in the included studies in 5 categories. In the randomization pross evaluation, the risk level for 5 articles was escalated to 'some concerns' due to vague descriptions of their random grouping methods, which were only broadly mentioned without specific details. In deviations from intended interventions, 7 group studies were classified under 'some concerns' because the randomized allocation was not adequately concealed, potentially affecting the study integrity. In the missing outcome data section, the adopted articles had a good performance in this section. In the measurement of the outcome score, several articles did not describe the blinding for outcome assessors. The outcome assessors potentially had some measurement bias, for this reason, generating high-risk assessments. In the selection of the reported result, we upgraded the risk rating of the Lazarus 2021 group because it modified the protocol in the trial

eFigure 54. Subgroup analysis of the types of viral respiratory infectious disease vaccines in immunogenicity.

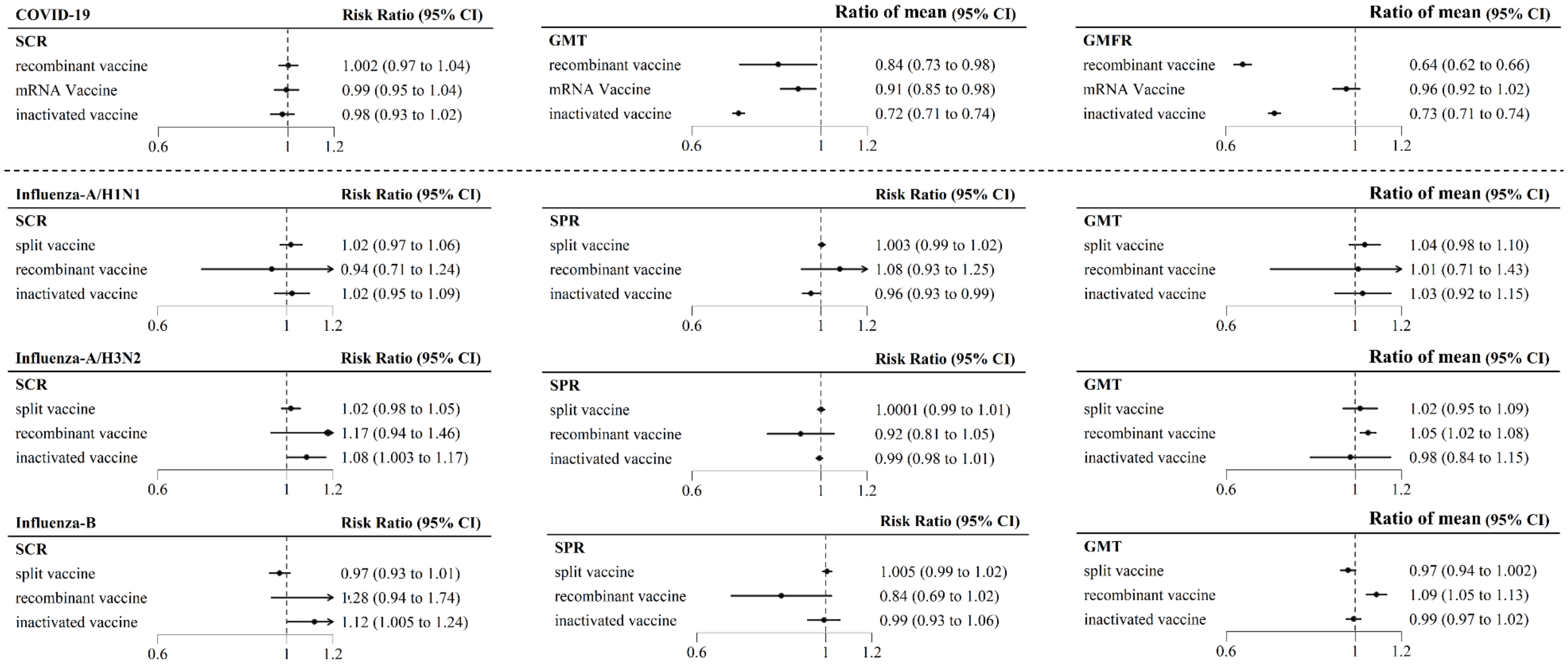

eFigure 55. Subgroup analysis of the types of viral respiratory infectious disease vaccines in SARS-COV-2 adverse event group

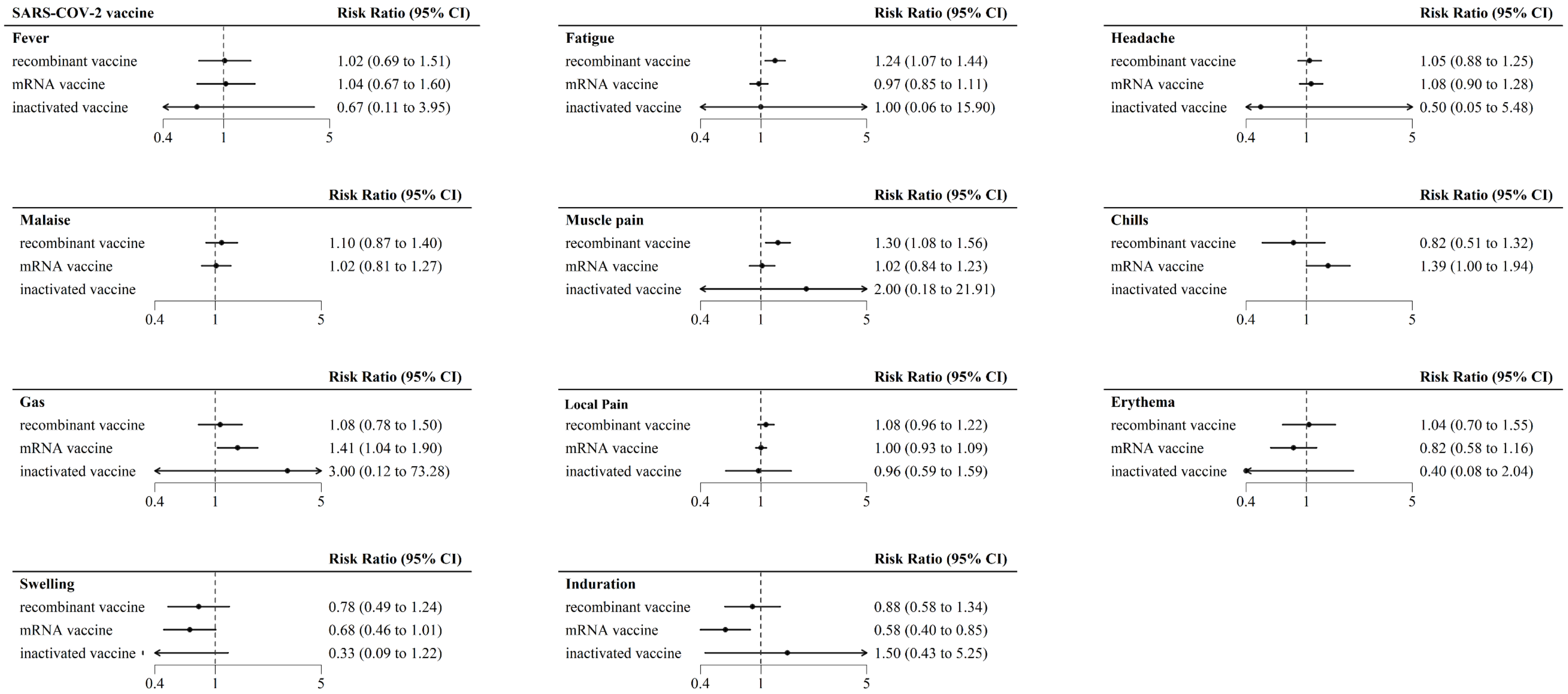

eFigure 56. Subgroup analysis of the types of viral respiratory infectious disease vaccines in seasonal influenza vaccine adverse event group

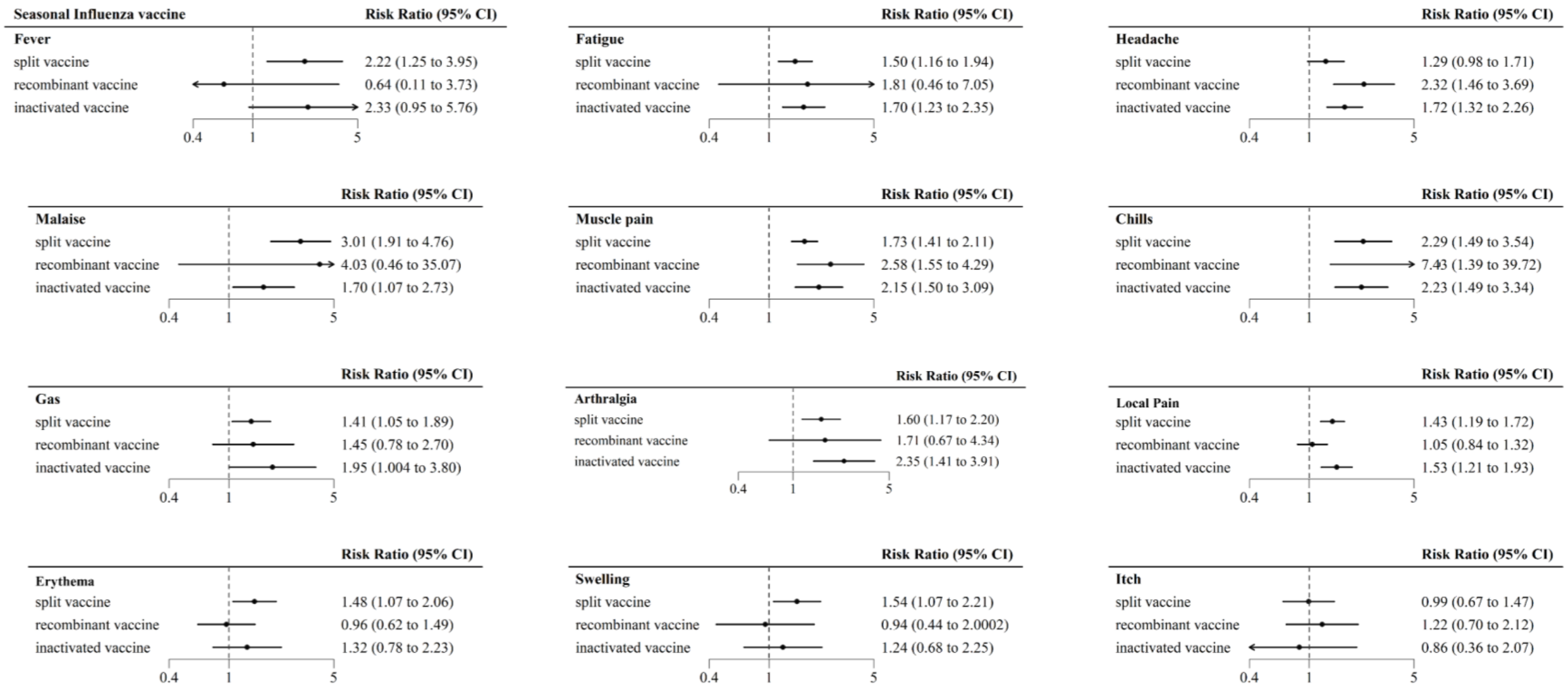

eFigure 57. Subgroup analysis of the types of concomitant administered vaccines in SARS-COV-2 and seasonal influenza vaccine immunogenicity group

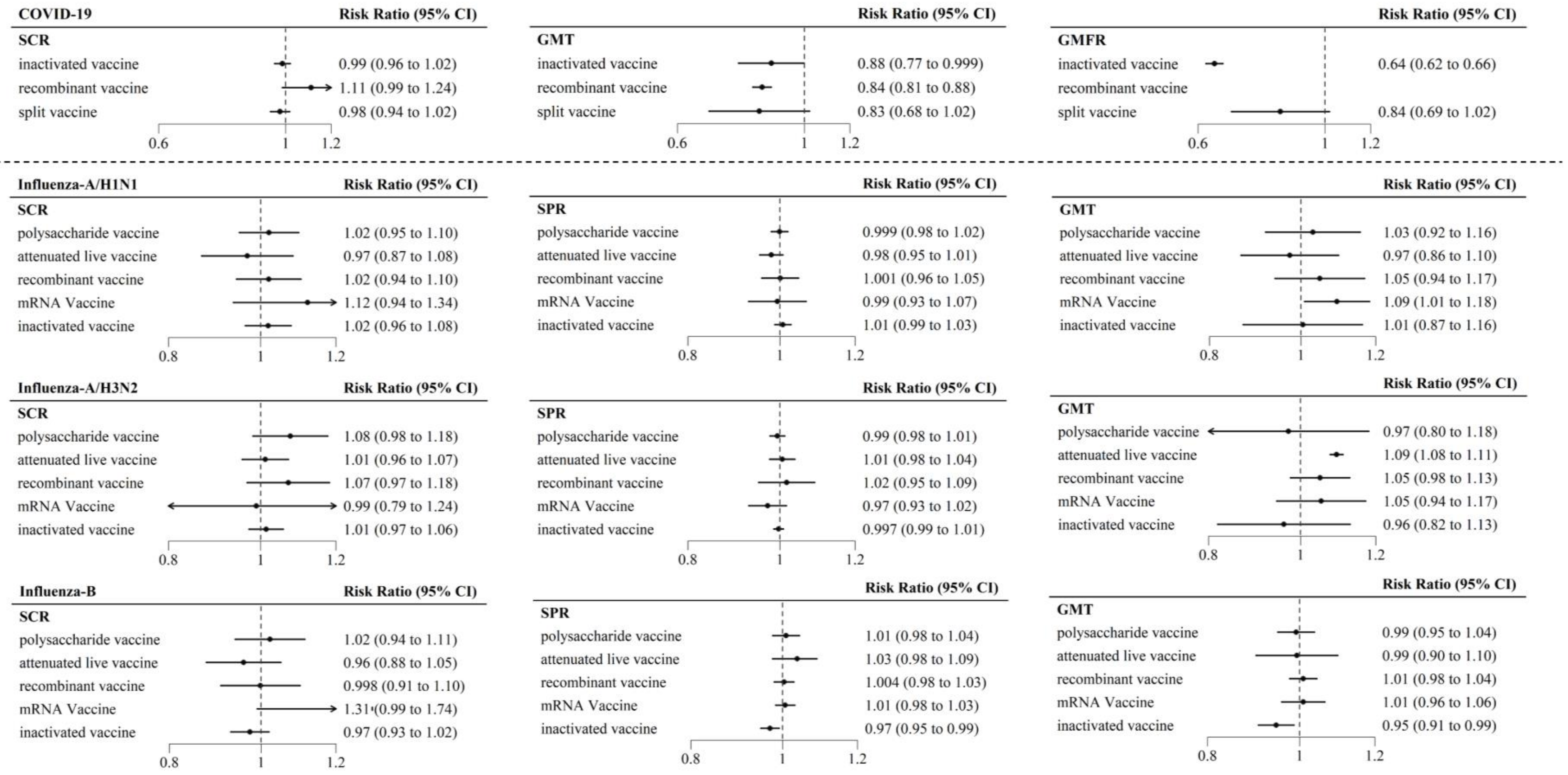

eFigure 58. Subgroup analysis of the types of concomitant administered vaccines in Seasonal influenza vaccine adverse event group

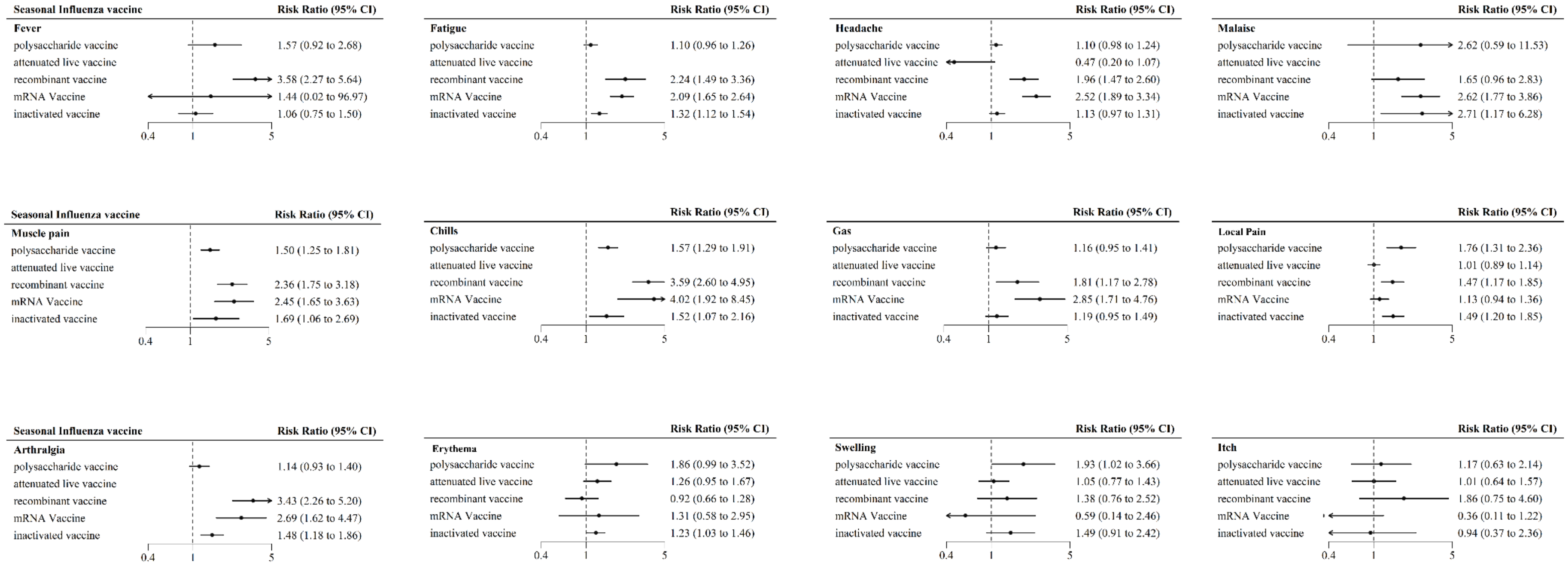

eFigure 59. Subgroup analysis of the types of concomitant administered vaccines in SARS-COV-2 adverse event group

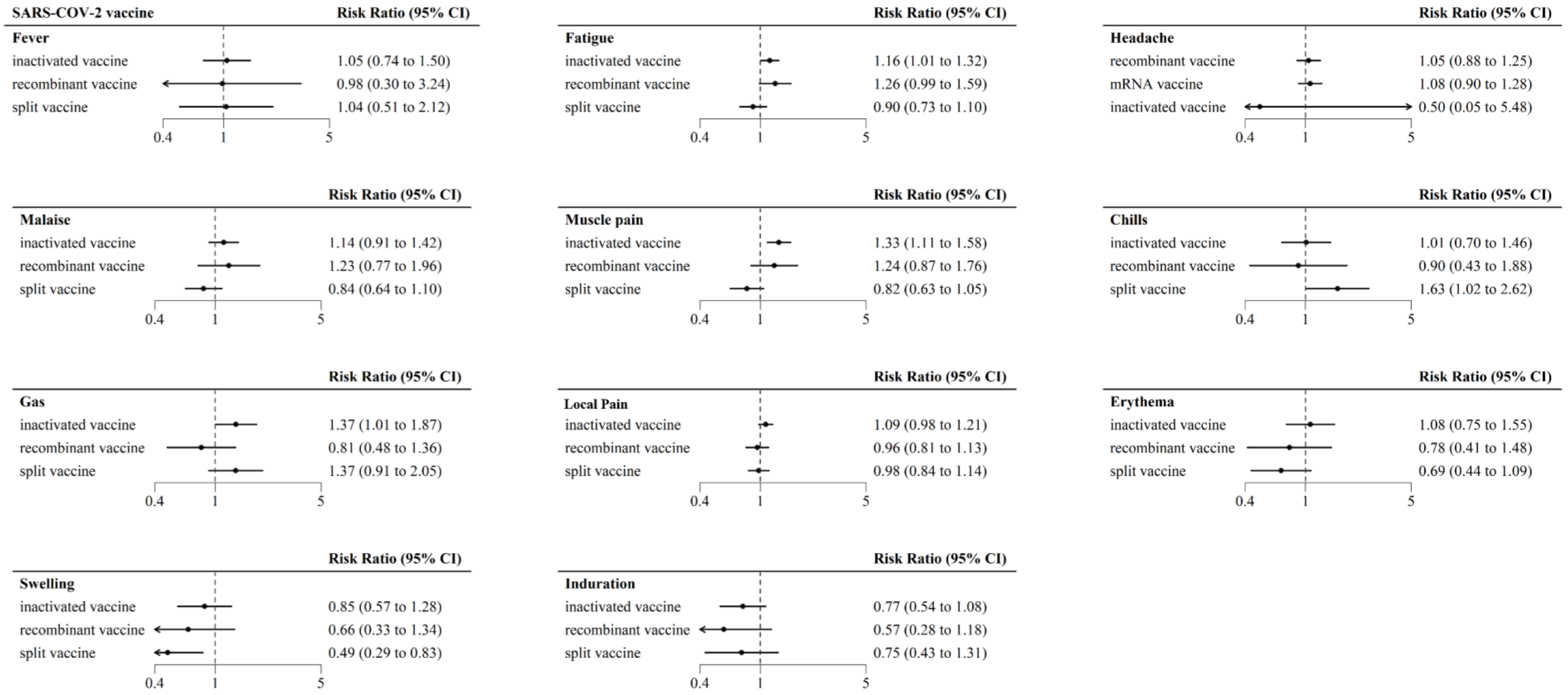

Supplement: Supplementary file 1 [file DataSheet_1.pdf]
